# The Monash Autism-ADHD genetics and neurodevelopment (MAGNET) project design and methodologies: a dimensional approach to understanding neurobiological and genetic aetiology

**DOI:** 10.1186/s13229-021-00457-3

**Published:** 2021-08-05

**Authors:** Rachael Knott, Beth P. Johnson, Jeggan Tiego, Olivia Mellahn, Amy Finlay, Kathryn Kallady, Maria Kouspos, Vishnu Priya Mohanakumar Sindhu, Ziarih Hawi, Aurina Arnatkeviciute, Tracey Chau, Dalia Maron, Emily-Clare Mercieca, Kirsten Furley, Katrina Harris, Katrina Williams, Alexandra Ure, Alex Fornito, Kylie Gray, David Coghill, Ann Nicholson, Dinh Phung, Eva Loth, Luke Mason, Declan Murphy, Jan Buitelaar, Mark A. Bellgrove

**Affiliations:** 1 Turner Institute for Brain and Mental Health, School of Psychological Sciences, Monash University, 18 Innovation Walk, Melbourne, VIC 3800 Australia; 2 Department of Paediatrics, Monash University, Melbourne, VIC 3800 Australia; 3 Department of Developmental Paediatrics, Monash Children’s Hospital, 246 Clayton Rd, Clayton, VIC 3168 Australia; 4 Murdoch Children’s Research Institute, Royal Children’s Hospital, 50 Flemington Rd, Parkville, VIC 3052 Australia; 5 Department of Paediatrics, Faculty of Medicine, Dentistry and Health Sciences, Royal Children’s Hospital, 50 Flemington Road, Parkville, VIC 3052 Australia; 6 Department of Mental Health, Royal Children’s Hospital, 50 Flemington Rd, Parkville, VIC 3052 Australia; 7 Neurodevelopment and Disability Research, Murdoch Children’s Research Institute, Royal Children’s Hospital, 50 Flemington Rd, Parkville, VIC 3052 Australia; 8 Centre for Educational Development, Appraisal, and Research, University of Warwick, Coventry, CV4 7AL UK; 9 Department of Psychiatry, School of Clinical Sciences, Monash University, 246 Clayton Rd, Melbourne, VIC 3168 Australia; 10 Faculty of Information and Technology, Monash University, Melbourne, VIC 3800 Australia; 11 Sackler Institute for Translational Neurodevelopment, Institute of Psychiatry, Psychology and Neuroscience, King’s College London, De Crespigny Park, Denmark Hill, London, SE5 8AF UK; 12 Department of Forensic and Neurodevelopmental Sciences, Institute of Psychiatry, Psychology and Neuroscience, King’s College London, De Crespigny Park, Denmark Hill, London, SE5 8AF UK; 13 Centre for Brain and Cognitive Development, Birkbeck, University of London, Henry Welcome Building, Malet Street, London, WC1E 7HX UK; 14 Department of Cognitive Neuroscience, Donders Institute for Brain, Cognition and Behaviour, Radboud University Nijmegen Medical Centre, Kapittelweg 29, 6525 EN Nijmegen, The Netherlands

**Keywords:** ASD, ADHD, Cognition, Genetics, Neuroimaging, Eye-tracking, HiTOP, RDoC

## Abstract

**Background:**

ASD and ADHD are prevalent neurodevelopmental disorders that frequently co-occur and have strong evidence for a degree of shared genetic aetiology. Behavioural and neurocognitive heterogeneity in ASD and ADHD has hampered attempts to map the underlying genetics and neurobiology, predict intervention response, and improve diagnostic accuracy. Moving away from categorical conceptualisations of psychopathology to a dimensional approach is anticipated to facilitate discovery of data-driven clusters and enhance our understanding of the neurobiological and genetic aetiology of these conditions. The Monash Autism-ADHD genetics and neurodevelopment (MAGNET) project is one of the first large-scale, family-based studies to take a truly transdiagnostic approach to ASD and ADHD. Using a comprehensive phenotyping protocol capturing dimensional traits central to ASD and ADHD, the MAGNET project aims to identify data-driven clusters across ADHD-ASD spectra using deep phenotyping of symptoms and behaviours; investigate the degree of familiality for different dimensional ASD-ADHD phenotypes and clusters; and map the neurocognitive, brain imaging, and genetic correlates of these data-driven symptom-based clusters.

**Methods:**

The MAGNET project will recruit 1,200 families with children who are either typically developing, or who display elevated ASD, ADHD, or ASD-ADHD traits, in addition to affected and unaffected biological siblings of probands, and parents. All children will be comprehensively phenotyped for behavioural symptoms, comorbidities, neurocognitive and neuroimaging traits and genetics.

**Conclusion:**

The MAGNET project will be the first large-scale family study to take a transdiagnostic approach to ASD-ADHD, utilising deep phenotyping across behavioural, neurocognitive, brain imaging and genetic measures.

**Supplementary Information:**

The online version contains supplementary material available at 10.1186/s13229-021-00457-3.

## Background

### Overview

Autism spectrum disorder (ASD) and attention-deficit/hyperactivity disorder (ADHD) are neurodevelopmental disorders affecting 1–2% and 5% of the population, respectively [175, 220]. ASD is defined by deficits in social communication, and restricted and repetitive patterns of behaviour and interests and altered sensory processing, whereas ADHD is defined by hyperactivity, impulsivity and inattention [16]. In ASD, 30–80% of cases exhibit ADHD symptomatology [186, 225], and 20–50% of ADHD cases display ASD symptoms [127, 234]. The introduction of the DSM-5 has allowed, for the first time, the concurrent diagnosis of ASD and ADHD, and the two disorders are now recognized to co-occur in up to 50% of cases [127, 271]. This comorbidity can be associated with a more severe ADHD phenotype and higher treatment needs overall [68, 305].

Although ASD and ADHD are diagnosed according to a symptomatic and behavioural presentation and developmental history, both conditions have a strong genetic aetiology and are highly heritable with estimates of up to 85% for ASD [304] and 70–90% for ADHD [104, 172]. Evidence of familiality comes from findings that first degree relatives of affected individuals often show subclinical behavioural or neurocognitive difficulties characteristic of ASD and ADHD [132, 205, 233, 243, 257]. Furthermore, siblings of children with ASD have a greater likelihood of having ADHD than the general population [116, 117], and siblings of children with ADHD exhibit greater ASD symptoms than healthy controls [197] suggesting shared familiality. It is becoming clear that multiple genes are implicated in ASD and ADHD and these are associated with multiple biological systems. The genetic links may also transcend diagnostic categories, with twin studies providing evidence for shared genetic liability for ASD and ADHD [116, 117]. There is also strong evidence for a degree of shared genetic aetiology [234], with population-based research suggesting that ASD and ADHD symptoms share common genetic variance throughout childhood and adolescence [270].

Copy number variations (CNV’s; [88, 290]), de novo mutations [143, 210, 230, 240], and common genetic variation from Genome-Wide Association Studies (GWAS; [113, 126]) are all implicated in the genetic aetiology of ASD. Similarly, CNVs [103], rare variants [129], and GWAS single nucleotide polymorphisms (SNPs; [83]) are implicated in the genetics of ADHD. Although there is a growing body of literature on the phenotypic, neurobiological and genetic overlap between ASD and ADHD [11, 127, 146, 234, 264], comprehensive transdiagnostic dimensional phenotyping approaches that are agnostic to diagnostic category are only beginning to emerge.

### The Research Domain Criteria (RDoC) and Hierarchical Taxonomy of Psychopathology (HiTOP): complementary frameworks for research in ASD and ADHD

Traditional taxonomies, such as the DSM and ICD, inherently assume segmentation between diagnostic categories. However, high rates of comorbidity between specific disorders, for example ASD and ADHD with intellectual disability [ID; 274, 278], obsessive compulsive disorder (OCD; [166, 183]), oppositional defiant disorder (ODD) and conduct disorder (CD; [32, 185]), and depression and anxiety [90, 268], as well as significant within disorder heterogeneity [106] challenge the assumption of a clear division between diagnostic categories. As the search for biological causes and accurate ways to identify psychiatric disorders gains traction, there is a move away from categorical conceptualisations of disorders towards a more dimensional understanding of psychopathology [60, 130, 239]. The National Institute of Mental Health’s (NIMH) Research Domain Criteria (RDoC) project [141, 142] and the Hierarchical Taxonomy of Psychopathology (HiTOP) consortium [158] represent complementary approaches to addressing the limitations of traditional categorical nosologies by using dimensional models of psychiatric and neurodevelopmental disorders evaluated at multiple levels of measurement. Studying phenotypes related to ASD and ADHD as hierarchically organised dimensions resolves problems associated with comorbidity and heterogeneity [105, 157].

The principal focus of RDoC is the analysis of dimensional phenomena at multiple levels of analysis across several core functional domains. These include behaviour, cognition, neural circuits, and genes in areas such as social processes and cognitive systems. These elements are organised within the RDoC matrix, which is primarily intended as a heuristic framework to encourage and facilitate psychiatric research that is unconstrained by traditional diagnostic categories. The primary focus of HiTOP is the articulation of the structure of the symptoms of psychopathology, which are conceptualised as hierarchically organised dimensions [158, 163]. These dimensions can be studied at varying levels of generality and specificity to uncover shared and unique genetic, neurobiological, and clinical correlates [65, 169, 291]. The RDoC and HiTOP approaches are thus complementary: the hierarchically organised phenotypic dimensions furnished by HiTOP provide the structural framework for exploring the functional domains and elements of the RDoC matrix [162, 188].

### Alignment of ASD and ADHD neurocognitive endophenotypes with the RDoC matrix

A number of neurocognitive traits have been identified as areas of difficulty for children with ASD and ADHD. Some neurocognitive domains are associated with similar levels of impairment across ASD and ADHD, while others appear to differentiate between them. Although atypical neurocognitive profiles are frequently observed at a group level, not all individuals within a disorder show divergence across all behavioural and neurocognitive domains. This heterogeneity has hindered clinical translation of group-level findings to individuals (e.g. [40, 59, 73, 76, 82, 102, 243, 280, 286]). Within ASD and ADHD, deficits are seen on tasks of *sustained attention* and *arousal* [20, 29, 54, 148, 148, 150, 150, 151, 289], *cognitive control,* for example inhibition (e.g. [21, 115, 221, 218, 300, 301]), *social processes* such as emotion recognition and/or theory of mind [14, 36, 128, 281], visual and verbal *working memory* [69, 120, 226, 227], and *reward sensitivity* and *decision-making* [147, 196, 309]. *Sensorimotor* abnormalities are common in ASD. In particular, oculomotor deficits are robustly associated with ASD [112, 149, 194], with emerging evidence for oculomotor impairments in ADHD [101]. These areas of neurocognitive divergence broadly align with five RDoC matrix domains: positive valence systems, cognitive systems, arousal/regulatory systems, social processes and sensorimotor systems [193, 199].

### Relationship between ASD and ADHD within HiTOP model

Although dimensional models of psychopathology originated in the developmental literature [3, 139], neurodevelopmental disorders are yet to be fully integrated into the HiTOP model [163]. The HiTOP framework conceptualises psychopathology as a multi-dimensional hierarchy, with an overarching factor for general psychopathology, or ‘*p*’ factor, represented at the top of the hierarchy and reflecting a common liability for mental disorder [48, 66]. Below this ‘*p*’ factor are super spectra; internalising, externalising, and psychosis; representing shared vulnerabilities to more specific ranges of problems. Internalising symptoms encompass depression, anxiety and somatic, eating and sexual difficulties, and subsumes the narrower and distinct subspectra of fear and distress. The externalising domain subsumes substance abuse and antisocial behaviours and is further differentiated into the disinhibited externalising (e.g. impulse-control problems) and antagonistic externalising (e.g. antisocial personality traits) subspectra. The psychosis super-spectra captures phenotypic variance related to psychotic disorders, but can be further differentiated into thought disorder (i.e. positive symptoms, experiences, and traits, such as reality distortion) and detachment (negative symptoms, experience, and traits, such as social withdrawal and emotional detachment).

To date, the full HiTOP model is more clearly articulated in adult populations [158, 163] and existing work examining the placement of neurodevelopmental disorders within dimensional models of psychopathology has been characterized by low specificity in focusing exclusively on broad band psychopathology factors (i.e., *p* factor, externalizing and internalizing superspectra [202, 228, 265]). Despite this work, there is still insufficient evidence to indicate the placement of neurodevelopmental disorders within HiTOP [157]. An advantage of the HiTOP conceptualization beyond general dimensional approaches in developmental psychopathology is that relevant phenotypes can be examined at varying levels of generality and specificity, from broad spectra, to subspectra, empirical syndromes, symptom components and maladaptive traits [158]. If neurodevelopmental disorders are to be included in the HiTOP framework, more evidence is needed to determine whether a general neurodevelopmental disorder subfactor [265] or alternatively, more granular hyperactive, inattentive [228], and social communication [202] subfactors, might best explain the relationship between psychopathology and neurodevelopmental conditions.

MAGNET is uniquely positioned to examine the relationship of ADHD and ASD to other forms of psychopathology within a hierarchical dimensional model, because phenotypes related to these conditions are being measured at a finer level of granularity compared to previous studies [202, 228]. For example, a dimension related to stereotyped behavior has yet to be incorporated into the HiTOP model [157]. The MAGNET protocol will be able to investigate the position of this phenotype using the Childhood Routines Inventory—Revised and Restricted Interests and Repetitive Behavior subscale of the Social Responsiveness Scale, 2nd Edition. Furthermore, studies that have examined the placement of ADHD and ASD within HiTOP have not taken into consideration potential phenotypic subdimensions or subtypes of ADHD and ASD [6, 201]. Finally, MAGNET is unique in concurrent measurement of RDoC-relevant constructs (e.g., cognition, genetics) compared to previous studies (e.g., [265]), which enables us to address method variance, validate the findings with objective cognitive assessment and observer ratings, as well as address the RDoC-HiTOP interface [188].

A conceptual cross-mapping between RDoC and HiTOP has previously been outlined [162, 188]. HiTOP spectra, subspectra, empirical syndromes, symptom components, and maladaptive traits form the phenotypic targets to which biologically informed RDoC constructs can be related [162]. Thus, there is synergy between these two complementary dimensional approaches to psychopathology. Leveraging the RDoC and HiTOP approaches has the potential to pave the way for a unified nosology, which is biologically informed and has clinical application [158, 169, 188, 212]. Symptom ratings, behavioural measures and neurocognitive tasks selected to measure psychopathology, and more specifically core ASD-ADHD traits, will allow for characterisation of ASD-ADHD within an integrated RDoC-HiTOP framework. However, although the RDoC-HiTOP interface has been broadly outlined, the relationships between specific HiTOP and biologically informed constructs has yet to be fully articulated and empirically tested at a fine-grained level of analysis [188].

The MAGNET study addresses several limitations of existing research. First, by incorporating multiple measures of ADHD and ASD symptomatology within the same structural modelling study, along with measures of other HiTOP spectra and subspectra (i.e., the Child Behavior Checklist), we are in a position to investigate the placement of these neurodevelopmental conditions, their potential subdimensions and/or subtypes, within a broader structural model of child psychopathology (i.e., internalizing, externalizing, thought disorder). Second, our methodology includes an array of measures that align with the constructs and subconstructs included in the RDoC matrix and across multiple levels of analysis, including behavioural assessment, cognitive paradigms, and genes. This uniquely positions the MAGNET study to address multiple potential points of convergence at the RDoC-HiTOP interface, as well as utilize the biologically-informed constructs of RDoC to test the validity of any ADHD/ASD phenotypic subdimensions or clusters. Of particular relevance is the cross-mapping of HiTOP dimensions and the RDoC Sensorimotor Process construct, for which there is currently no evidence [188]. The MAGNET study methodology includes multiple measures of Sensorimotor subconstructs, including Sensorimotor Dynamics (e.g., sinusoidal pursuit), Initiation (e.g., Reflexive saccades), and Inhibition and Termination (e.g., stop signal and antisaccade; [199]). Furthermore, we will be able to directly assess the mapping of Social Processes subconstructs, including Reception of Facial Communication and Understanding of Mental States, onto the Disinhibition Externalizing spectrum and overlap between this and the Detachment spectrum (i.e., ADHD and comorbid ASD/ADHD), which has not yet been investigated [188]. Our comprehensive cognitive battery aligns with multiple RDoC subconstructs, including Language, Limited Capacity, Inhibition/Suppression, and Interference Control, enabling us to test specific associations with symptom dimensions, as well as determine if cognitive profiles may differentiate subtypes [99]. Finally, we extend upon previous studies by including genotyping, enabling us to investigate the RDoC-HiTOP interface at the level of genes by calculating polygenic risk scores for ADHD, ASD, and related phenotypes [83, 126].

### Precision phenotyping to facilitate genetic discovery

Current studies attempting to uncover the neurobiological correlates of ASD and ADHD typically take one of two approaches. The first approach aims to recruit large sample sizes to facilitate high powered genetic analyses, with the trade-off being that only surface level phenotyping of behaviour is typically captured [239]. The second approach recruits smaller samples with deeper phenotyping using multiple modalities and informants, at the cost of reduced sample sizes, lower statistical power and greater financial expense per participant [239]. Large biobanking projects for ASD (e.g. SFARI, [1], Australian Autism CRC biobank, [8], and Norweigian Autism Birth Cohort [272]) and ADHD (e.g. International Multicentre ADHD Genetics [IMAGE] program; [10], The Lundbeck Foundation Initiative for Integrative Psychiatric Research [iPSYCH]; [83]) have achieved large sample sizes but only capture clinical symptom level data which provides minimal insight into the structure of developmental psychopathology and associated neurobiology. Other projects with more comprehensive phenotyping protocols including clinical, neurocognitive and imaging measures such as the EU-AIMS (LEAP) study [178], Biological Origins of Autism (BOA) study [269], and ENIGMA-ADHD/ASD [35, 136, 137]. However, these projects typically confine recruitment to DSM-5 categories for either ASD or ADHD, either excluding on the basis of comorbidity or not accounting for the effects.

There is increasing momentum toward transdiagnostic research efforts, as evidenced through projects such as the NIMH-funded Bipolar Schizophrenia Network on Intermediate Phenotypes (B- SNIP) project [58], which applied a dimensional framework to schizophrenia and bipolar disorder, as well as biobank initiatives like the Healthy Brain Network [7], and the Adolescent Brain Cognitive Development (ABCD) study [173]. Of these studies, the B-SNIP project is the only transdiagnostic study to our knowledge that incorporates first-degree relatives in its design. The inclusion of siblings of first degree relatives enhances the power to detect novel biomarkers with robust biological plausibility, relative to case–control designs, as relatedness is consistently known to reduce the amount of variance within outcome measures [50, 253].

Although there is consistent evidence suggesting shared genetic liability and family aggregation for ASD and ADHD, direct evidence for shared DNA variation from techniques such as GWAS or DNA has been harder to discern [12, 242]. One contributing factor to the inability of techniques such as GWAS to identify shared genetic variation between ADHD and ASD, for example, may be the coarseness of the phenotyping employed and imprecise mapping of comorbidities [42, 236]. A hierarchical approach to psychopathology and neurodevelopment, such as that proposed by HiTOP, whereby phenotypic variation is dimensionally measured in both cases and key comparison groups, and at multiple levels of measurement, provides more specific phenotypic targets for genetic discovery [291]. This circumvents the current difficulties that broad heterogeneous diagnostic categories pose for mapping the underlying genetic architecture of psychiatric illness [239, 291]. Looking outside of the confines of DSM-defined diagnostic categories for genetic associations using a hierarchical framework will allow for identification of both general and specific levels of genetic risk. Indeed, GWAS studies suggest pleiotropy is prevalent in psychopathology, with multiple genes and common genetic variation implicated across a number of disorders [275, 291]. Further, dimensional approaches confer substantially higher statistical power to detect trait-associated genetic variation [258, 259]. Case–control designs have the potential to weaken the genetic signal, with classification of subthreshold cases as controls increasing the breadth of phenotypic and genetic variability within groups [303]. To improve our mapping of the genetic architecture of the ASD-ADHD spectra, and neurodevelopment more broadly, we need to have greater precision in our studies of phenotype-genotype associations. Ways to achieve this can be through family designs, high precision phenotyping grounded in theoretically and biologically informed frameworks (e.g. HiTOP, RDoC), and comprehensive clinical review of all cases.

### The Monash Autism-ADHD Genetics and Neurodevelopment (MAGNET) project

To our knowledge there is no current study taking a truly transdiagnostic approach to understand the symptomatic, neurobiological (neurocognitive and neuroimaging) and genetic overlap between ASD and ADHD. A transdiagnostic sampling strategy that combines a family study design with deep dimensional phenotyping is needed across the ASD-ADHD spectra. By drawing on both RDoC and HiTOP frameworks, the MAGNET Project will contribute to our understanding of how neurodevelopmental disorders fit into a data-driven hierarchical taxonomy. Further, by understanding how these dimensional phenotypes present in families of children with ASD and ADHD, and whether siblings share similar behavioural signatures will provide crucial evidence for familiality of different ASD-ADHD phenotypes. The MAGNET Project therefore aims to: (1) identify data-driven symptom clusters across ADHD-ASD spectra using deep phenotyping of symptoms and behaviours; (2) investigate the degree of familiality for these data-driven symptom clusters; (3) map the neurocognitive and brain imaging correlates of these data-driven symptom clusters; and (4) explore their genetic correlates.

## Methods

### Study design

The MAGNET Project will enrol 1200 families with children aged between 4 and 18 years of age. Children who are typically developing, as well as those with a diagnosis of ASD, ADHD, or ASD + ADHD will be recruited to ensure both ends of the ASD-ADHD spectra are appropriately sampled. Children who are under investigation for ASD and/or ADHD and are referred to the study by their paediatrician will also be recruited. In addition, unaffected and affected siblings of probands will be recruited. A dimensional enhancement approach to sampling will be taken, as it augments clinical samples with non-clinical participants and those exhibiting subthreshold symptoms [75, 161]. This sampling strategy, whereby typically developing children are transdiagnostically phenotyped alongside probands and subthreshold cases (e.g. siblings, children currently under investigation for ASD or ADHD) combines the strengths and offsets the weaknesses of categorical and dimensional approaches to psychopathology research by increasing statistical power [50, 253] whilst maintaining clinical validity and enabling direct comparisons with existing diagnostic classifications systems [75, 131]. The MAGNET protocol comprehensively phenotypes all children and siblings, irrespective of case–control status, for behavioural and neurocognitive constructs that are central to ASD and ADHD symptomatology and align with RDoC and HiTOP frameworks (e.g. *internalising and externalising symptoms, attention and cognitive control, arousal, reward, working memory, perception, social processes,* and *sensorimotor processes*)*.* The battery uniquely captures dimensional traits across ASD-ADHD spectra using a range of symptom, parent-report, neurocognitive, and direct behavioural observation measures to capture the target domains from multiple perspectives. This approach will provide a rich source of data unconfounded by informant bias and method bias, with the opportunity to model the correspondence and complex interactions of information obtained from multiple informants [2, 176, 176, 177, 177, 213, 215, 219].

**Fig. 1﻿:**
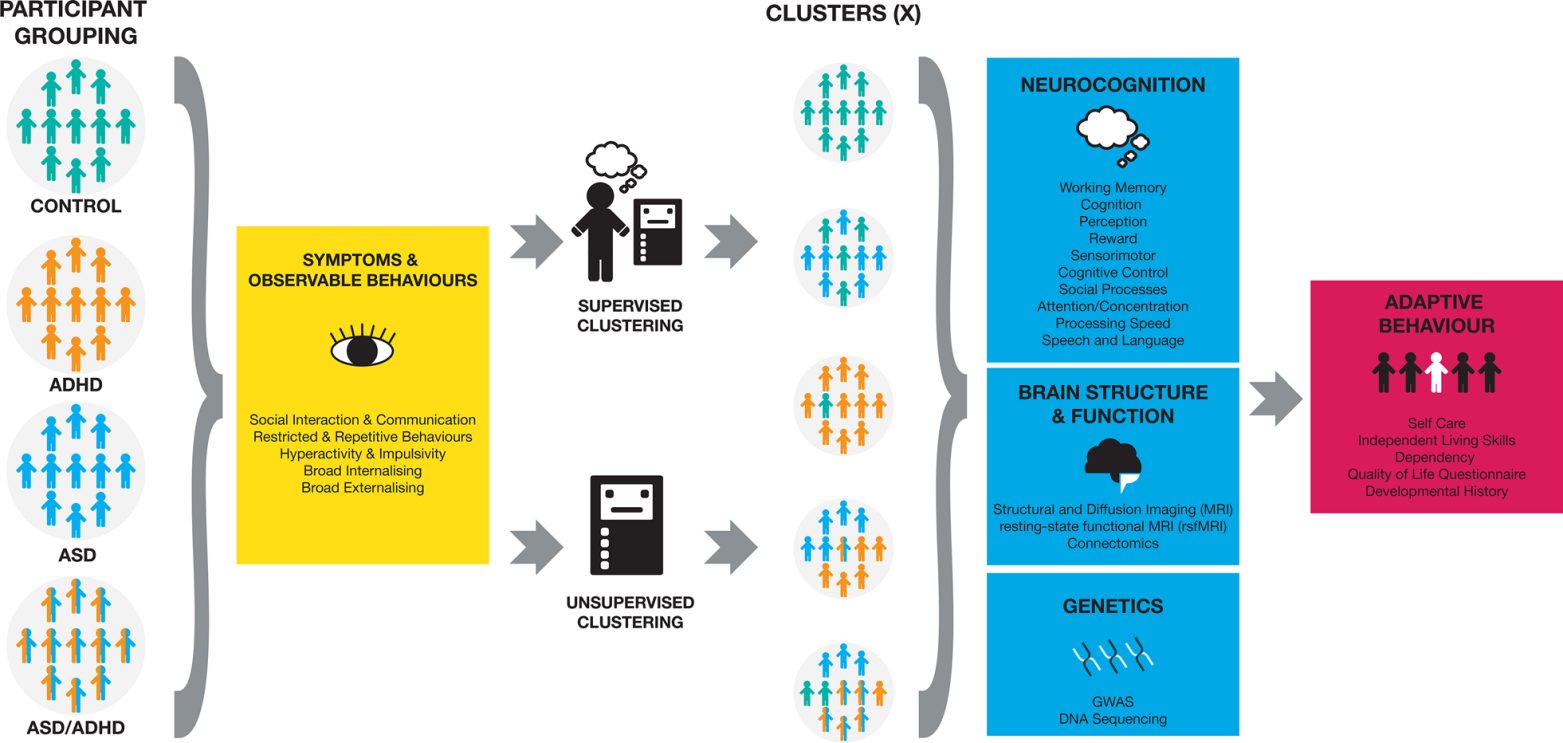
The MAGNET Project study protocol. **Participant** **grouping:** The MAGNET Project is a single-site study recruiting children who are typically developing, as well as those with elevated ASD, ADHD, or ASD + ADHD symptoms. Affected and unaffected siblings of probands will also be recruited. All children undergo comprehensive dimensional phenotyping across behavioural constructs central to ASD and ADHD target domains. **Clustering:** The MAGNET Project will use both supervised and unsupervised methods for discovery of ASD-ADHD clusters using measures of symptoms and behaviours. As these methods are hypothesis free and diagnosis naïve, the number of clusters will not be determined a priori. Uniquely, the battery captures target domains from multiple perspectives (self-report, parent-report, clinician rated measures, and direct child measures [e.g. eye-tracking and neurocognitive tasks]). All control children, probands, and siblings and parents of probands provide a saliva sample for genetic analysis. Structural and functional brain measures (magnetic resonance imaging [MRI], resting state MRI, and diffusion weight imaging) are also collected. The neurocognitive, brain imaging, genetic, and functional outcomes will then be mapped to the data-driven symptom clusters

Targeted sampling through hospitals, schools, private practice clinicians, and social media across Victoria, Australia will allow for a broad and representative distribution of socioeconomic status (SES) and symptom presentation. Currently the MAGNET Project is in an open recruitment phase. The MAGNET Project will actively recruit females and children with mild-severe intellectual disability, as these children are typically under-represented, or excluded from, studies of ASD and ADHD. The study has been piloted on control and clinical children aged 4 to 18 years of age (see Table 1 for preliminary demographic and clinical data) to assist in deciding appropriate age and cognitive ranges for tasks and minimum dataset requirements. See Fig. 1 for an overview of the MAGNET Project study protocol (see Additional file 1 for the MAGNET Project Protocol).

**Table 1:** Preliminary demographic and clinical data for the MAGNET project for *N* = 216 participants across controls, probands and siblings

	Controls	Probands	Sibling unaffected	Sibling affected	Total
Total	33	95	56	32	216
*Diagnosis*					
ASD	–	34	–	8	42
ADHD	–	25	–	7	32
ASD/ADHD	–	27	–	4	31
Sus. ASD/ADHD	–	9	–	13	22
*Sex*					
Male	15	69	18	17	119
Female	18	26	38	15	97

ASD, autism spectrum disorder; ADHD, attention-deficit/hyperactivity disorder; Sus., suspected; Age, average age in months

### Participant eligibility

Children with a diagnosis of ASD and/or ADHD provide the clinical report from their clinician with evidence of diagnosis. Children who are under investigation, or queried for, ASD and/or ADHD are required to have a clinician (paediatrician, psychologist, and/or general practitioner) currently managing their care. Siblings of probands must share two biological parents with the proband. The healthy control children are required to have no neurodevelopmental diagnosis, no first-degree relative with a diagnosis of ASD and/or ADHD, and no history of a psychiatric (e.g. depression or anxiety) or neurological illness (e.g. head injury or tuberous sclerosis).

Probands and siblings with comorbidities such as anxiety, depression, oppositional defiant disorder (ODD), conduct disorder (CD), and obsessive–compulsive disorder (OCD) are not excluded. As a large proportion of children with ASD and ADHD experience these comorbid disorders [79, 183, 306], exclusion of these disorders may result in a sample that is not representative of the target population. Where possible, one or both biological parents complete a battery of questionnaires examining ASD and ADHD symptomatology, mental health, and quality of life. Exclusion criteria for all children include known genetic (e.g. Fragile X, Angelman’s Syndrome, tuberous sclerosis) or environmental (e.g. traumatic brain injury, foetal alcohol syndrome) causes. A peri/prenatal environment questionnaire retrospectively captures maternal alcohol and drug use, medication, illness/infection, and complications during the pregnancy and delivery. Retrospective information on the child’s development, including developmental milestones and regression is obtained via parent-report. As the questionnaire battery is extensive, at least one parent/caregiver is required to speak English. Parents complete approximately 3 hours of online questionnaires, and one (control families) or two (clinical families) 3-hour research visits at Monash University to complete the testing protocol.

All children undergo case review by a registered psychologist and paediatrician, and speech pathologist if available, to determine a ‘best clinical estimate’ of that child’s current diagnostic status. A best clinical estimate will be given for ASD, ADHD, comorbid ASD/ADHD, intellectual disability (ID), CD, and ODD (see Additional file 2). The best clinical estimate will not be used as exclusion criteria for the study. Children who do not meet thresholds for ASD and/or ADHD will still provide useful information about the dimensionality of ASD and ADHD symptoms. Children with an estimated full-scale intelligence quotient (FSIQ) in the range for ID (IQ ≤ 70) as measured using standardised psychometric assessment (e.g., Wechsler assessments) are administered a minimum dataset protocol (see Additional file 3), but will attempt additional tasks from the battery wherever possible.

#### Ethnicity

Single-nucleotide polymorphisms (SNPs) may vary between ethnic populations and potentially cause false positive results in genetic association studies. To avoid the potential impact of population stratification only children with four grandparents of European ancestry are invited to complete the genetic component of the protocol.

#### Siblings

Only full biological siblings will be eligible to take part in the study. Within simplex families, that is, families where only one child has an ASD and/or ADHD diagnosis, the child with the ASD/ADHD diagnosis is nominated as the proband. In multiplex families, families where more than one child has an ASD and/or ADHD diagnosis, the eldest child is denoted as the proband and younger children are designated as affected or unaffected siblings. Unaffected siblings of ASD/ADHD probands have no diagnosis of ASD/ADHD, are not under investigation for ASD/ADHD, and are not assigned a neurodevelopmental disorder diagnostic category during their best clinical estimate review.

#### Medication

The child’s current and previous medication history, medication prescriber (e.g. paediatrician, general practitioner), and reasons for any medication changes, will be recorded.

Children who are taking medication remain on their medication during Visit 2 when their relevant Wechsler and Autism Diagnostic Observation Schedule—Second Edition (ADOS-2) assessments are completed (see Additional file 4 for clinical assessment protocol). However children taking stimulant or non-stimulant medication for ADHD including methylphenidate, lisdexamfetamine, or dexamfetamine are required to withdraw from their medication 48–72 hours prior to completing the neurocognitive test battery during Visit 1 [51, 195]. Participants taking guanfacine or antipsychotics (e.g. risperidone, aripiprazole) do not withdraw for any component of the protocol as abrupt withdrawal from these medications may be associated with adverse side effects [138, 273, 307]. Children taking melatonin are not required to withdraw prior to participating.

### Phenotyping overview

Each of the measures or tasks included were selected as gold standard measures that are widely used, have biological plausibility, and show robust effect sizes when differentiating controls from either ASD or ADHD (See Table 2 for the MAGNET Project symptom and environmental phenotyping measures, and Table 3 for neurocognitive phenotyping measures).

**Table 2:** The MAGNET project symptom phenotyping measures

Tasks	Attention	Working memory	Speech and language	Social Processes	Cognitive control	Reward	Sensori-motor	Perception
WISC-V/WPPSI-IV/WAIS-IV/WAIS-II	X	X	X		X	X	X	X
*Dimensional measures of ASD traits*								
ADOS-2 + 3DI	X		X	X	X			
Childhood & Adult Routines Inventory				X				
Autism Quotient	X			X	X		X	X
Social Responsiveness Scale			X	X			X	
*Dimensional measures of ADHD traits*								
Conners’ Parent Rating Scale—Revised	X	X		X	X	X		
SWAN	X	X				X		
Scale of Attention in Intellectual Disability	X	X				X		
*Comorbid Symptoms*								
Aberrant Behaviour Checklist		X				X		
Child Behaviour Checklist			X	X				
DAWBA	X		X	X	X	X	X	
Children’s Communication Checklist 2			X	X				
CELF-5	X	X	X	X	X		X	X
CELF-P2	X	X	X		X		X	X
PLS-5	X	X	X		X			
PEP-3	X	X	X	X	X		X	X
Strengths and Difficulties Questionnaire	X			X		X		
Spence Childhood Anxiety Scale				X				
Childhood Depression Inventory				X				
Beck Depression Inventory				X				
Beck Anxiety Inventory				X				
*Domain general rating scales*								
Child Health and Illness Profile			X	X				X
WHO Quality of Life Questionnaire			X	X				X
Vineland Adaptive Behaviour Scale	X	X	X	X	X		X	X

WISC-V =Wechsler Intelligence Scale for Children—Fifth Edition; WPPSI-IV =Wechsler Preschool and Primary Scale of Intelligence—Fourth Edition; WAIS-IV = Wechsler Adult Intelligence Scale—Fourth Edition; SWAN =Strengths and Weaknesses of ADHD symptoms and Normal Behaviour; DAWBA = Development and Well-Being Assessment; CELF-5 =Clinical Evaluation of Language Fundamentals—Fifth edition; CELF-P2 =Clinical Evaluation of Language Fundamentals—Preschool-2; PLS-5 =Preschool Language Scales—Fifth Edition; PEP-3 =Psychoeducation Profile—Third Edition; WHO =World Health Organisation

**Table 3:** The MAGNET project neurocognitive phenotyping measures

Neurocognitive tasks	Attention	Working memory	Speech and language	Social processes	Cognitive control	Reward	Sensori-motor	Perception
Go/No-Go	X				X			
Stop signal task	X				X		X	
Reflexive saccade task							X	X
Anti-saccades	X		X		X		X	X
Sinusoidal pursuit	X						X	X
Step-ramp pursuit							X	X
Spatial working memory	X	X						X
Probabilistic reversal learning	X	X			X	X		
Cambridge gambling task	X				X	X		
Facial recognition task	X	X		X				X
Karolinska directed emotional faces			X	X				X
Reading the mind in the eyes	X		X	X				X
Continuous false belief task			X	X				

The components of the MAGNET protocol intended to measure phenotypic dimensions relevant to ASD were chosen in consultation and collaboration with the European Autism Interventions—A Multicentre Study for Developing New Medications—Longitudinal European Autism Project (EU-AIMS [LEAP]) study team [144, 178]. The EU-AIMS (LEAP) study is a European multi-centre study that aims to identify risk factors contributing to differences in brain development, social difficulties and other core ASD symptoms. Through aligning parts of the MAGNET and EU-AIMS (LEAP) protocols, the MAGNET project will also act as a replication site for the EU-AIMS (LEAP) study. The addition of measures for dimensional phenotyping of ADHD symptoms and relevant neurocognitive traits are unique to the MAGNET project and make ours the first large-scale family-based project to take a truly transdiagnostic approach to understanding ASD and ADHD (see Additional file 1 for MAGNET protocol summary).

### Characterisation of ASD, ADHD and comorbid symptoms

Dimensional ASD symptomatology is measured through parent-report measures capturing social communication (Autism Quotient—Child [AQ-C], [17], Child Communication Checklist—Second Edition [CCC-2], [33], Social Responsive Scale—Second Edition [SRS-2], [64]), social competence (Child Behaviour Checklist [CBCL], [4], restricted, repetitive, and stereotyped behaviours (SRS-2, [64],The Childhood Routines Inventory—Revised [CRI-R], [96]), and autism symptomatology overall (AQ-C; [17], SRS-2; [64]). Dimensional traits central to ADHD are captured through parent-report questionnaires, and an in-house observation checklist for ADHD behaviours completed during ADOS-2 coding. Parent rated measures of attention and inattention (Strengths and Weaknesses of ADHD Symptoms and Normal Behaviour [SWAN]; [13], Conners’ Parent Rating Scale—Revised [CPRS-R]; [62], Development and Wellbeing Assessment [DAWBA], [122]), hyperactivity (Aberrant Behaviour Checklist [ABC], [9], Strengths and Difficulties Questionnaire [SDQ], [121], DAWBA, [122]), impulsivity, and overall ADHD symptomatology (CPRS-R, [62]), are comprehensively assessed, alongside an additional measure of attention appropriate for children with intellectual disability (Scale of Attention in Intellectual Disability [SAID], [110]). Teachers are invited to complete the SRS-2, SDQ, and Conners’ Teacher Rating Scale—Revised [63], although completion rates are typically lower than for parent report. Height, weight, head circumference, and joint mobility and hypomobility [26] are also recorded for every child.

#### Comorbidities

Comorbidities commonly observed in ASD and ADHD are captured in all children, including anxiety (Child Behaviour Checklist [CBCL], [5], Spence Children’s Anxiety Scale [SCAS], [263]) and depression (CBCL, [5], DAWBA, [122], Childhood Depression Inventory—Second Edition [CDI-2], [159, 260]). Conduct problems and oppositional defiant problems are also indexed (CBCL, [5], CPRS-R, [62], DAWBA, [122]). Level of current cognitive function is determined using age appropriate Wechsler intelligence scales [293, 294, 295, 296]. See Additional file 4 for clinical assessment protocol.

### Adaptive behaviours and quality of life

Adaptive behaviour (Vineland Adaptive Behaviour Scale—Third Edition [VABS-3]; [262]) and quality of life (Child Health and Illness Profile—Child Edition [CHIP-CE], [229]) are measured in all children through parent-report questionnaires.

#### Language assessment

Language profiles in ASD are heterogeneous, ranging from non-verbal [114] to superior linguistic abilities [154]. Although language impairments are not a hallmark diagnostic criteria for ADHD, both linguistic and pragmatic deficits are commonly part of the symptom presentation [27]. Recent empirical records on the co-occurrence of language impairments in ASD and ADHD have identified impairments in structural and pragmatic aspects of language in both the groups [19, 164, 203, 250]. Despite the presence of language difficulties in ASD and ADHD, and indeed, in a number of other neurodevelopmental disorders and psychopathology, language constructs are not currently included in RDoC or HiTOP frameworks. Thus, the inclusion of language assessments in the MAGNET Project protocol will provide a novel and unique contribution to these nosologies.

A standardised screening measure for language difficulties (Clinical Evaluation of Language Fundamentals—Fifth Edition [CELF-5]: Screening Test; [297]) is administered to all enrolled children over 5 years of age. Children with a diagnosis of ASD and/or ADHD or those who are under investigation for these disorders, and control children who fall below criterion on the screening measure for language difficulties, are administered the Australian adaptation of the Clinical Evaluation of Language Fundamentals—Fifth Edition (CELF-5; Age group 5 to 21 years; [70], [298]) or Clinical Evaluation of Language Fundamentals—Preschool Edition (CELF-P2; Age group 3 to 6 years 11 months; [251]). This clinician-administered assessment provides a comprehensive global measure of language abilities, and characterises structural and pragmatic language in children.

The Preschool Language Scale—Fifth Edition (PLS-5; [310]) is administered to younger minimally verbal children. The PLS-5 incorporates information from clinical observation, direct measurement and parent report to assess domains of attention, play, gesture, vocal development, social communication, semantics, language structure, integrative language skills and emergent literacy skills in children from birth to 7 years 11 months. A caregiver rated questionnaire, the Children's Communication Checklist 2 (CCC-2; [33]), measures both structural (language form/content) and pragmatic traits of communication impairment in children. The CCC-2 includes an overall measure of communication skills and a Social Interaction Deviance Composite (SIDC) which indexes the strength of relationships between the social domains of communication and structural components of language, thereby aiming to attain and identify traits associated with pragmatic language difficulties. With poorer overall language performance and SIDC linked to ASD traits [33], these measures provide valuable information when differentiating comorbid presentations of language impairment in neurodevelopmental disorders. The SRS-2 also provides a parent-reported index of social communication. Recordings from the ADOS-2 provide high-resolution natural speech and language samples. See Additional file 4 for clinical assessment protocol.

### Measures of neurocognition

We assess the domains of *sustained attention, inhibition, cognitive control, arousal, reward, working memory, perception, social processes,* and *sensorimotor processes* with the view to utilise neurocognitive data to discover neurobiological correlates of novel ASD-ADHD data-driven clusters. The tasks chosen are widely used, have biological plausibility and show robust effects sizes when differentiating clinical cases from controls. See Table 2 for the MAGNET Project neurocognitive phenotyping measures. Additional file 5 summarises the MAGNET Project’s neurocognitive assessment protocol.

#### Neurocognitive tasks

The neurocognitive tasks will be completed on a desktop computer and touchscreen laptops. Amsterdam Neuropsychological Tasks (ANT), Psytools, PsychoPy and STOP-IT software programmes were used for task administration [261, 81, 214, 282, 283].

#### Response inhibition, sustained attention and cognitive control

Response inhibition refers to the ability to withhold or cancel a motor response [52]. Sustained attention, or vigilance, can be defined as the ability to maintain engagement in a task over a prolonged period of time [109]. This component of attention is thought to be mediated by top down, or endogenous processes, and is controlled by internal goals [192]. These cognitive functions are measured using a Go/No-Go (ANT; [261]) and Stop Signal Task which are standard measures of top-down/endogenous sustained attention and response inhibition. Response inhibition is indexed through stop-signal reaction time (SSRT) and the percentage of failed attempts to inhibit a response on tasks. Longer stop signal reaction times and commission errors indicate poor inhibition and more omission errors and are indicative of poorer sustained attention [282]. Response inhibition and sustained attention deficits are central to the conceptualisation of ADHD [20, 28, 221, 300, 301], with some support for deficits in ASD [21, 54, 152, 246, 218]. Further, these deficits are heritable, with unaffected siblings of ADHD probands demonstrating response inhibition and sustained attention difficulties [53, 111, 243, 257]. Similarly, reduced inhibitory control has been demonstrated to be familial in ASD families [246].

#### Arousal

Arousal can be understood as an individual’s state of reactivity, and although arousal is intimately linked with constructs like attention, the neural correlates of these processes are largely distinct [72]. Arousal will be examined by deriving measures of intra-individual variability in response times across tasks of sustained attention and response inhibition, as suboptimal arousal is thought to underpin intra-individual variability in ADHD [28, 29, 49, 254]. Increased response time variability is a hallmark feature of neurocognitive performance in ADHD [29, 148, 150, 151, 256] and is familial [165, 200]. Variability in response time is thought to be a marker for dysfunction in the frontal areas of the brain [30, 181], which is consistent with theories of hypo-arousal and fronto-striatal dysfunction in ADHD [74, 241]. Although children with ASD show similar response time variability to typically developing children [151], variability in response time appears to index ADHD symptomatology across diagnostic boundaries as children with comorbid ASD and ADHD show similar variability to those with ADHD [279]. Thus, response time variability as a proxy measure for arousal shows promise for effectively stratifying children with ASD, ADHD and ASD-ADHD.

#### Reward sensitivity

*Reward sensitivity* refers to the tendency to respond more strongly to incentives, or rewards, and is a process implicated in decision making. ADHD is associated with divergent *decision making*, differing sensitivity to *reward*, and elevated risk-taking behaviour [80, 147, 180, 309]. Effect sizes for decision making difficulties are comparable to the attention difficulties seen in ADHD [196]. Altered reward processing in ADHD is well-studied, and posited as central to the disorder [277]. Children with ADHD show poorer decision making as they have difficulty adjusting their responses in the face of changing levels of risk [59, 125, 276]. Biological plausibility is evidenced with correlative neuroimaging in ADHD of under activation in brain regions associated with decision making (i.e. ventral and dorsolateral prefrontal cortex, and insula; [39, 94] and hypo-responsiveness in neural circuitry involved with reward anticipation (i.e. ventral striatal circuitry; [245]). Dopamine is one of the neurotransmitters implicated in decision making and reward, and indeed, dopamine deficiency is a leading hypothesis in ADHD [309]. Together, a task engaging decision making, reward sensitivity, and risk-taking behaviour is a well-positioned ADHD trait for discovery of clusters.

In ASD, there is evidence for aberrant reward processing, but to a lesser extent than that observed in ADHD [156, 277]. Children with ASD showed increased activation in the anterior cingulate cortex during reward achievement compared to controls [247]. This region is thought to be involved with self-monitoring of performance in line with reward feedback [34, 232] and risk assessment [41]. However, there is some evidence to suggest ASD and control groups perform similarly on goal-directed decision making tasks in the context of explicit reward [100] and have similar sensitivity to monetary reward [85, 266], with no difference in neural activation while processing reward [168]. The less definitive evidence in ASD may indicate that only a subgroup of these children may in fact have altered reward processing and decision making.

To assess decision making, reward sensitivity, and risk-taking, the New Cambridge Gambling Task [44] will be used. It allows for delineation of risk-taking behaviours from impulsivity, and explicitly states the probability for each trial. Unlike other gambling tasks (e.g. Iowa Gambling Task), explicit statement of probability reduces the working memory load, thus reducing confounds of additional working memory deficits.

#### Probabilistic reversal learning

Broadly, cognitive flexibility is a component of executive function that encompasses adaptability at a behavioural level and is studied from a variety of perspectives such as set shifting, task-switching, and reversal learning [67]. More specifically, contingency-related cognitive flexibility is the adaptation of behaviour after negative feedback, typically measured using probabilistic reversal learning paradigms. In typical development, contingency-related cognitive flexibility specifically is associated with the orbitofrontal cortex, parietal cortex, and subcortical connections [107]. Impairments in contingency-related cognitive flexibility are seen in ASD [69, 91] and ADHD probands [145, 299], with impairments also observed in unaffected first degree relatives of ASD probands [246]. In ASD, cognitive inflexibility has been associated with restricted, repetitive, and stereotyped behaviours [91, 174, 190]. Neuroimaging findings demonstrate aberrant activation of networks during cognitive flexibility tasks in children with ASD (lateral frontoparietal and midcingulo-insular networks; [280]) and fronto-striatal function, which is implicated in cognitive flexibility, is thought to be impaired in ADHD [47, 89]. In the MAGNET Project contingency-related cognitive flexibility will be measured using a probabilistic reversal learning paradigm with positive and negative feedback [178, 209]. The number of trials required to shift to a new response choice, perseverative errors, and regressive errors index cognitive inflexibility.

#### Working memory

Internationally, the definitions of working memory are contentious, with working memory and short-term memory sometimes still used interchangeably. Some conceptualise working memory as the process of holding information in the mind for a short period of time, which can also be thought of as short-term memory [118]. Others understand working memory, also referred to as executive memory, as the ability to maintain and manipulate information, where this manipulation may have low or high executive demands [18, 77]. Tasks are then modality specific, using verbal or visual stimuli. The MAGNET Project’s conceptualisation of working memory aligns with executive memory that has high and low executive demands. Verbal and visual working memory difficulties are seen in both ASD and ADHD [87, 153, 184, 248, 249, 252], with deficits becoming more pronounced as the cognitive load increases [226, 227, 252, 267, 284]. These difficulties on working memory tasks with higher cognitive load correspond with atypical neural processing in children with ASD [222, 284], providing biological plausibility for working memory performance as a neurocognitive marker of ASD. Further, unaffected siblings of children with ASD and ADHD showed more impaired verbal and visuospatial working memory performance than typically developing controls [31, 204, 252]. The verbal and visuospatial working memory divergence seen in unaffected siblings of children with ASD and ADHD positions working memory as a good candidate endophenotype [108, 191]. Verbal [292, 293, 297, 298] and visuospatial [45, 46] working memory tasks which increase in cognitive load across trials allows us to index working memory capacity across the broad range of cognitive abilities captured in the study.

#### Social processes

*Emotion recognition* is the ability to correctly identify another person’s emotion based on their facial expression and is crucial for effective social communication. Emotion recognition difficulties in children with ASD are a consistent and robustly replicated finding [128, 281]. Atypical processing of emotions is also thought to be familial, with unaffected relatives of individuals with ASD also showing less severe, but still significant emotion recognition difficulties [78, 206]. Although emotion recognition is not as extensively researched in ADHD, there is some evidence for emotion recognition divergence in these children [14, 36, 84, 287]. Emotion recognition in the MAGNET project is conceptualised, and measured, as the ability to recognise both simple and complex emotional states (Reading the Mind in the Eyes Task [RMET]; [22]; Karolinska Directed Emotional Faces [KDEF]; [119]).

*Theory of Mind (ToM)* is the ability to understand and attribute mental states to oneself and to others and understand that others can have different mental states to yourself. Profound difficulties with understanding others’ thoughts and intentions in day-to-day life are common in ASD [216]. These difficulties with ToM have been linked to genetic anomalies associated with ASD [231]. False-belief tasks are widely used for assessing ToM and individuals with ASD typically show egocentric biases when completing these tasks compared to their typically developing peers [25]. These difficulties are less definitive in high functioning individuals with ASD however, with some able to successfully complete continuous false-belief tasks [244]. The ability of such a task to separate different individuals with ASD positions it well to stratify these individuals. Conversely, the findings within ADHD are currently heterogeneous. More research is necessary to understand whether these deficits are present in only a subset of these children [217].

### Oculomotor measures

Saccade and pursuit eye movement abnormalities have the potential to reliably distinguish ASD and ADHD children from controls [149]. Oculomotor abnormalities can arise as the result of abnormalities in a range of well-mapped neural circuitry throughout the brain, spanning motion sensitive visual area V5, parietal and frontal areas supporting visual attention and sensorimotor transformation, basal ganglia, brainstem and cerebellar circuitry [149]. Oculomotor control is ideal to measure in children, as it is quick, and affords sensitive, high-resolution recording, and requires minimal-to-no language comprehension for children to perform. Sensorimotor measures from ocular motor tasks include accuracy, motor dynamics (e.g. velocity profiles), initial eye acceleration in response to the onset of a visual target or target movement and integration of visual feedback in motor responses. The anti-saccade task, completed in children eight years and over, also provides a measure of how attentional processes and inhibition interface with oculomotor control [97, 140, 155, 198]. Other studies in schizophrenia and bipolar disorder have found unique relationships between genes associated with nervous system development and function and with sensorimotor processing and pursuit maintenance [171]. See Additional file 6 for oculomotor testing protocol.

### Brain structure and function

Large-scale neuroimaging studies have identified robust structural differences associated with ASD and ADHD, demonstrating both common and disorder-specific brain alterations. In both ASD and ADHD, cases showed reduced subcortical volumes [134, 235] and cortical thinning in temporal regions [136, 235]. Reduced surface areas were specific to ADHD [136], whereas ASD showed increased cortical thickness in frontal regions [235]. Evidence regarding differences in diffusion weighted imaging (DWI) and resting state fMRI (rs-fMRI) are based on smaller studies demonstrating wide-spread alterations in fractional anisotropy [86, 98] and less consistent changes in rsfMRI [170, 308].

Structural and functional brain imaging (resting state fMRI) will be collected to determine if neurobiological differences exist as a function of symptom-based data-driven clusters. All scans will be performed using Siemens Skyra 3 T scanner following previously established protocols [207, 237]. Data processing pipelines will include extensive correction for in-scanner motion [207, 211] which is the most prevalent MRI artefact in paediatric populations.

### Genetics

Saliva is collected from all probands, affected and unaffected biological siblings, biological parents of probands, and healthy controls for DNA extraction (see Additional file 7 for DNA collection and extraction protocol). DNA will be subjected to array-based genotyping (e.g. Illumina Global Screen Array for GWAS) and/or whole genome sequencing, as funding allows. Because our study sample size has limited power to reliably detect novel associations with DNA variants, we will capitalise on existing publicly available data and consortia science in the following ways. First, we will derive Polygenic Risk Scores (PGRS; [55, 95]) for ASD and ADHD using international datasets as the base dataset (e.g. Psychiatric Genomic Consortium [PGC] and iPSYCH; [83, 126]) and our entire sample of probands as the target dataset. We will estimate the relationships between polygenic risk scores for ADHD and/or ASD and each of our symptom-based data-driven clusters. Second, our family-based design is optimal for whole genome sequencing and will allow us to determine whether patterns of inherited versus de novo mutations differentially cluster across the data-driven clusters [50, 253]. Again, we acknowledge the limited power of our sample for whole genome sequencing, and will join collaborative efforts (e.g. PGC,iPSYCH; Autism Speaks MSSNG Project; EU-Aims; Province of Ontario Neurodevelopmental Network [POND]).

### Parent phenotyping

Both biological parent’s complete self-report dimensional measures of ASD (Autism Quotient—Adults [AQ-A, [38]; SRS-2, [64], Adult Routines Inventory [ARI], [96]) and ADHD symptomatology (SWAN, [13], Conners' Adult ADHD Rating Scale, Conners et al. [61]; SDQ, [121]. Parent’s complete self-report measures of depression (Beck Depression Inventory [BDI], [24], anxiety (Beck Anxiety Inventory [BAI], [23]), and a quality of life measures (World Health Organisation Quality of Life Measure [WHOQOL-BREF], [285]) as parents of children with ASD and ADHD can experience poorer mental health and quality of life outcomes compared to parents with typically developing children [124, 167, 288].

### Database access

All raw data is stored on a central database with access only granted to current members of the research team who have personalised login details. Oculomotor and neuroimaging data are downloaded to local devices from the central database for cleaning, pre-processing, and analysis. Currently, access to the MAGNET Project’s data is only granted for members of the MAGNET research team and our collaborators from the EU-AIMS (LEAP) study [178]. Genotyping information will be made available to international research consortia, such as the PGC, where participant consent for sharing has been given. Upon completion of the project, the MAGNET Project data set will be changed to open access. Consent for sharing neuroimaging data will be in line with recommendations from the Open Brain Consent working group [208].

### Planned statistical analysis

A combination of supervised psychometric analyses and unsupervised clustering approaches will be used to converge on data-driven homogeneous ASD-ADHD clusters embedded within biologically-relevant dimensions based on previously derived factor score estimates [37, 106]. By using multiple measures of target constructs to create latent variable phenotypes, we can maximise our study’s statistical power and strengthen the representation of our key constructs [259]. The utility of dimensional approaches for improving statistical power in psychopathology research, particularly psychiatric genetics, is well established [160, 275, 258]. Similarly, the advantages of phenotypic precision in improving signal-to-noise ratio and thus statistical power for detecting relationships with external criterion variables, including genetic variation, has also been outlined [56, 259, 302]. Hierarchical models of psychopathology, such as HiTOP, which parse phenotypic variation into homogeneous components at varying levels of granularity and specificity are a particularly powerful approach to linking with genetics [239, 291]. Accounting for phenotypic heterogeneity through latent subtyping using hybrid models also has the potential to confer increased statistical power [105, 106]. Furthermore, combining measures of a construct across modalities removes confounding method variance [92, 219]. Obtaining information from multiple informants also controls for informant bias, whilst discrepancies between informant reports provides additional sources of information relevant to developmental psychopathology that can be the subject of further analysis [176, 176, 177, 177]. The MAGNET study leverages all of these approaches in combination, which not only improves power, but also increases consistency and efficiency and reduces bias in statistical estimation [238, 255]. Moreover, the MAGNET Project’s representative sample and measures are important prerequisites for robust clustering methods to avoid model overfitting and poor reproducibility [43, 223].

Dimension reduction strategies, such as exploratory factor analysis and exploratory structural equation modelling [15, 71, 182], or multidimensional item response theory [224], will be used on each participant’s raw scores to first identify their factor or scale score estimates representing their standing on these latent dimensions. Unbiased feature selection and optimising latent model fit in this step, prior to later clustering analyses, can reduce the interference of variance from extraneous noise. It is also acknowledged that there may be clustering and nesting within the data based on sampling (e.g. participants from the same family) and testing (e.g. testing sessions, assessors) procedures [57, 187]. Subsequent analyses will account for these effects, though the choice of correction method will depend on the characteristics of our final dataset.

Factor mixture modelling is one possible supervised clustering method that we will employ for our subtyping analyses. Factor mixture modelling can uncover homogeneous clusters within continuous and categorical data embedded within dimensional models of psychopathology by utilising probabilistic modelling techniques [37, 179, 189]. The flexibility of factor mixture modelling permits the testing and comparison of multiple models with varying numbers of a priori specified clusters. Alternatively, unsupervised machine learning techniques may be better suited for addressing specific research questions related to uncovering underlying structures in the data, and identifying clusters in a "bottom up" way. For example, HiTOP approaches to taxonomy make few assumptions regarding symptom-level data, and instead take the structure or shape of the subtypes from the data itself [133]. Community detection is one possible unsupervised approach, which combines graph theoretic analyses to detect homogeneous communities/clusters (i.e., highly connected sets of nodes). By ensuring that the algorithm achieves a connected graph, our analyses will parsimoniously account for all participants. These supervised, unsupervised, and hybrid approaches will help to empirically unify the theoretical grounding of MAGNET’s research questions with the power of cutting-edge data-driven analysis techniques to address heterogeneity. Moreover, these techniques are diagnosis-naïve, thus allowing MAGNET to fully embrace the transdiagnostic features of our biobehavioural subtypes. Finally, although MAGNET aims towards data-driven clusters using symptom and behavioural data, the potential utility of incorporating neurocognitive or genetic components in defining clusters will not be overlooked [58, 99].

## Discussion

The MAGNET Project has completed initial piloting of the study protocol and entered into an open recruitment phase. It is the first large-scale study using a family design to take a truly transdiagnostic approach to ASD and ADHD that aligns with the principles of the RDoC matrix and HiTOP model of psychopathology.

### Challenges in study design, recruitment, and data quality

#### Study design

A significant amount of time is required in the conceptualisation of an assessment battery that is appropriate for the large range of cognitive abilities and ages, while adequately capturing dimensional ASD and ADHD traits. As a number of the measures included in the protocol were not initially intended for use across broad age ranges or levels of cognitive ability, it is important to allow for extra time during piloting to determine the minimum age and cognitive level for tasks with novel applications. Although such an approach required more time initially, it will translate into a high-quality dataset upon project completion.

One of the measures used to confirm an ASD diagnosis, the ADOS-2, was chosen as it is internationally recognised as part of gold standard assessment. Uniquely, *all* children who participate in the MAGNET Project complete an ADOS-2. The ADOS-2 research training dictates coding of observed behaviours with no clinical interpretation to ensure research-reliable coding. Differentiating the social difficulties of children with ADHD on the ADOS-2 can be challenging, which has also been previously noted by Grzadzinski et al. [127]. ADOS data from children with ASD and ADHD also has the potential to improve clinical phenotyping across the ASD-ADHD spectra. Analysis of individual ADOS items may elucidate which items are more sensitive to ASD and which items are driven by ADHD presentations. Clinical cases are reviewed using all measures, including the ADOS-2 and the DAWBA, by the team’s paediatrician and psychologist to determine a best clinical estimate (see Additional file 2). The best clinical estimate process has been imperative in confirming diagnostic status.

#### Recruitment

The inclusion of children with ID will facilitate a sample that is largely representative of our target population, specifically within ASD. However, recruitment uptake for families of children with ID has been slow. These families often have children with high treatment needs which can be time consuming, in turn reducing the likelihood of these parents enrolling in a time-intensive research protocol. An alternative targeted recruitment strategy for these families will be needed moving forward, including direct communication with specialist school settings to engage teaching staff in the recruitment process for their learning community. Partnering with community grant funds and the Australian National Disability Insurance Scheme (NDIS) are further strategies the MAGNET team intends to utilise for recruitment of these children.

As ASD and ADHD are highly heritable, with evidence for shared genetic liability in families, this inherently limits the number of possible unaffected siblings. A large number of families with children with ASD and/or ADHD will therefore be required to achieve sufficient numbers of unaffected siblings for high powered statistical analysis.

#### Data quality

A number of the large-scale biobanking projects and multi-site studies can experience significant missingness in their data. Protecting against missing data has been a key priority in the MAGNET Project protocol development. Initial piloting highlighted that ensuring parents completed all online questionnaires before attending the in-person research visits reduced missing data, and increased attendance rates to research visits. Comprehensive data collection at the initial point of contact with families will also allow us to determine if attrition and resulting missingness is attributed to characteristics of the family or child, thus allowing us to model the missingness and avoid bias in our results. With the oversight from the project’s supervising psychologist, families are provided with a results summary after participating, including outcomes from cognitive assessments, language assessments (where applicable), ASD and ADHD symptom scales, and ADOS-2 ratings. To increase retention rates between the first and second research visit for each family, the neurocognitive tasks are completed in the first research visit and the cognitive assessment and ADOS-2 are completed in the second. Importantly, other measures used in the best clinical estimate review, such as the DAWBA, are completed prior to participants first research visit. Saliva collection from all members of the family pedigree has also been challenging, especially from fathers. Currently we have noted that mothers will primarily bring children to their research visits. Good follow-up and regular contact with the family is imperative in ensuring the least amount of missing genetic data. Minimal manual handling of data with automatic backups of all clinical, neurocognitive, and oculomotor data reduces the risk of missing data through technical or human error. Sophisticated analysis strategies to manage missingness will be utilised by the MAGNET Project that accommodates some missing data under assumptions of Missing Completely At Random, or Missing At Random, such as multiple imputation, auxiliary variables, and expectations-maximisation algorithm [93, 123].

The diversity of clinical specialists on the MAGNET Project team, including psychologists, cognitive neuroscientists, paediatricians, psychiatrists, and speech pathologists, is relatively unique. When research teams are large, this increases potential variability in administration of assessments, and thus variability in data quality. The MAGNET Project team undergo regular and ongoing staff training and clinical supervision from the project’s supervising psychologist. As a result, all members are consistently building skills to maximise participant engagement and data-capture across all tasks and assessments.

### Limitations

It is possible that the MAGNET Project’s sampling strategy will not achieve a true community sample upon completion. However, a variety of recruitment avenues and methods will be utilised to achieve a sample with breadth in symptomatology and phenotype. The project will provide insight into ASD and ADHD’s place within a hierarchical taxonomy of psychopathology and neurodevelopment. Although the study primarily targets traits central to these disorders, the full breadth of neurodevelopmental difficulties and common comorbidities (e.g. anxiety) are not captured with the same degree of granularity. The MAGNET Project will therefore provide one piece of the much larger puzzle in the quest for understanding neurodevelopment in a hierarchical framework. The broad range of cognitive abilities captured by the project, which allows a more representative sample, also means a proportion of children with more severe ID may not be able to complete all neurocognitive and/or imaging protocols. Nevertheless, our minimum dataset protocol is designed to provide minimal missing data across key tasks.

## Conclusion

Clinical heterogeneity and unitary conceptualisations of ASD and ADHD have hampered attempts to understand the structure of developmental psychopathology and associated neuropsychology, neurobiology and genetics. Current attempts to uncover the genetic aetiology of ASD/ADHD are limited with respect to one or more of the following: (1) recruitment is restricted to diagnostic categories that ignore the dimensional organisation of psychopathology symptoms, comorbidity, and within-group heterogeneity [163]; (2) minimal phenotyping in large samples; or (3) deep phenotyping in smaller samples [239]. Using deep phenotyping, dimension reduction techniques, factor mixture modelling, and machine learning techniques, the MAGNET Project aims to identify unique, homogeneous ASD-ADHD clusters of individuals with similar behavioural, neurocognitive, neuroimaging and, potentially, genetic profiles. The MAGNET Project will be one of the first studies to combine a dimensional conceptualisation of developmental psychopathology, in combination with deep phenotyping in a large sample to investigate the behavioural, neurocognitive, neuroimaging and genetic markers in ASD and ADHD. This study is well-positioned to uncover novel, homogeneous data-driven clusters with potential implications for ASD and ADHD diagnosis and treatment.

## Supplementary Information

**Additional file 1**. MAGNET Project study protocol summary.

**Additional file 2**. Best clinical estimate protocol.

**Additional file 3**. Protocol for testing non-verbal children and children with ID.

**Additional file 4**. Clinical assessments protocol.

**Additional file 5**. Research visit and neurocognitive testing protocol summary.

**Additional file 6**. Oculomotor testing protocol.

**Additional file 7**. Saliva collection protocol.

## Data Availability

Currently MAGNET Project data is stored on a central database with access currently granted to members of the research team and our collaborators from the EU-AIMS (LEAP) study [178]. Upon study completion database access will be opened to the scientific community.
