# Supplementary material for: The Monash Autism-ADHD genetics and neurodevelopment (MAGNET) project design and methodologies: a dimensional approach to understanding neurobiological and genetic aetiology
Source: Mol Autism. 2021 Aug 5;12:55. doi: 10.1186/s13229-021-00457-3 (PMC8340366; doi:10.1186/s13229-021-00457-3)
Supplement: Supplementary file 1 — Additional file 1. MAGNET Project study protocol summary. [file 13229_2021_457_MOESM1_ESM.docx]

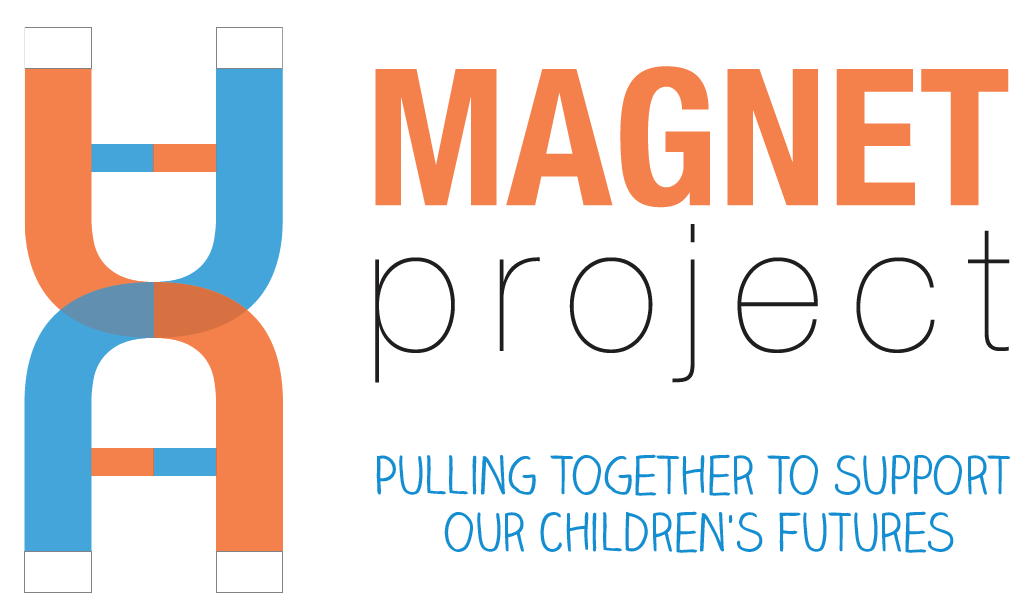


PROTOCOL

The Monash Autism/ADHD Genetics and Neurodevelopment (MAGNET) Project

**Principal investigators**

Dr Beth Johnson, Prof Mark Bellgrove

Turner Institute of Brain and Mental Health

Monash University

Date: 10 September 2020

Revision: 4

**Confidential**

This document is confidential. It may not be transmitted, reproduced, published, or used without prior written authorization.

**Statement of Compliance**

This document is a protocol for a research project. This study will comply with this protocol, the conditions of the ethics committee approval, and the NHMRC National Statement on ethical Conduct in Human Research (2018).

Contents

[Summary 8](#_Toc55489485)

[1 Background 8](#_Toc55489486)

[1.1 Summary 8](#_Toc55489487)

[1.2 Project aims 9](#_Toc55489488)

[1.2.1 Part 1: Assessing overlap of ADHD and ASD behaviours 10](#_Toc55489489)

[1.2.2 Part 2: Genetics of ADHD and ASD behaviours 10](#_Toc55489490)

[1.2.3 Part 3: Engagement with the ASD and ADHD community 10](#_Toc55489491)

[1.3 Affiliated studies 10](#_Toc55489492)

[1.4 Sample size and analysis 10](#_Toc55489493)

[1.5 Outcome measures 11](#_Toc55489494)

[2 General study management 11](#_Toc55489495)

[2.1 Staff training 11](#_Toc55489496)

[2.1.1 Working with Children Check 11](#_Toc55489497)

[2.1.2 Good Clinical Practice training 12](#_Toc55489498)

[2.1.3 Curriculum vitae 12](#_Toc55489499)

[2.2 Ethics 12](#_Toc55489500)

[2.2.1 Monash University 12](#_Toc55489501)

[2.2.2 Schools 12](#_Toc55489502)

[2.2.3 Monash Children’s Hospital 12](#_Toc55489503)

[2.3 Site management 12](#_Toc55489504)

[2.3.1 Monash University 12](#_Toc55489505)

[2.3.2 Schools 12](#_Toc55489506)

[2.4 Record keeping 12](#_Toc55489507)

[2.4.1 MAGNET participant registration 12](#_Toc55489508)

[2.4.2 Case report forms (CRFs) 12](#_Toc55489509)

[2.5 Data Security and Management 13](#_Toc55489510)

[2.5.1 Confidentiality 13](#_Toc55489511)

[2.5.2 Secure data storage 13](#_Toc55489512)

[2.5.3 Erasing or modifying data 13](#_Toc55489513)

[2.5.4 Cross checking data 13](#_Toc55489514)

[2.6 Communication with research participants 14](#_Toc55489515)

[2.6.1 General notes on communication 14](#_Toc55489516)

[2.6.2 Managing the study email account 14](#_Toc55489517)

[2.7 Adverse events 14](#_Toc55489518)

[2.7.1 Risk Assessment Procedure 15](#_Toc55489519)

[2.7.2 Infection Control 15](#_Toc55489520)

[3 Study protocol 16](#_Toc55489521)

[3.1 Protocol summary 16](#_Toc55489522)

[4 Participant recruitment & enrolment 17](#_Toc55489523)

[4.1 Recruitment through schools 17](#_Toc55489524)

[4.2 Recruitment from the community 17](#_Toc55489525)

[4.2.1 Recruitment through social media 17](#_Toc55489526)

[4.2.2 Recruitment from existing study databases 17](#_Toc55489527)

[4.3 Recruitment via referring clinicians 17](#_Toc55489528)

[5 Participant inclusion and exclusion criteria 17](#_Toc55489529)

[5.1 Assessing eligibility 18](#_Toc55489530)

[6 Informed consent 18](#_Toc55489531)

[6.1 Informed consent from the parent 18](#_Toc55489532)

[6.1.1 What & why: the research study 19](#_Toc55489533)

[6.1.2 What & why: the research procedures 19](#_Toc55489534)

[6.1.3 Risk to participants 19](#_Toc55489535)

[6.1.4 Benefits of participation 20](#_Toc55489536)

[6.1.5 Confidentiality 21](#_Toc55489537)

[6.1.6 The three limits to confidentiality 21](#_Toc55489538)

[6.1.7 Voluntary nature of participation 21](#_Toc55489539)

[6.1.8 Right to access information 21](#_Toc55489540)

[6.1.9 Complaints or concerns 22](#_Toc55489541)

[6.1.10 Question time 22](#_Toc55489542)

[6.1.11 Signing the consent form 22](#_Toc55489543)

[6.2 Informed consent from the child 22](#_Toc55489544)

[6.2.1 Ask the child about their understanding of the study 22](#_Toc55489545)

[6.2.2 Discuss voluntary participation and withdrawal 23](#_Toc55489547)

[6.2.3 Verbal and non-verbal cues to assess understanding 23](#_Toc55489548)

[6.3 Informed consent from the other parent 23](#_Toc55489549)

[6.4 Informed consent from the child’s teacher 23](#_Toc55489550)

[6.5 Storage of participant consent forms 23](#_Toc55489551)

[6.6 Withdrawal of consent 23](#_Toc55489552)

[6.7 Checklist for consent 23](#_Toc55489553)

[7 Methodology 24](#_Toc55489554)

[7.1 Assign each family and individual participant a unique code 24](#_Toc55489555)

[7.2 Participation requirements 24](#_Toc55489556)

[7.3 Pre-site visit 28](#_Toc55489557)

[7.3.1 Child and parent 1 questionnaire 28](#_Toc55489558)

[7.3.2 Sibling questionnaire 28](#_Toc55489559)

[7.3.3 Vineland Adaptive Behaviour Scale – Third Edition 28](#_Toc55489560)

[7.3.4 Parent 2 questionnaire 28](#_Toc55489561)

[7.4 Site Visit: standardised clinical assessments 28](#_Toc55489562)

[7.4.1 Wechsler Abbreviated Scales of Intelligence - Second Edition 29](#_Toc55489563)

[7.4.2 Wechsler Preschool and Primary Scale of Intelligence - Fourth Edition 29](#_Toc55489564)

[7.4.3 Wechsler Intelligence Scale for Children - Fifth Edition 29](#_Toc55489565)

[7.4.4 Wechsler Adult Intelligence Scale - Fourth Edition 29](#_Toc55489566)

[7.4.5 Vineland Adaptive Behaviour Scale – 3 30](#_Toc55489567)

[7.4.6 The Developmental, Dimensional and Diagnostic Interview (3di) 30](#_Toc55489568)

[7.4.7 Autism Diagnostic Observation Schedule – Second Edition 30](#_Toc55489569)

[7.4.8 Clinical Evaluation of Language Fundamentals – Fifth Edition Screening Test 30](#_Toc55489570)

[7.4.9 Clinical Evaluation of Language Fundamentals – Fifth Edition 30](#_Toc55489571)

[7.4.10 Clinical Evaluation of Language Fundamentals – Preschool Edition 31](#_Toc55489572)

[7.4.11 Preschool Language Scaled – Fifth Edition (PLS-5) 31](#_Toc55489573)

[7.4.12 Intra-site reliability 31](#_Toc55489574)

[7.4.13 Best estimate of clinical diagnosis 31](#_Toc55489575)

[7.5 Site visit: Neurocognitive Testing 31](#_Toc55489576)

[7.5.1 Go/No-Go 31](#_Toc55489577)

[7.5.2 Face Recognition 32](#_Toc55489578)

[7.5.3 Spatial Working Memory – Find the Phone 32](#_Toc55489579)

[7.5.4 Karolinska Directed Emotional Faces 33](#_Toc55489580)

[7.5.5 Reading the Mind in the Eyes Task – Child 33](#_Toc55489581)

[7.5.6 Continuous False Belief – Sandbox Task 34](#_Toc55489582)

[7.5.7 New Cambridge Gambling Task 35](#_Toc55489583)

[7.5.8 Stop Signal Task 35](#_Toc55489584)

[7.5.9 New Reversal Learning 35](#_Toc55489585)

[7.5.10 Spence Children’s Anxiety Scale 36](#_Toc55489586)

[7.5.11 Child’s Depression Inventory 2 (CDI-2) 36](#_Toc55489587)

[7.5.12 Beighton’s Hypermobility Scale 37](#_Toc55489588)

[7.5.13 Physical Measurements 37](#_Toc55489589)

[7.6 Site visit: Eyetracking 37](#_Toc55489590)

[7.6.1 Visually guided saccade 37](#_Toc55489591)

[7.6.2 Antisaccade paradigm 37](#_Toc55489592)

[7.6.3 Smooth pursuit 1: Smooth pursuit eye movement 37](#_Toc55489593)

[7.6.4 Smooth pursuit 2: Step ramp 37](#_Toc55489594)

[7.7 Salivary DNA Sample 37](#_Toc55489595)

[7.7.1 In person saliva collection 38](#_Toc55489596)

[7.7.2 Mail-out saliva kits 38](#_Toc55489597)

[8 Brain structure and function 38](#_Toc55489598)

[9 Clinical Report 38](#_Toc55489599)

[10 Analysis methods 39](#_Toc55489600)

[11 References 39](#_Toc55489601)

**Acronym list**

3Di Autism Diagnostic Interview-Revised

ABC Aberrant Behaviour Checklist

ADOS-2 Autism Diagnostic Observation Schedule – Second Edition

ADHD Attention deficit hyperactivity disorder

AE Adverse Event

AQ Autism Quotient

AQ-C Autism Quotient-Child Version

AR Adverse Reaction

ARI Adult Routines Inventory

APA American Psychological Association

ASD Autism Spectrum Disorder

BAI Beck Anxiety Inventory

BD Block design

BDI-2 Beck Depression Inventory-2

BMI Body mass index

CAARS Conners’ Adult ADHD Rating Scale

CBCL Child Behaviour Checklist

CCC-2 Children’s Communication Checklist 2

CDI Children’s Depression Inventory

CELF-5 Child Evaluation of Language Foundations – 5^th^ edition

CHIP-CE Child Health and Illness Profile –Child Edition

CPRS Conners Parent Rating Scale

CRF Case Report Form

CRI Child Routines Inventory

CSHQ Children’s Sleep Habits Questionnaire

CV Curriculum Vitae

DAWBA Development and Wellbeing Assessment

DSM Diagnostic and Statistical Manual of Mental Disorders

DSMB Data Safety Monitoring Board eCRF Electronic Case Report Form

EEG Electroencephalogram

EF Executive Function

EQ Empathy Quotient

ES Effect size

EU-AIMS Autism Research in Europe

FSIQ Full-Scale Intelligence Quotient

GCP Good Clinical Practices

HREC Human Research Ethics Committee

ID Intellectual Disability

IQ Intelligence Quotient

LEAP Longitudinal Europe Autism Project

MAGNET Monash Autism/ADHD Genetics and Neurodevelopment Project

MASSIVE Multi-modal Australian SciencesS Imaging and Visualisation Environment

ML Machine learning

MRI Magnetic Resonance Imaging

PC2 Physical Containment 2 Laboratory

PIQ Performance Intelligence Quotient

SCAS Spence Children’s Anxiety Scale

SRS-2 Social Responsiveness Scale 2^nd^ Edition

SDQ Strengths and Difficulties Questionnaire

STEM Curriculum based on science technology engineering and mathematics

SWAN Strengths and Weaknesses of ADHD Symptoms and Normal Behaviour scale

TMS Transcranial Magnetic Stimulation

VABS-3 Vineland Adaptive Behaviour Scale -3

VIQ Verbal Intelligence Quotient

WASI-II Wechsler Abbreviated Scales of Intelligence – II

WHOQOL World Health Organisation Quality of Life

WPPSI-IV Wechsler Preschool and Primary Scale of Intelligence - Fourth Edition

WISC-V Wechsler Intelligence Scale for Children - Fifth Edition

WAIS-IV Wechsler Adult Intelligence Scale - Fourth Edition

# Summary

| Full title | Understanding the behavioural and genetic overlap between autism spectrum disorders (ASD) and attention deficit hyperactivity disorder (ADHD) |
| --- | --- |
| Acronym | MAGNET Project: Monash ASD/ADHD Genetics and Neurodevelopment Project |
| Sponsor name | Monash University & Monash Children’s Hospital |
| Principal investigators | Dr Beth Johnson, Prof Mark Bellgrove |
| Research team members | This includes clinical/research staff and students who are involved in the project. All current research team members are included as part of ethics and are recorded in the delegation log. |
| Conditions being investigated | Autism spectrum disorder (ASD) and attention deficit hyperactivity disorder (ADHD) |
| Anticipated study duration | 5 years |
| Study design | Multi-dimensional phenotyping and genetic analysis of cases and controls, with supervised and unsupervised analysis methods, to identify clusters across the ASD-ADHD spectra |
| Project aims | Identify data-driven clusters across ASD-ADHD spectra using deep phenotyping of symptoms and behaviours  Investigate the degree of familiality for these data-driven symptom clusters dimensional phenotypes relevant to ASD-ADHD; and, relatedly,  Map the neurocognitive and brain imaging correlates of these ASD-ADHD clusters  Explore their genetic correlates |
| Sample size | 1200 children who are both typically developing, and those with elevated ASD, ADHD, or ASD-ADHD traits. |
| Endpoints | Identification of homogeneous clusters across the ADHD-ASD spectra, based on symptoms and behaviours, and the neurobiological (neurocognitive and brain imaging) correlates of these subtypes; identification of novel genetic markers associated with ASD and ADHD traits or clusters. |
| Eligibility criteria | **Controls:** Age 4-18 years, English speaking, no immediate family member with a diagnosis of ASD and/or ADHD, no uncorrected visual or hearing impairment.  **ASD/ADHD:** Age 4-18 years, English speaking, no uncorrected visual or hearing impairment, a diagnosis of ASD and/or ADHD, or currently under investigation for ASD and/or ADHD without known genetic (e.g. Fragile X) or environmental causes (e.g, fetal alcohol syndrome, traumatic brain injury). |
| Study location/s | Research appointments will be held at Monash University. |

# Background

## Summary

Attention deficit hyperactivity disorder (ADHD) and autism spectrum disorder (ASD) are the two most prevalent neurodevelopmental disorders, affecting approximately 5% and 2.5% of children, respectively (Polanczyk, Willcutt, Salum, Kieling, & Rohde, 2014; Randall et al., 2016). ADHD and ASD symptoms co-occur in 20-50% of cases. Children with co-occurring ADHD and ASD symptoms have poorer outcomes socially, emotionally, behaviourally, and academically (Thomas, Sciberras, Lycett, Papadopoulos, & Rinehart, 2015). While there are clear environmental and psychosocial influences, strong genetic contributions to the aetiology of both disorders are irrefutable (Grzadzinski et al., 2011; Hawi et al., 2015; Rommelse, Franke, Geurts, Hartman, & Buitelaar, 2010). Although genetic overlap between ADHD and ASD is implicated by the involvement of common genes in both disorders (Grzadzinski et al., 2011; Rommelse et al., 2010), gaining traction on this issue has been hampered by studies that have recruited samples of patients with either ADHD or ASD alone, and statistical approaches that have treated each as a unitary disorder without regard to their inherent clinical heterogeneity. Further to this, it remains entirely unclear whether children presenting with co-occurring ADHD/ASD symptoms have a distinct genetic profile to those with either disorder alone.

How ADHD and ASD symptoms appear can be very different among children, which can make it difficult for clinicians to predict the outcome for the child, response to treatment, and what treatments should be offered.

There are now major international efforts to recharacterize ADHD and ASD, and in particular, when the two conditions co-occur within the same individual. To achieve this, we need to study specific behaviours that exist across the general population, but in their most severe form present as ADHD or ASD. We can also use genetic, molecular, neurocognitive and structural (i.e. brain imaging) definitions to identify signatures that best define ADHD/ASD subtypes. We hope this approach will redefine diagnosis, improve diagnostic reliability and improve treatment outcomes.

This approach has been undertaken for other psychiatric disorders, such as schizophrenia and bipolar disorders (Clementz et al., 2016). Biologically informed models of symptom overlap across the ADHD/ASD spectra are necessary for improving diagnosis and informing differential treatment options. The MAGNET project will comprehensively phenotype core cognitive traits that capture the breadth of ADHD/ASD spectra and use data-driven methods to identify substructure in the data, reflecting homogeneous clusters, which can then be mapped to neurocognitive, brain imaging, and genetic traits.

We also are interested in how genetics may be involved in the symptoms we see across children with ADHD and ASD. We know that ADHD and ASD symptoms arise largely from genetic and biological causes. We will look at participant's DNA for those genes that we think influence behaviours like attention, memory and language.

By comprehensively phenotyping neurocognitive traits that capture the complexity of the ADHD/ASD spectra and its relationship to genetic risk, we can delve below DSM-5 symptom descriptions and answer questions about the clinical, genetic and neurobiological overlap between ADHD and ASD. This approach has the potential to identify clinically and functionally relevant clusters that could redefine our conceptualisation of ADHD and ASD. It also has the potential to assist clinicians in improving predictions about outcomes, and predictions about response to treatment.

## Project aims

We will establish a group of 1200 families of children aged between 4 and 18 years of age who are both typically developing children, and those with elevated ASD, ADHD, or ASD+ADHD symptoms to ensure both ends of the ASD-ADHD spectra are appropriately sampled. In addition, unaffected and affected siblings of probands will be recruited. We will use this data to:

1) identify data-driven clusters across ADHD-ASD spectra using deep phenotyping of symptoms and behaviours;

2) investigate the degree of familiality for these data-driven symptom clusters;

3) map the neurocognitive and brain imaging correlates of these data-driven symptom clusters; and

4) explore their genetic correlates.

### Part 1: Assessing overlap of ADHD and ASD behaviours

The aim of this part of the project is to comprehensively measure aspects of how we behave, which at their most extreme, are seen in ADHD and ASD: attention, cognitive control, working memory, repetitive behaviours, social processes, language & communication. By comprehensively assessing these domains across the whole ASD-ADHD spectrum of traits, from children who are typically developing through to those with a clinical diagnosis, we can begin to identify and more homogeneous clusters across this spectrum.

### Part 2: Genetics of ADHD and ASD behaviours

ADHD and ASD symptoms come largely from genetic and biological causes. A substantial amount of phenotypic variation can be attributed to aggregations of single nucleotide polymorphisms (SNPs), and some variants of these SNPs have been linked to risk for ADHD or ASD, or associated with ASD or ADHD symptoms. Utilizing parent-offspring (mother/father/children), even with just a single parent (mother OR father/child or children) is a powerful and robust method for assessing gene variants in disease, and how they are passed from parent to child, which avoids the problem of population stratification that may confound conventional case/control studies.

Polygenic risk scores are another way of indexing genetic vulnerability for psychiatric disorders. The more aggregations of risk variants an individual carries, the greater their polygenic risk. Participants will provide a small saliva sample, used to extract DNA. These methods will be used to link behavioural subtypes to genetic risk.

### Part 3: Engagement with the ASD and ADHD community

This project has considerable engagement with clinics, schools and the general community, and we are therefore uniquely placed to include community engagement and education as part of the project. Parents and teachers will be offering considerable time to participate in the project, and we receive funding from the NHMRC to conduct our research, therefore it is imperative that we give back to the community.

The two areas of focus for community engagement are: 1) education about neurodevelopmental disorders within the community (e.g. through online platforms, parent forums in schools, engagement and presentations to clinicians), and 2) conveying the importance of STEM more broadly (e.g. through social media, engagement with the CSIRO Scientists in Schools program). This is built into the project by way of offering presentations to schools and communities, to help educate parents and students taking part in the project.

## Affiliated studies

The MAGNET Project is an extension of the EU-AIMS Longitudinal European Autism Project (LEAP):

[**https://www.autismresearchcentre.com/project_32_leap**](https://www.autismresearchcentre.com/project_32_leap)

The design and methodologies, and clinical characterization of the EU-AIMS LEAP sample are available here:

[**https://molecularautism.biomedcentral.com/articles/10.1186/s13229-017-0146-8**](https://molecularautism.biomedcentral.com/articles/10.1186/s13229-017-0146-8)

[**https://molecularautism.biomedcentral.com/articles/10.1186/s13229-017-0145-9**](https://molecularautism.biomedcentral.com/articles/10.1186/s13229-017-0145-9)

The aims and methodology of the LEAP and MAGNET studies are aligned.

## Sample size and analysis

Within a multidimensional design, estimating sample size using power calculations is problematic because the number of variables shifts with the increasing granularity of the data, and statistical power changes as the first time point of data becomes available. For ML, a sample that instead captures a wide range of clinical and neurocognitive manifestations across the ADHD-ASD spectra is of greater importance (i.e. probands, siblings, controls). Consistent with other multi-dimensional studies employing similar analysis methods (B-SNIP3, EU-AIMS LEAP19) we will therefore recruit 1200 families of children who are both typically developing, and those with elevated ASD, ADHD, or ASD+ADHD symptoms, in addition to unaffected and affected siblings of probands.

A combination of supervised psychometric analyses and unsupervised clustering approaches will be used to converge on data-driven homogenous ASD-ADHD clusters embedded within biologically-relevant dimensions (Borsboom, Rhemtulla, Cramer, Maas, & Scheffer, 2016; Feczko et al., 2019). Dimension reduction techniques, such as exploratory factor analysis or multidimensional item response theory (Reckase, 2009) will be used to identify factor score estimates on latent variables. Factor mixture modelling is a hybrid approach that combines categorical and continuous latent variables and is ideal for uncovering clinically relevant clusters embedded within dimensional models of psychopathology (Clark et al., 2013; Miettunen, Nordström, Kaakinen, & Ahmed, 2016). Alternatively, where unsupervised machine learning techniques may be better suited for addressing specific research questions, community detection is one possible approach. Both techniques are diagnosis-naïve, allowing MAGNET to fully embrace the transdiagnostic features of our biobehavioural clusters.

## Outcome measures

The battery used in this protocol will characterize domains that capture the core traits associated with ASD and ADHD: internalising and externalising symptoms, attention and cognitive control, arousal, reward, working memory, perception, language, social processes, repetitive behaviours and sensorimotor processes. Each of the neurocognitive and clinical instruments will provide individual outcome measures (see Section 7.4 and Section 7.5) and a comprehensive profile of each participant’s strengths and weaknesses across cognitive and behavioural domains.

# General study management

## Staff training

Dr Beth Johnson is the lead investigator and main contact person for this project, under the supervision of Professor Bellgrove who heads the Bellgrove Laboratory at Monash University. Dr Beth Johnson has received training from the Donders Institute, Netherlands, and King’s College London, UK, for all aspects of the study protocol that overlap with EU-AIMS.

Dr Beth Johnson will oversee the training of research team members involved in data collection, including recruitment and consent, and administering the protocol.

New research team members will not commence their involvement in the project until they have been added as an investigator to the ethics, provided evidence of their Working with Children Check, and completed the appropriate training for their role.

All research team members will be listed on a study delegation log, which is maintained in the project’s REDCap database (Harris et al., 2019, 2009). The delegation log will specify which tasks each individual is approved to complete.

The project will have additional research management and administration support from trained research team members, including clinical/research staff and students. Supervision, led by Dr Beth Johnson or delegated as appropriate to the team’s supervising psychologist, will be provided fortnightly at a minimum, and involve any additional training required and/or clinical case study discussion.

### Working with Children Check

All research team members must have a current and valid Working with Children’s Check before commencing on the project.

### Good Clinical Practice training

All research team members must undertake Good Clinical Practice (GCP) training.

### Curriculum vitae

A copy of all research team members’ curriculum vitaes must be stored with the study files.

## Ethics

### Monash University

Monash University: CF16/1537 - 2016000806 – Monash ASD/ADHD Genetics and Neurodevelopment (MAGNET) study

### Schools

Department of Education: 2017_003570 - Understanding the behavioural and genetic overlap between autism spectrum disorders (ASD) and attention deficit hyperactivity disorder.

### Monash Children’s Hospital

Monash Health: RES-19-0000-372A - Understanding the overlap between autism spectrum disorders (ASD) and attention deficit hyperactivity disorder (ADHD)

## Site management

### Monash University

Research appointments will be held at Monash University. All saliva samples and DNA will be stored at Monash University. Staff must complete Biosafety training and facility induction to gain access.

Staff must complete any necessary inductions for research facilities before being given access to the facilities or booking systems.

### Schools

The research team may visit primary schools to conduct assessments in lieu of a research visit to Monash University. Participants will be recruited from schools from the public education system. Dr Beth Johnson will contact the principal of each school by email or phone and explain the project to them and gain permission for testing. Parents will be invited to complete screening, eligibility, and consent forms online, and paper copies will be provided at their request. Once consent is complete, parents will then be sent the Pre-Site Visit Questionnaire and be informed of the testing date at their child’s school.

## Record keeping

### MAGNET participant registration

A secure database (REDCap) will be used for all sensitive participant information, including personal details and identifiable data. All participants will be registered, this includes participants who were screened and ineligible and those who declined to continue with the project.

### Case report forms (CRFs)

During the testing sessions, a researcher will fill in a CRF for each participant. The CRF will act as a checklist for the completion of standardised assessments, neurocognitive tasks and saliva collection, where the date, time, and any comments are recorded. At the conclusion of the testing session, information recorded in the CFR will be entered into REDCap by a research team member.

Where applicable, research team members can enter additional information (e.g. relevant discussions with parents, or lengthy observations/clinical impressions) directly into REDCap.

## Data Security and Management

### Confidentiality

Participant confidentiality is strictly held in trust by the MAGNET Project investigators. This confidentiality is extended to cover clinical information relating to participants. The study documentation, data and all other information generated will be held in strict confidence. No information concerning the study or the data will be released to any unauthorized third party, without prior written approval of the institution. All records will only be identified by a unique participant identification number to maintain participant confidentiality. Identifiable information will not be released without written permission of the participant, except as necessary for monitoring by HREC or regulatory agencies

### Secure data storage

A unique participant identification number will be used to identify participants in all reporting, CRFs, electronic and hardcopy questionnaires and assessments. Only the research team will have access to and be able to re-identify data. Information will be kept for a minimum of seven years after the 18th birthday of participants, or for 15 years after the finish of the study, whichever date is later. Aggregated data only will be reported in publications and presentations, with individual identifying information removed. The findings of this study will be published in peer reviewed journals.

Hardcopy consent forms, questionnaires, CRFs, and standardised assessments will be stored in locked filing cabinets at Monash University.

Parents and teachers will more often complete questionnaires online via REDCap data capture tool. Data from REDCap will be periodically exported and backed up on a regular basis: this will minimise the risk of losing data should the data need to be recovered due to a software or hardware issue. All back-up data will be securely stored.

All data will also be stored across project databases, S drive (Monash University’s secure data storage) and REDCap. Psytools data is stored on a secure external server (Delosis) and will be periodically backed up onto S drive. Data may be transferred to the Multi-modal Australian ScienceS Imaging and Visualisation Environment (MASSIVE), a specialised high-performance computing facility at Monash University, for data analysis.

### Erasing or modifying data

When collecting data, research team members must not erase/delete any entry on a data collection form, or in REDCap. If a mistake has been made on a paper CRF, a line should be placed through the original entry so that it remains visible. The corrected value should be written in an adjacent space and a comment provided as to why the correction was made. Any corrections and comments should be initialled and dated by the research team member.

### Cross checking data

Research team members are required to cross-check all data collection forms for completeness, within one week following the testing session. Research team members must initial and date the front page of every form to indicate cross-checking has been completed. There is also a second opportunity to correct any missing data when clinical reports are created for families.

Wherever possible, missing data should be followed up immediately and added to the form where available. If data cannot be obtained, this should be noted on the form along with the reason.

To maintain data quality, a process of data checking and cleaning will be undertaken by the research team. We will keep a register of data issues to ensure problems with data are addressed in a timely manner and conduct regular audits of data management processes.

## Communication with research participants

### General notes on communication

When communicating with participants, it is important to use our professional channels (e.g. Monash email, Monash phone number, or blocked phone number). This is to ensure that participants cannot contact research team members out of hours, or privately. Occasionally in studies researching the brain and mental illness, participants can see research team members as someone with knowledge in this area and may contact them out of hours with non-study related issues (e.g. in a time of crisis). This puts the research team member and the participant in an unhelpful and potentially unsafe situation. As such, the following communication principles should be followed:

- Communicate with the participant using a Monash email address, Monash phone line, or a blocked phone number
- Advertise using official Monash contacts on posters and in emails.
- If advertising on websites or social media, use the study email address or MAGNET Project Facebook page.
- For text messaging (e.g., appointment reminders), there are online SMS services available, which send messages from an anonymous number.

All contact must be recorded in the relevant participant file in REDCap.

### Managing the study email account

The study email account will be listed on study documents and will act as the primary point of contact for parents, teachers, clinicians and any potential participants or other interested parties. Delegated research team members will have access to the study email address inbox.

An electronic tagging and filing system will ensure all emails are responded to by an appropriate research team member.

Do not delete any correspondence from participants, clinicians or staff from the account.

## Adverse events

In the unlikely event that any participants become distressed throughout the course of the study, the following procedure will be put in place. The member of the research team in direct contact with the participant will attempt to calm the participant and direct them toward appropriate resources, dependent upon the cause of the distress.

The event will be reported to the team’s supervising psychologist, who will then follow up with the participant to debrief them and ensure that they are no longer experiencing distress, to provide further comfort/brief counselling if needed, or referral to appropriate external services. The procedure for notifying and recording risk of harm for participants, including appropriate authorities and phone numbers, are provided at the end of the CRF. An Adverse Event Register to record and track any adverse events will be maintained in REDCap.

### Risk Assessment Procedure

All staff members and students will undergo risk assessment training, overseen by the MAGNET project’s supervising psychologist.

If risk is identified, document this as an adverse event in the Adverse Event Register on REDCap, along with the date and status of the adverse event (e.g., ongoing/resolved) and a completed risk assessment. The supervising psychologist is responsible for management and follow up with staff and participants.

### Infection Control

Research procedures will follow all government guidelines and any additional University or research facility directives, in order to minimize risks to research participants and team members. This includes following Monash University’s centrally managed risk framework for return-to-campus ‘Minimum requirements for a return to campus activities during the COVID-19 pandemic’ and ‘Risk Assessment Template for assessing work with people with physical distances of less than 1.5 metres’.

Our primary measures for infection control focus on:

- - Screening (all family members attending research session)
  - Hygiene (for participant families and research team)
  - Regular cleaning (before, after, and throughout the session)
  - Contact tracing (confirming contact details on arrival)

**Screening**

Prior to the research visit, we will inform parents of the following infection control procedures:

- We will contact families within the week prior to their upcoming research visit to complete a COVID-19 screening checklist. If families do not pass screening, we will reschedule the appointment and provide them with a list of COVID-19 testing sites.
- Only participating children and one parent/guardian can attend research visits at our testing facilities.
- The research visit will be discontinued and rescheduled if any family member reports, or is observed to have, any of the following symptoms; fever, cough, sore throat, shortness of breath, chills/body aches, headache, runny nose, muscle pain or diarrhoea.

**Hygiene**

During the research visit, all research team members, participants and parents will be required to maintain the recommended social distance to minimise the spread of infection (1.5 m between non-cohabiting people). Parents and children are to remain in the designated rooms/areas throughout the visit.

Research team members will request that participants wash their hands and use hand sanitizer at regular intervals.

To ensure young children are aware of these requirements, the research team member will give all children instructions in easy to understand language.

**Regular cleaning**

Before and after each family’s research visit, all testing equipment and surfaces will be disinfected. For items where this is not possible, the item will be given to the participant to take home (e.g. pencil used by child for writing) or placed in a secure container with clearly written instructions not to be open until the date at which surface degradation of potential infectious material is completed (at least 7 days based on current health advice).

**Contact tracing**

At the beginning of the research visit, the research team will confirm contact details with the parents and ensure they will be immediately contactable at those details for a minimum of 2 weeks following completion of the research visits. If there is a reported COVID-19 infection at the research facility, the primary family contact will be informed and provided with a list of COVID-19 testing sites.

# Study protocol

## Protocol summary

The aim of this project is to comprehensively measure specific behavioural and neurocognitive traits which, at their most extreme, are clinically impaired in cases of ADHD and ASD: internalising and externalising symptoms, attention and cognitive control, arousal, reward, working memory, perception, language, social processes, repetitive behaviours and sensorimotor processes.

We will establish a group of 1200 children who are both typically developing, and those with elevated ASD, ADHD, or ASD-ADHD traits. We are also inviting affected and unaffected siblings to take part in the study. A summary of the protocol is seen in Figure 1.


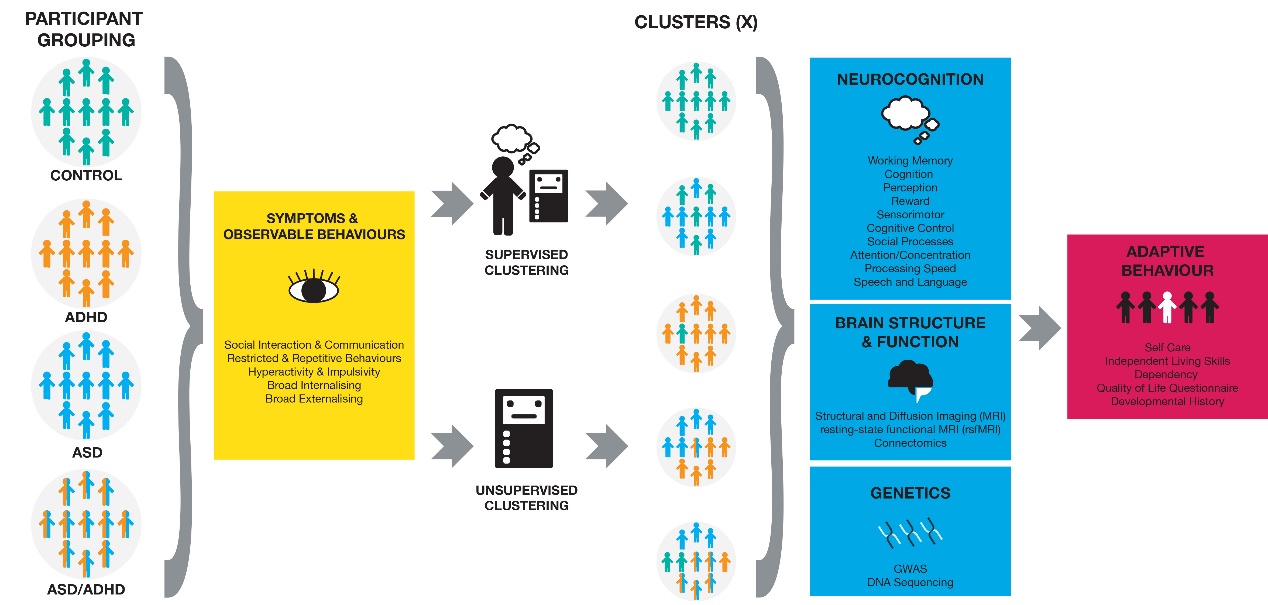


*Figure 1.* Protocol summary.

# Participant recruitment & enrolment

Participants will be recruited via multiple avenues, including schools (with Principals as first contact), online advertising and social media, referring clinicians, support groups, community centres, or, existing volunteer databases of the research team.

## Recruitment through schools

We will approach public schools regarding participation in our study. Dr Beth Johnson will contact school principals to explain the project. If a school is willing to participate, we will obtain written permission from the principal. Information packages will be sent for the school to distribute using their preferred platform (e.g. electronic correspondence, paper based etc.). This information will include a link to the online study summary, eligibility questionnaire, explanatory statement and consent form. For participants recruited through their school, the research appointment will be undertaken at school (unless additional assessments are required).

## Recruitment from the community

Participants will be recruited from the general population via advertisements on social media, radio, and word-of-mouth. Participants will be directed to the survey link for access to the online study summary, eligibility questionnaire, explanatory statement and consent form. Participants recruited from the community will be invited to attend research appointments at Monash University.

### Recruitment through social media

The MAGNET project has a Facebook page and an Instagram page.

**[https://www.facebook.com/magnetproject/](https://www.facebook.com/magnetproject/ )**

[**https://www.instagram.com/monashmagnetproject/**](https://www.instagram.com/monashmagnetproject/)

Participants can contact the research team via direct messaging, or by following the survey link to eligibility, explanatory statement and consent forms. Occasionally the research team use social media platforms to run promotions to recruit families across Melbourne. Dr Beth Johnson and designated research team members maintain the Facebook and Instagram pages and provide periodic updates to followers.

### Recruitment from existing study databases

Where Dr Beth Johnson or Prof Mark Bellgrove are co-investigators on other studies with overlapping study participant eligibility criteria, and where participants have given consent to be contacted for future research, Dr Beth Johnson will contact those participants and invite them to take part in the MAGNET project.

## Recruitment via referring clinicians

Private and hospital-based paediatricians or psychologists may provide information about the MAGNET Project to their clients. Families who request further information about the study will be contacted by our research team via email, phone or SMS. The research team will address any specific questions and direct them to the online study summary, eligibility questionnaire, explanatory statement and consent forms. The researcher may be invited to attend the clinic to provide information in person.

# Participant inclusion and exclusion criteria

Families will be excluded from this study if;

- The child has, or has had, a major illness or disability (e.g. history of neurological illness, including head injury or epilepsy).
- The child has uncorrected visual or hearing impairment
- The family (specifically the parents) are non-English speaking. Parents complete a significant number of questionnaires outside a research visit, and English is required in order to be able to complete these.
- The family has withdrawn from the study

Further, only Caucasian individuals will be invited to participate in the genetics section of this project. This exclusion criteria arises directly from the nature of the genetic work. Specifically, it is well established that single nucleotide polymorphisms have different allele frequencies in different ethnic groups. Differences in these frequencies prevents combining data across different ethnic populations, a problem referred to as population stratification. If data from different ethnic populations were combined, it would invalidate the genetic analyses and make publication of the data difficult. This criterion will be clearly defined in all promotional/advertising material and the scientific requirement to recruit only Caucasian individuals for the genetics section of the project will be made clear.

## Assessing eligibility

Families interested in taking part in the MAGNET Project will be provided with a link to an online study summary, eligibility questionnaire, explanatory statement and consent form. The explanatory statement provides additional information about the project and what is involved in participating:

**<https://redcap.cdms.org.au/surveys/?s=J7EFRRH4PR>**

Should a prospective participant not meet eligibility criteria, they will be sensitively told that the study is not a good fit for them, but that if of interest, the research team is happy to add their contact information to a volunteer database so that they may be contacted for future studies that are better suited.

# Informed consent

Once eligibility is ascertained, consent can be obtained either online (prior to the study visit) or on paper-based forms (during the study visit). Parents completing the online registration form will read the participant explanatory statement and consent form prior to providing consent. Informed consent will be obtained in writing (in hard copy or online) from parents on behalf of themselves and their participating child. The explanatory statement includes the contact details of the research team so that if parents have any questions or concerns about the project they can discuss these with a member of the research team.

Though written consent will be obtained online in most cases, a researcher should still contact the family **either via phone or email**, so as to provide as much information about the study as possible. It is important to ensure that the family is well informed and fully understands what is expected from their participation, and that they are happy to proceed with the project. Multiple opportunities should be offered for the family to consent and re-consent. It is also imperative that the child understands why they are participating.

Trained research team members are primarily responsible for ensuring written consent is obtained from all participating families prior to taking part in the study.

## Informed consent from the parent

In research, informed consent is a person’s agreement to take part in the study and its research activities (e.g. questionnaires, interview, cognitive tasks, EEG, MRI, TMS) when the decision to participate is made with a full understating of the purpose of the study, the requirements of participation, potential risks, potential benefits, how information will be handled, and any consequences of choosing to not participate.

The informed consent process involves a participant explanatory statement and consent form and a verbal or face-to-face discussion between the researcher and participant. Participants are provided with the participant explanatory statement and consent form prior to attending their first research session (e.g. provided as an email attachment, or link). A member of the research team will review the details of the study, guided by the explanatory statement, with the participant via phone prior to booking the first research session, and then again as the initial stage of the first research session.

The explanatory statement guides the discussion, but it is the discussion, and not the form, that is the true process of obtaining consent. The process of obtaining informed consent includes a discussion, in lay English terms, of each section of the explanatory statement and consent form, including (but not limited to) the following points:

### What & why: the research study

- What is the research question that the study is addressing?
- Why is the research question important? What broader/longer-term implications might the study findings have?

### What & why: the research procedures

- What research procedures will participants be required to engage in?
- What do each of these procedures involve?
- Why are these procedures part of the study (i.e. how do they answer the research question)?

Discussing the ‘what’s and why’s’ of the study is an excellent opportunity to build rapport with participants, and to maximise their engagement with the research procedures. It is helpful to emphasise that researchers can only answer research questions with the assistance of participants, and they should communicate gratitude for participants in helping to answer these questions. In doing so, researchers will help participants feel that they are part of the research team, valued, and contributing to science. This will maximise the likelihood of them having a positive experience, and the researcher collecting high quality data.

### Risk to participants

- What are the potential risks/inconveniences associated with the research activities?
- What is the likelihood of these risks occurring?
- If these risks were to eventuate, or a medical abnormality was detected (e.g. MRI), what are the potential consequences? It is important to give a full and balanced assessment of potential risks, and not to underplay risk or create unwarranted anxiety regarding the risk.
- Risk assessment: In some cases, participants will be invited to join the study via clinical or educational avenues. At all stages of the process it will be explained to participants that their participation is completely voluntary, is not related to their medical treatment/education, and will not affect their teacher/clinician relationship. Consent will be obtained by research team members based at Monash University (MAGNET Project team) who have no teacher/clinician relationship with participants. Participants will also be advised that they can withdraw from the study without affecting their relationship with their school/clinicians.
- **Identification of behavioural concerns (potential psychological risk to participant):** the study involves a range of standardised cognitive assessments and the evaluation of ASD and ADHD traits. As such, there is a chance that a child may be identified as having behavioural concerns of which the parents may not have previously been aware, which may cause distress to the participant. As part of the consenting process, we will ask parent(s)/caregiver(s) whether they have any concerns about their child’s development, and whether they would like to know if any developmental problems are identified. We also require that clinician details be captured at the time of enrolment, to ensure appropriate referral pathways can be followed, if necessary.

- **Distress/discomfort (potential psychological risk to participant):** Depression and anxiety measures are administered as part of the testing protocol, which may be uncomfortable or confronting for some participants. Situations in which participants become discomforted/distressed will be managed in accordance with Section 2.7 of this Protocol.
- **Genetic testing paternity results (potential psychological risk to participant):** As a result of the family-based design of the project, wherein we collect DNA from biological mother, father and child, we have the ability to determine paternity from the genetic analyses.  As a research laboratory, without the associated quality assurance and national accreditation for paternity testing, we are not placed to disclose, or provide feedback, on this type of sensitive information to participants. As such, our explanatory statement clearly states that no information regarding parent-child relatedness will be provided. i.e. “Although we will be analysing genetics in this study, information about parent-child relatedness will not be available.”
- **Time burden (potential psychological risk to participant):** The time commitment to families may be burdensome to many families, depending on the assessments that need to be administered to the child (e.g. as additional standardised clinical assessments may need to be administered if the child shows indicators for language difficulties). The research visit for the child may take between 4-9 hours, although this burden is managed through full informed consent, and allowing the time to be completed over multiple visits. The parent is required to complete psychological inventories/questionnaires about them self and their child which can also take between 3-4 hours, although this is managed through informed consent and can be completed at home and does not have to be completed all at once.
- **Working with vulnerable participants (potential psychological risk to participant):** The research will involve young children (aged 4-18 years) who may or may not have developmental delays, therefore the ability of children to understand relevant information and take part in limited discussion about participation in the research is going to be highly variable and require some degree of flexibility. Trained clinical/research staff and students will be directly involved with recruitment of clinical families and participants, and will determine whether these children have the maturity to consent to participation. Prior to the interview, the young participant will be reminded that it is voluntary to undertake the testing sessions and they can withdraw at any time or decline to answer specific questions.  In the event that the research team member feels that any participant is at risk, in distress, or is being deliberately uncooperative, the assessment will cease.

- **Infection control (potential/psychological risk to participant):** Research requiring face-to-face contact with participants places the research team member and the participant at risk of exposure to infectious diseases, e.g. COVID-19. Research procedures will follow all government guidelines and any University or research facility directives in order to minimize this risk. The primary infection control measures encompass participant education, participant screening, hygiene practices and routine cleaning and disinfection. Further infection control procedures are contained in section 2.7.2.

### Benefits of participation

- What are the specific benefits of participation (e.g. reimbursement, if intervention trial - potential for reduction in symptom severity)?
- What are the broad benefits of participation (e.g. opportunity to contribute to knowledge, to help answer research questions that may lead to new treatments, etc)? This is another excellent opportunity to help the participant feel like they are helping answer an interesting/important research question, and maximise the likelihood of their having a positive experience.

While this research is unlikely to directly benefit the participants involved, it should advance our knowledge and understanding of ASD and ADHD. This project has the potential to be a landmark study of international relevance. By comprehensively phenotyping neurocognitive traits that capture the complexity of the ASD/ADHD spectra and its relationship to genetic risk, we can delve below DSM-5 symptom descriptions and answer questions about the clinical, genetic and neurobiological overlap between ASD and ADHD. For example, do the inattentive symptoms observed in some children with ASD arise from a common or fundamentally distinct aetiology to that of ADHD? This project therefore offers the very real prospect of identifying clinically and functionally relevant clusters that could redefine our conceptualisation of ASD and ADHD. Such an approach has the potential to determine the most sensitive diagnostic tools, improve diagnostic reliability and reduce the time taken to diagnose. It also has the potential to assist clinicians in improving predictions about outcomes, and predictions about response to treatment. As discussed in Section 6.1.2, letting participants know that they are integral to the research provides an opportunity for them to feel that they are helping to answer an interesting/important question, and also maximises the likelihood that they have a positive experience.

### Confidentiality

This is an important principle in all research studies. As with all data collection, ensure that data collection is undertaken in a private space, and the following are clearly conveyed:

- Information collected during the study will remain confidential.
- Only the research team will have access to identifying information about participants.
- Each participant will be assigned a unique identification number, which will be used for hard copy, electronic, and biological data identification, rather than their name.
- Information linking participant names with their identification number will be securely stored and only accessible by members of the research team.
- All hard copy documents containing identifying information will be stored in a locked filing cabinet that only the research team have access to.
- Data used in publications and presentations will be deidentified.

### The three limits to confidentiality

1. The researcher is concerned that the participant is an acute risk of harm to themselves.

2. The researcher is concerned that the participant is an acute risk of harm to someone else.

3. The research data is subpoenaed by a court.

For clinical cohort participants, also convey that if one of the first two limits were triggered, the research team member would discuss the reasons for breaking confidentiality with the participant, the steps that will come next because of this, and that their senior research team member is the person that will need to be initially consulted about the next steps. The third scenario is extremely rare, but technically possible.

It is never appropriate to discuss information collected during the study (e.g. participants’ mental health history, medical history, drug-taking) with staff or students who are not part of the research team.

### Voluntary nature of participation

- Convey that participation in the study is entirely voluntary, and that participants are free to withdraw at any time, and that withdrawing will not impact their relationship with the research team, school or clinician.

### Right to access information

- Convey that participants have a right to access their data. At the conclusion of their participation in the study, they will be provided with a report which summarises the clinically relevant information that was gathered.
- Convey the limits to data access for the study in question, e.g. participants will not be provided with results from the neurocognitive tasks, as this is not clinically informative.

### Complaints or concerns

- Let the participant know that if they have complaints or concerns regarding the way the study is being conducted, or their participation in it, they can contact the Monash University Human Research Ethics Committee or Monash Health Human Research Ethics Committee who will investigate on their behalf. Indicate where in the explanatory statement the appropriate contact details are.

### Question time

- Encourage participants to ask questions throughout the informed consent discussion.
- At the end of the discussion, ask again if the participant has any questions.
- Answer all questions in lay English, avoiding high-level technical terms and jargon. If you do not know the answer to a question, check with a senior research team member and then convey the answer to the participant within an appropriate time frame.

### Signing the consent form

The final aspect of the informed consent procedure is confirming the participant has freely given their informed consent to take part in the study and correctly completed and signed the consent form. A copy is provided to the participant, either electronically or as a hard copy. As part of the informed consent process, parents will be asked to provide optional consent to allow us to approach the child’s other parent and teacher to collect data.

## Informed consent from the child

While it is the parent that provides consent for their child to participate, parents will be asked to discuss the study with their child as part of the parental consent process.

The research will involve young children (aged 4-18 years) who may or may not have developmental delays, therefore the ability of children to understand relevant information and take part in limited discussion about participation in the research is going to be highly variable and require some degree of flexibility.

Parents will be asked about their child’s capacity to understand what their participation in the research involves and gauge how their child might feel about participating, specifically that:

- The child will need to provide a small saliva sample by spitting into a tube
- We might ask their teacher to give information about the child
- That the information we collect will be private and won’t be shared with anyone
- The child can decline to participate without getting into trouble

This constitutes an opportunity to confirm consent. It will also be explained to the child that they can withdraw their consent to participate at any time.

### Ask the child about their understanding of the study

To determine if the young person has sufficient understanding of the study, the researcher will ask the young person two questions for example:

- Can you tell me two things we are going to ask you to do today?
- We are also going to ask some other people to tell us about how you have been going at school and at home. Can you remember who else we are going to speak with?

### Discuss voluntary participation and withdrawal

The research team member will remind the young person that participation is voluntary, that they can choose not to answer specific questions and can withdraw from the study at any time. The researcher team member will gauge competence in understanding by asking the child to summarize what has been explained to them, and assess both verbal (as outlined below) and nonverbal cues that the information has been understood.

### Verbal and non-verbal cues to assess understanding

Common nonverbal cues that may indicate lack of understanding in typically developing children, such as fidgeting, walking away or gaze aversion, are classic symptoms of many children along the ASD and ADHD spectrum, therefore some degree of flexibility in our criteria for assessing understanding is necessary. However, the research team are highly experienced with children with complex needs along the ASD and ADHD spectrum, and will cooperatively work with parents and use their professional judgement to determine competence and willingness to take part, while ensuring that children and their families do not feel coerced to take part.

## Informed consent from the other parent

If a second parent is available and is willing to participate in the study, they must also fill in a consent form. This is included as the first section in the Parent 2 Questionnaire on REDCap. A paper copy can be supplied, if requested.

## Informed consent from the child’s teacher

Where parents have consented for the child’s teacher to be contacted, the research team will contact the school informing them of the study, and that we are requesting that a member of their staff participates. The school will be asked to provide the research team with the teacher’s email address. The Principal or school will not be required to take any further action.

A research team member will then send the teacher an email that explains the study and why we are contacting them, and a link to the Teacher Questionnaire on REDCap. Consent is given by the teacher before commencing the survey.

We will not be informing teachers of their students' diagnoses, however when we are reporting results to teachers, we will explain the purpose of the study.

## Storage of participant consent forms

Consent is documented electronically in REDCap (most commonly) or in hard copy. All participant hard copy consent forms will be stored in a locked filing cabinet. Only members of the research team have access to these files. Hard copy consent forms are scanned and uploaded to the participant’s record in REDCap. Parents are provided with an emailed copy of the electronic consent, or a photocopy of the completed hard copy consent form for their records.

## Withdrawal of consent

Participants who withdraw from the study, or are lost to follow up, will not be replaced. A note of their withdrawal will be made on REDCap and all data relating to that participant will be destroyed, at their request.

If a teacher withdraws, or the child changes school, the parent will be asked if they wish to nominate a new teacher who has regular contact with the child in the classroom.

## Checklist for consent

- Consent form signed by parent (online or hard copy form).
- Child’s clinician details provided, where permission is given.
- Teacher’s contact details provided, where permission is given.

# Methodology

## Assign each family and individual participant a unique code

Once consented, the family and individual participants are formally enrolled into the MAGNET Project and assigned a unique participant identification number. All participants and their families are assigned a unique 8-digit pseudocode identifier, which includes information on 1) study site, 2) study code, 3) family ID, 4) personal ID (i.e., participant, biological mother, biological father, non-biological mother, sibling etc.). These IDs are to be included on all documentation and biological samples. The **Individual** is an 8-digit number, which is a combination of the Family ID and an additional number specific to the individual within the family.

## Participation requirements

Participant visits are scheduled over 2-3 visits, depending upon the number of assessments required. This depends on the participant group (control, clinical), available in-date assessments and the location of the testing sessions (school or Monash University). Depending on the outstanding assessments, the study team may offer to conduct a home visit for the family to reduce participant burden.

The overall commitment to the study differs depending on whether the child is typically developing or has a diagnosis of ASD and/or ADHD, as additional assessments are required.

- Control: 4 hours per child, 2-3 hours of parent questionnaires
- Clinical: 7-9 hours per child, 3.5-4 hours of parent questionnaire

See MAGNET Protocol Summary Table 1 below for child and parent questionnaires, and clinical assessments.

See MAGNET Protocol Summary Table 2 for neurocognitive and eye tracking tasks.

| **MAGNET Protocol Summary Table 1** | | | | | | | | |
| --- | --- | --- | --- | --- | --- | --- | --- | --- |
| **Domain/ Task** | **Duration (mins)** | **Online** | **Control child** | **Control Parents** | **ASD/ADHD**  **(Child or Sib with Dx)** | **ID/ASD/ADHD**  **child** | **Typical Sibling**  **(No Dx)** | **ID/ASD/ ADHD Parents** |
| **Cognitive development** | | | | | | | | |
| WISC, WPPSI, WASI or WAIS | 30-60 |  | X |  | X | X | X |  |
| **Medical/ psychiatric history** | | | | | | | | |
| Subject Medical History Questionnaire | 20 | X | X | X | X | X | X | X |
| Family History Questionnaire | 10 | X | X | X | X | X | X | X |
| Peri/Prenatal environmental questionnaire | 15 | X | X |  | X | X | X |  |
| Children’s Sleep Habits Questionnaire (CSHQ) | 8 | X | X |  | X | X | X |  |
| **Measures of ASD symptoms** | | | | | | | | |
| Autism Diagnostic Observation Schedule  (ADOS-2) | 45-60 |  | X |  | X | X |  |  |
| 3di | 10 |  | X |  | X | X | X |  |
| Social Responsiveness Scale-2nd Edition (SRS-2) | 15 | X | X | X | X | X | X | X |
| Autism Quotient (AQ), Adult, Child | 15 | X | X | X | X | X | X | X |
| Childhood Routines Inventory – Revised (CRI-R) | 10 | X | X |  | X | X | X |  |
| **Dimensional measures of ADHD symptoms** | | | | | | | | |
| Conners’ Parent Rating Scale (CPRS), Conners’ Adult ADHD Rating Scale (CAARS) | 15 | X | X | X | X | X | X | X |
| Strengths and Weaknesses of ADHD symptoms and Normal Behaviour (SWAN) | 5 | X | X |  | X | X | X |  |
| **Comorbidities** | | | | | | | | |
| Development and Well-Being Assessment (DAWBA) | 40-50 | X | X |  | X | X | X |  |
| Children’s Communication Checklist 2 (CCC-2) | 15 | X | X |  | X | X | X |  |
| Aberrant Behaviour Checklist  (ABC) | 15 | X | X |  | X | X | X |  |
| Strengths and Difficulties Questionnaire (SDQ) | 5 | X | X | X | X | X | X | X |
| Child Behaviour Checklist (CBCL) (Parent Report Form & Youth Self Report) | 15 | X | X | X | X | X | X | X |
| Spence Children’s Anxiety Scale (SCAS) | 5 |  | X | X | X | X | X | X |
| Children’s Depression Inventory 2 (CDI-2) (Parent report and Self report) | 10 | X | X | X | X | X | X | X |
| Beck Anxiety Inventory (BAI) | 5 | X |  | X |  |  |  | X |
| Beck Depression Inventory 2 (BDI-2) | 5 | X |  | X |  |  |  | X |
| Adult Routines Inventory (ARI) | 15 | X |  | X |  |  |  | X |
| **Quality of Life/Adaptive Behaviour** | | | | | | | | |
| Vineland Adaptive Behaviour Scale (VABS-3) | 20-45 | X | X |  | X | X |  |  |
| Child-Health and Illness Profile (CHIP-CE) | 12 | X | X |  | X | X | X |  |
| **Language Assessment** |  |  |  |  |  |  |  |  |
| Clinical Evaluation of Language Fundamentals – Fifth Edition (CELF-5) Screening Test | 10-15 |  | X |  | X | X | X |  |
| Clinical Evaluation of Language Fundamentals – Fifth Edition (CELF-5) | 45-90 |  | X |  | X |  | X |  |
| Clinical Evaluation of Language Fundamentals – Preschool Edition (CELF-P2) | 20-40 |  | X |  | X | X | X |  |
| Preschool Language Scale – Fifth Edition (PLS-5) | 35-60 |  |  |  |  | X |  |  |

| **MAGNET Protocol Summary Table 2** | | | | | | | |
| --- | --- | --- | --- | --- | --- | --- | --- |
| **Domain Task** | **Protocol** | **Duration (mins)** | **Age** | **Control child** | **ASD/ADHD** | **ID/ASD/ADHD** | **ID/ASD/ADHD siblings** |
| **Neurocognitive tasks** | | | | | | | |
| Go/No-Go | Monash | 10 | 4+ | X | X | X | X |
| Spatial working memory | LEAP | 10 | 4+ | X | X | X | X |
| Karolinska Directed Emotional Faces | LEAP | 10 | 4+ | X | X | X | X |
| Continuous false belief (sandbox task) | LEAP | 5 | 4+ | X | X | X | X |
| Face recognition | Monash | 5 | 4+ | X | X | X | X |
| Stop signal | Monash | 15 | 8+ | X | X | X | X |
| Reading the Mind in the Eyes | LEAP | 10 | 8+ | X | X | X | X |
| **Eye tracking** | | | | | | | |
| Reflexive saccade task | Monash | 5 | 4+ | X | X | X | X |
| Anti-saccade | Monash | 5 | 8+ | X | X | X | X |
| Smooth pursuit | Monash | 5 | 4+ | X | X | X | X |
| Step-ramp | Monash | 10 | 4+ | X | X | X | X |
| **Total duration for neurocognitive and eye tracking tasks (children aged 4-7)** | **60 min** |  |  |  |  |  |  |
| **Total duration for neurocognitive and eye tracking tasks (children aged 8+)** | **90 min** |  |  |  |  |  |  |

## Pre-site visit

Once eligibility is determined and consent is completed, participants will be contacted to provide further information about the site visit and the questionnaires. Parents are most commonly emailed links to the online questionnaires for ease of access, and so that researchers are able to track what questionnaires have been sent to families. For infection control purposes (see 2.7.2.) parents are asked to complete all questionnaires prior to attending any research sessions, to reduce the amount of contact parents have with staff and supplies/instruments.

### Child and parent 1 questionnaire

The Child and Parent 1 questionnaire is a battery of online clinical rating scales relating to each child and the parent themselves, and is completed by one designated parent, who is defined as Parent 1. The clinical rating scales are standardised measures for a variety of ASD and ADHD traits and common comorbidities. This Child and Parent 1 questionnaire comprises the SRS-2, CPRS, SDQ, DAWBA, SWAN, CRI-R, AQ-C, ABC, CHIP-CE, CSHQ, CBCL, CDI-2, CCC-2 as well as a family and developmental history, and SRS-2, CAARS, SDQ, ARI, BAI, BDI-2, WHOQOL and AQ for Parent 1. Parent 1 must complete the questionnaire prior to the first research visit to minimise missing data. The Parent 2 Questionnaire (if applicable) can be completed before, during or after the testing session(s).

### Sibling questionnaire

The sibling questionnaire is completed by Parent 1 for any other full biological children that are participating in the project. It comprises the SRS-2, CPRS, SDQ, DAWBA, SWAN, CRI-R, AQ-C, ABC, CHIP-CE, CSHQ, CBCL, CDI-2, CCC-2, however it differs from the Child and Parent 1 questionnaire as it does not contain a family history, only a developmental history for that individual child. Further, there are no self-report questionnaires that the parent must complete, as they do so in the Child and Parent 1 questionnaire.

### Vineland Adaptive Behaviour Scale – Third Edition

The Vineland Adaptive Behaviour Scale – Third Edition (VABS-3) includes questions about each child’s adaptive behaviour and how they function in their everyday lives. These can be completed at home prior to the research session(s), or during the research sessions(s) at Monash University. For infection control purposes (see 2.7.2.) parents are emailed a link to the Vineland for each child with the rest of their child, sibling and parent questionnaires. This is to reduce the amount of contact parents have with staff and supplies/instruments.

### Parent 2 questionnaire

The Parent 2 Questionnaire includes a battery of questionnaires solely about that parent, as questions relating to the child only need to be completed once (by Parent 1). This includes SRS-2, CAARS, SDQ, ARI, BAI, BDI-2, WHOQOL and AQ. These questionnaires can be completed at home, at the research visit, or following the research visit. For infection control purposes (see 2.7.2.) parents are asked to complete these questionnaires at home, to reduce the amount of contact parents have with staff and supplies/instruments.

## Site Visit: standardised clinical assessments

All children will complete a cognitive assessment using the Wechsler scales: either a WISC-V (6-16-years), WPPSI-IV (4-6-years), WASI-II (6-90 years) or WAIS-IV (16-90 years). Children entering the study as typically developing, with no current concerns raised about their developmental trajectory, will receive a WASI-II (6 years and over) or WPPSI-IV (4-6-years). The WISC and WPPSI are important clinical assessments for families, clinicians and the education system, therefore children entering the study with a current neurodevelopmental diagnosis, or who have had concerns raised by the parent, school or treating team, will receive a WISC-V or WPPSI-IV depending on their age. All siblings of clinical children complete a WISC or WPPSI because of the increased likelihood that we may uncover that the child meets criteria for ASD or ADHD and may need to be seen by follow up clinical services. Parent 1 will complete a Vineland questionnaire for all participating children. All children aged 5 and older will complete a CELF-5 Screener assessment. Children who reach a threshold on the CELF-5 Screener, or otherwise who are indicated by clinical judgement during cognitive assessment and/or the ADOS will be complete a CELF-5 (5 – 21 years), CELF-P2 (3 – 6 years), or a PLS-5 (minimally verbal children aged, 0 – 7 years).

These measures are routinely used in clinics and research studies (e.g., 3di, ADOS-2, WASI). To reduce participant burden, the results of these assessments can be utilised in lieu of administering another. WASI-II (or WISC-V/WAIS-IV) scores may be reused if the previous assessment took place within 2 years. ADOS scores may be reused if the assessment took place no longer 6 months (Modules 1, 2, 3, 4). The 3di scores may be used from previous assessments carried out at any age provided that all scores are available. CELF-5, CELF-P2, and PLS-5 scores may be reused if the assessment took place in the last 12 months. Unless otherwise specified, standardized measures are administered in line with the published instructions from the manual.

### Wechsler Abbreviated Scales of Intelligence - Second Edition

The WASI-II will be used for all participants aged above 6 years without a diagnosis or suspected diagnosis of ADHD and/or ASD, the four-subtest form includes two verbal subscales (Vocabulary, Similarities) and two non-verbal subscales (Block Design, Matrix Reasoning). On this basis, a full-scale IQ (FSIQ) score can be derived, which serves as an index of general cognitive ability. Subtest standard scores and PIQ, VIQ and FSIQ standardized scores will be entered into the database. Participants who complete the WASI-II will also complete the Digit Span subtest from the WISC-V or the WAIS-IV (dependent on participant age) as a measure of verbal working memory.

### Wechsler Preschool and Primary Scale of Intelligence - Fourth Edition

The WPPSI-IV is administered for all participants aged less than 6 years. The ten primary subtests will be completed: these subtests map to Verbal Comprehension, Visual Spatial, Fluid Reasoning, Working Memory, and Processing Speed indices. These will also give a FSIQ, which is used as an index of general cognitive ability.

If a child is non-verbal or has suspected Intellectual Disability, it may not be appropriate or possible to administer the age appropriate Wechsler test. In the advent of this, subtests from the WPPSI-IV should be first attempted. The recommended WPPSI-IV subtests are Picture Naming, Receptive Vocabulary, Object Assembly, Block Design, Information and Bug Search. The Raven’s coloured progressive matrices may also be attempted.

### Wechsler Intelligence Scale for Children - Fifth Edition

The WISC-V is administered for participants aged 6 - 16 years with a diagnosis or suspected diagnosis of ADHD and/or ASD, and their siblings irrespective of diagnostic status. The ten subtests will be completed: these subtests map to Verbal Comprehension, Visual Spatial, Fluid Reasoning, Working Memory, and Processing Speed indices. These will also give a FSIQ, which is used as an index of general cognitive ability.

### Wechsler Adult Intelligence Scale - Fourth Edition

The WAIS-IV is administered for all participants aged 16 years or older with a diagnosis or suspected diagnosis of ADHD and/or ASD and their siblings irrespective of diagnostic status. It provides measures of 4 main areas of intelligence: Verbal Comprehension, Perceptual Reasoning, Working Memory and Processing Speed.

A Full Scale Intelligence Quotient (FSIQ) is derived by combining 10 subtests to give a measure of a participant’s overall cognitive abilities.

### Vineland Adaptive Behaviour Scale – 3

The VABS-3 (Parent/ Caregiver Interview form) will be used to probe for personal and social skills, including socialization, communication, daily living, and motor skills if the child is 9 years old or younger. The VABS-3 will be administered to the parent or caregiver during the study visit. The scores entered into the database will be the domain-standardized scores and age equivalents (socialization, communication, daily living skills, and motor skills where applicable) and the Adaptive Behaviour Composite. The VABS-3 will also be completed by parents with typically developing children.

### The Developmental, Dimensional and Diagnostic Interview (3di)

The 3di is a standardised, dimensional, computer-based, diagnostic interview to assist with the diagnosis of ASD. The interview will be typically administered via a computer for the MAGNET Project. Where this is not possible, the interview will be conducted during a separate home visit or over the telephone. The 3di questions are designed to assist in the identification of ASD, including questions on language development and early ASD symptomatology. For newly completed 3dis, all item-level scores should be entered.

### Autism Diagnostic Observation Schedule – Second Edition

The ADOS-2 is a standardized protocol for observation of social, communicative and repetitive behaviours associated with autism. The instrument comprises a series of structured and semi-structured presses for interaction, accompanied by coding of specific target behaviours associated with particular tasks and by general ratings of the quality of behaviours.

Modules 1-4 will be used, depending on the participant’s age, and expressive language level. If a participant has already completed either an ADOS-2 or an ADOS-G, these scores can be utilised rather than readministering. ADOS-G cut-offs are calculated for the social and communicative domains and a combined cut-off for the sum of social and communication scores. ADOS-2 cut-offs are calculated for the social and affect (SA) and restricted and repetitive behaviour (RRB) domains and a combined cut-off for the sum of SA and RRB scores. We will *video-record* all ADOS sessions to enable scoring by a second-rater, with final scores determined through consensus coding. One rater will be blinded to child’s diagnostic status for the purposes of scoring ADOS-2 assessments.

For newly completed ADOSs, all item-level scores should be entered, as should the domain and diagnostic algorithm scores. For previously completed ADOS scores to be eligible, the domain and diagnostic algorithm scores should be completed withing the past 6 months.

### Clinical Evaluation of Language Fundamentals – Fifth Edition Screening Test

The Clinical Evaluation of Language Fundamentals – Fifth Edition Screening Test (CELF-5 Screener) is a sensitive language screening tool for children aged 5 to 21 years that identifies the need for a comprehensive language assessment. All children complete a CELF-5 Screener. Children who score at or below their age-referenced criterion score are referred for a comprehensive language evaluation. To ensure the dimensionality of language difficulties are captured, children whose scores are 2 points above criterion and/or who showed difficulty in any of the subtests, during the cognitive assessment, or during the ADOS are also administered a comprehensive language assessment (CELF-5, CELF-P2, or PLS-5).

### Clinical Evaluation of Language Fundamentals – Fifth Edition

The Clinical Evaluation of Language Fundamentals - Fifth Edition (CELF-5) is an instrument for identifying and diagnosing disorders in language performance. The CELF-5 is structured around four levels of assessment: language disorder, nature of disorder, behaviours of the disorder, and how the disorder affects classroom performance.

There are four Core tests used to determine the deficit; additional sub-tests determine strengths and weaknesses, and supplemental sub-tests for clinical skills. The Pragmatics Profile allows for a descriptive measure of language performance at school and at home.

### Clinical Evaluation of Language Fundamentals – Preschool Edition

The Clinical Evaluation of Language Fundamentals – Preschool Edition (CELF- P2) is a comprehensive language assessment designed to identify language strengths and weaknesses in preschool children aged 3 to 6.11 years. However, it is expected that there will be participants between 6.0 - 6.11 years old who are presenting with significant language difficulties (e.g. Intellectual Disability and Language Disorders) where this assessment tool will be more appropriate and increase likelihood of completing the assessment. In these instances, the CELF-P2 will be administered instead of the CELF-5.

### Preschool Language Scaled – Fifth Edition (PLS-5)

The Preschool Language Scales – Fifth Edition (PLS-5) is a measure of language, incorporating information from clinician observation, direct measurement and parent report. The PLS-5 is to be administered in line with administration guidelines from the manual. PLS-5 is administered to younger minimally verbal children in the age range of birth to 7.11years and consists of two standard scales: Auditory comprehension and Expressive communication. This assessment will be administered should a child present with limited language skills (e.g. non-verbal) and is unable to complete the CELF-P2.

### Intra-site reliability

For the ADOS-2, all administrators will be trained to research reliability by trainers at their site, or on another recognised course. All administrators at all sites will attend local reliability coding meetings (not necessarily specific to, but including some tapes from the study) run by recognized ADOS-2 trainers.

### Best estimate of clinical diagnosis

Standardised clinical data collected during the project, and previous clinical reports provided by parents where necessary, will be reviewed by the team’s paediatrician and psychologist, and speech pathologist if available, to determine a best estimate of clinical diagnosis for the child at the time of assessment in the project. This will include evaluation for the following diagnoses: ASD, ADHD, comorbid ASD/ADHD, Intellectual Disability, Oppositional Defiance Disorder, Conduct Disorder, anxiety, depression, and language disorder. This estimate will be stored in the project’s REDCap database.

## Site visit: Neurocognitive Testing

The child will complete neurocognitive tasks that index ASD and ADHD related processes. They will also complete eye-tracking tasks. Depending on the location of testing, the order of administration will differ.

### Go/No-Go


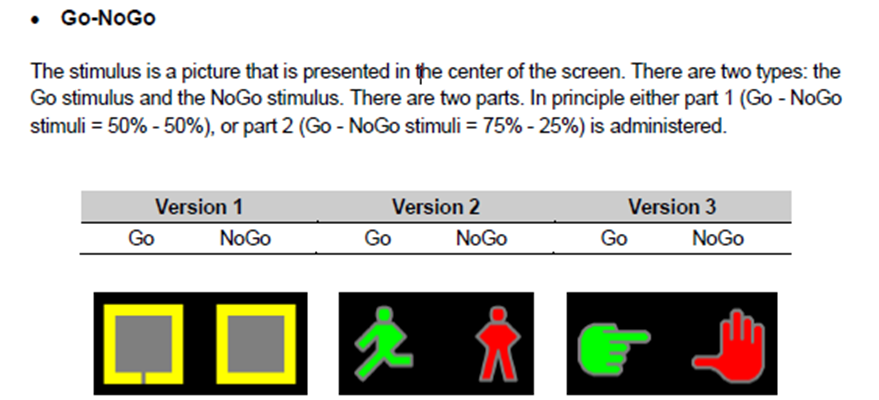
The Go/No-Go Task measures sustained attention and response inhibition (Amsterdam Neuropsychological Tasks; De Sonneville, 1999). The participant will be shown either a Green ‘Go’ Signal or a Red ‘No-Go’ Signal and must press the spacebar as quickly as they can when the Go signal is presented.

### Face Recognition

The Face Recognition Task (Amsterdam Neuropsychological Tasks; De Sonneville, 1999) explores the participant’s ability to remember and recognise faces. The participant will be shown a picture of one face which disappears, and then they must decide whether that face is present in a set of four pictures.


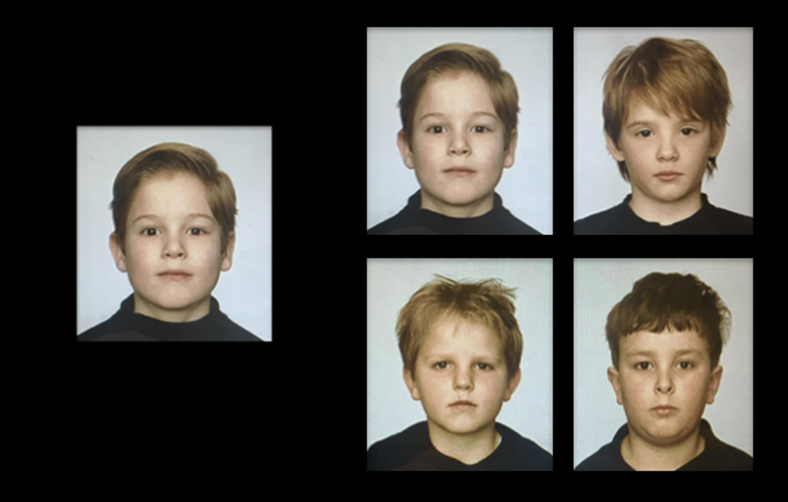


### Spatial Working Memory – Find the Phone

The Spatial Working Memory task explores the participant’s spatial working memory, and their strategy. The participant must find the phone that is ringing, remembering which phones have rung before.


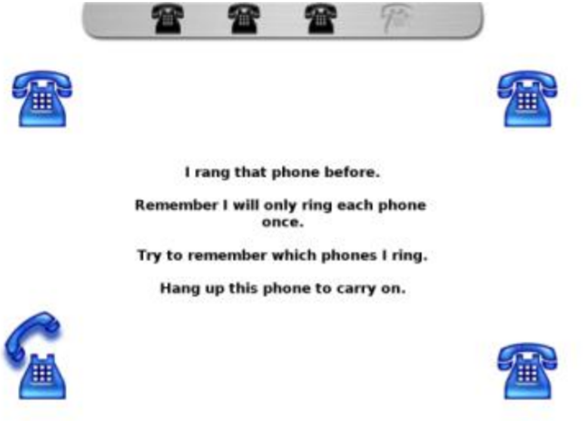


### Karolinska Directed Emotional Faces

The Karolinska Directed Emotional Faces explores the participant’s ability to recognise emotion in an entire face. The participant must select which emotion they think best matches the picture of the face.


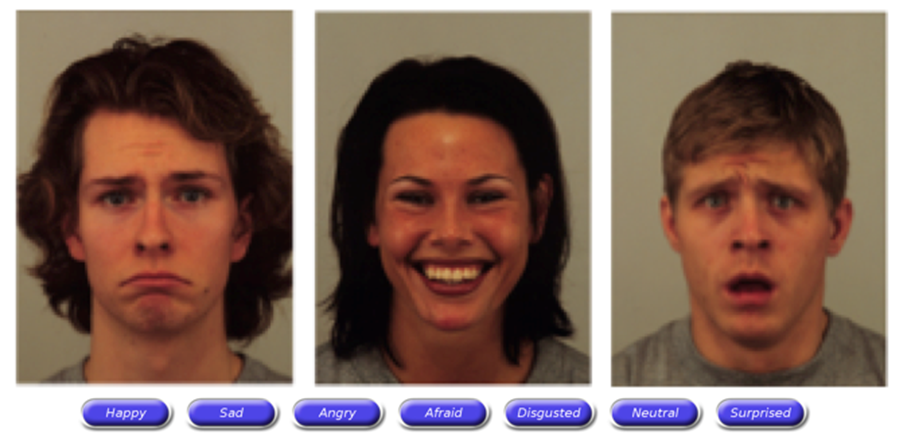


### Reading the Mind in the Eyes Task – Child

The Reading the Mind in the Eyes Task explores the participant’s ability to recognise emotion when presented with pictures of eyes. The participant must select what they think the person is thinking or feeling.


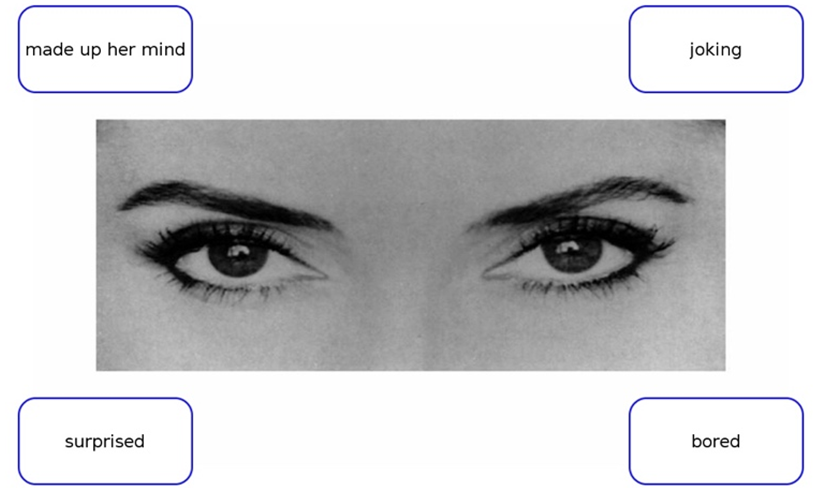


### Continuous False Belief – Sandbox Task

The Sandbox Task provides an indication of Theory of Mind (Beeger et al., 2012). Theory of Mind is the ability to attribute emotions and beliefs to other people, and to understand that these may differ from our own (Korkmaz, 2011; Premack & Woodruff, 1978). This task can be completed using pen and paper, or on a touch-screen computer. The researcher presents the story to the participant on a printed page or as an image on the computer screen and reads the story aloud: the story is about a girl and her father who are planting flower bulbs. While the father is absent, the girl moves the flower bulb to a different location (AB – False Belief Condition) or buries a stone in a different location (BA – True Belief Condition). The participant is then asked to show where the father thinks the flower bulb is planted, by marking the location with an ‘X’ (on either the paper page or the touch-screen image).


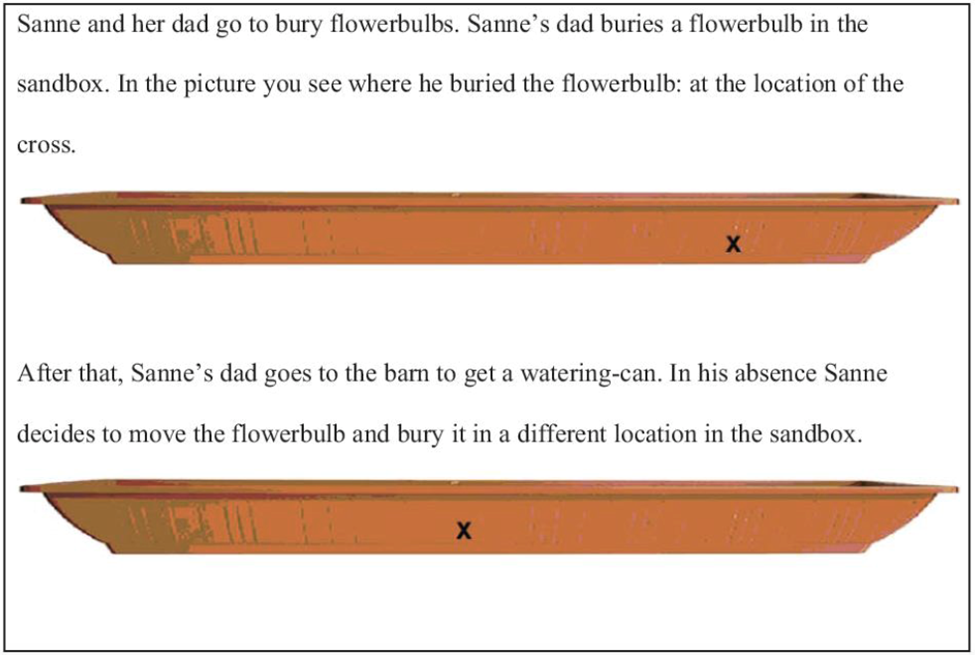


False belief condition (AB)


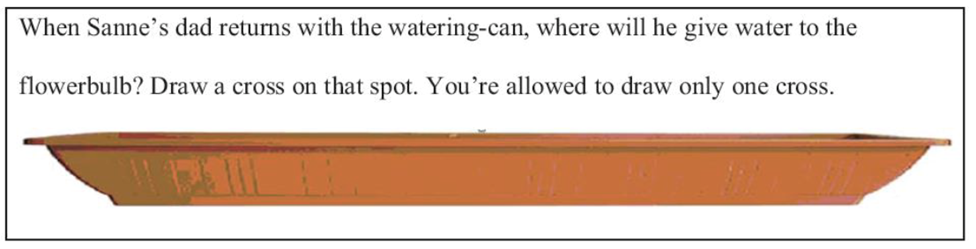


Question asked to participant following AB or BA.

### New Cambridge Gambling Task

The New Cambridge Gambling Task explores decision making and risk taking. Children must choose what colour they think the spinning pointer will land on and place a “guess” using “tokens”.


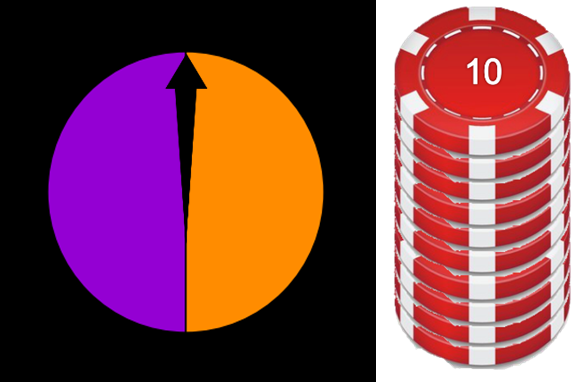


### Stop Signal Task

The Stop Signal Task explores reaction time and inhibition. The participant is presented with a series of circles and squares that appear on the screen, one after the other. When each stimulus appears (circle or square), the participant must respond as quickly as possible by pressing a designated key. However, when a tone is played along with the presentation of the stimulus, participants must not press the key, and rather inhibit their response.

### New Reversal Learning

The New Reversal Learning task explores the participant’s response following positive and negative feedback. The participant must select either the yellow or blue pattern, where they are told that one is correct more often than the other.


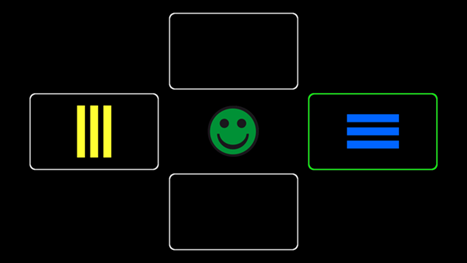


Positive feedback.


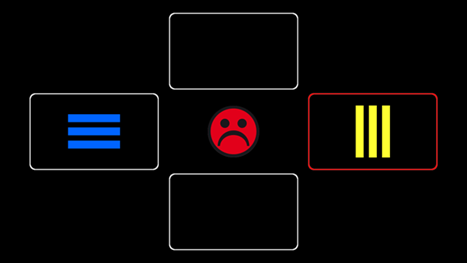


Negative feedback.

### Spence Children’s Anxiety Scale

The Spence Children’s Anxiety Scale is a self-report measure completed by the child. It asks how often they worry about each of the different scenarios. This scale can be completed on hardcopy or input directly into the participant’s record in REDCap. The researcher may complete this scale verbally with participants if their reading level prevents them from completing it independently.

### Child’s Depression Inventory 2 (CDI-2)

The Child’s Depression Inventory 2 is a multi-rater assessment of depressive symptoms in children aged 7 to 17 years. The child completes a 28-item self-report form, where the child is asked to select 1 statement from 3 options within each item. The statements are about the child’s thoughts and feelings in the last 2 weeks. Parents complete a complementary 17-item form, which asks the parent to rate their child’s behaviour and thoughts in the last 2 weeks, on a 4-point scale. The child’s self-report form can be completed on a hardcopy or input directly into the participant’s record in REDCap. For children whose reading level may prevent them for reading and/or understanding the scale, the researcher may complete the scale verbally with the participants. The parent form is completed electronically, as part of the Child and Parent 1 Questionnaire (see Section 7.3.1).

### Beighton’s Hypermobility Scale

The Beighton’s hypermobility scale measures a child’s degree of hypermobility. Included within the CRF are instructions about how to measure hypermobility. Nine measurements are taken, and the researcher asks the child whether they are able to do each action (e.g. touch the floor with their legs straight), and the researcher must write either Y or N whether the child was able to do this.

### Physical Measurements

The child’s height, head circumference, and weight must all be measured at the research visit and entered into the CRF.

## Site visit: Eyetracking

### Visually guided saccade

This task explores visually guided (or reflexive) saccades: these are initiated in response to novel exogenous stimuli. The participant must follow the green cross on the screen with their eyes.

### Antisaccade paradigm

This task requires the participant not to look at the green cross on the screen, but instead to look at the mirror opposite position, thus to inhibit a reflexive saccade.


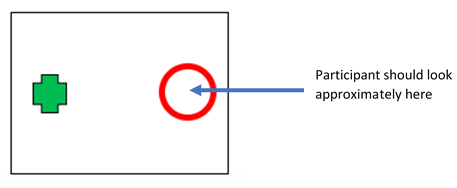


### Smooth pursuit 1: Smooth pursuit eye movement

This task assesses how well the participant is able to smoothly follow an object as it moves across the screen, and if they need to make catch-up saccades.

### Smooth pursuit 2: Step ramp

This task assesses how well the participant is able to smoothly follow an object as it moves across the screen, and if they need to make catch-up saccades.

## Salivary DNA Sample

Saliva samples will be collected from Caucasian families. Participants will be asked to collect a saliva sample using an ORAgene DNA Saliva Kit, or ORACollect for Paediatrics kit. Saliva samples will typically be collected during the research visit. Alternatively, if the parents are not in attendance (e.g. school visit, or only one parent attending research visit at Monash University), a saliva kit can be sent home via the mail, or with the family. See section 7.7.2 for further detail on mailing saliva kits.

Participants are required to provide a small (2 ml) sample of saliva from which DNA can be extracted. This is a non-invasive procedure and is thus preferable to the acquisition of blood in child populations. All DNA will be stored for a minimum of 10 years in our secure, restricted-access laboratory, and will only be destroyed upon request. Storage of any paper and electronic data collected will adhere to the University regulations and kept on University premises. Retaining DNA and data over the longer term will maximise the value of the effort on the part of the participants and their families, and the financial investment of our funding bodies, and will afford the sample size required to allow true scientific breakthroughs regarding the genetics of ASD and ADHD to occur.

The collection of DNA and behavioural data from children with ASD, ADHD and healthy control participants is a time-consuming and expensive process and forms an enormously valuable resource. DNA sample and data may be used in future, ethically approved genetic studies of ASD and ADHD, and may be pooled together with other genetic data from our domestic and/or international collaborators. Data may only be accessed where ethics approval to use the data has been granted by a governing research institute to the researcher/s, and data may only be used for non-commercial purposes. No information that could potentially identify an individual participant would be shared or made public.

### In person saliva collection

Prior to collecting a saliva sample, the participant is required to rinse out their mouth with water and refrain from food and water for at least 30 mins. This is to avoid the presence of food or remnants in the sample. Participants will provide a saliva sample using an ORAgene-DNA or ORACollect Paediatrics collection kit. The ORAgene-DNA kits will be used most frequently and require participants to provide a 2 mL sample. The ORACollect for Paediatrics kit is a buccal swab kit that will be used when a participant is having difficulty producing enough saliva. To minimise the risk of potential disease transmission, research team members will wear latex gloves and participants will collect the samples themselves, and place them directly into a labelled, rigid plastic storage box (the secondary container, consistent with guidelines for transportation and storage of biological hazards). The tube and container will both be labelled with the participant ID and their initials. See the SOP for Saliva Collection, Handling and DNA Extraction for further information regarding saliva collection procedures.

### Mail-out saliva kits

Saliva kits can be mailed (with a prepaid return envelope) to family members who did not attend the scheduled testing sessions, so that they can still participate in the genetic component of the project. See the SOP for Saliva Collection, Handling and DNA Extraction for further information regarding saliva kit mailing procedures. These kits can be sent before or after the study visit.

# Brain structure and function

Structural and functional brain imaging (resting state fMRI) will be collected to determine if neurobiological differences exist as a function of symptom-based data-driven clusters. All scans will be performed using Siemens Skyra 3T scanner following previously established protocols (Oldham et al., 2020; Sabaroedin et al., 2019). Data processing pipelines will include extensive correction for in-scanner motion (Oldham et al., 2020; Parkes, Fulcher, Yücel, & Fornito, 2018) which is the most prevalent MRI artefact in paediatric populations.

# Clinical Report

Upon the completion of the family’s participation in the study, the child will receive a clinical report which summarises the clinically relevant information collected during the study. This includes results from the child’s cognitive assessment, SRS-2, CPRS, Vineland, language assessment (CELF-5 Screener and CELF-5/CELF-P2/PLS-5 if applicable), SCAS, CDI, and the ADOS-2. Within this report it is clearly stated that these results were obtained for the purpose of research and are not diagnostic: results must be interpreted by an applied professional, e.g. paediatrician, psychologist, psychiatrist. The supervising psychologist reviews the clinical report before it is distributed to the family.

The family will receive the clinical report approximately two months following the completion of all assessments for typically developing child, and between three-four months for a child with ASD and/or ADHD.

# Analysis methods

A combination of supervised psychometric analyses and unsupervised clustering approaches will be used to converge on data-driven homogeneous ASD-ADHD clusters embedded within biologically-relevant dimensions based on previously derived factor score estimates (Borsboom et al., 2016; Feczko et al., 2019). By using multiple measures of target constructs to create latent variable phenotypes, we can maximise our study’s statistical power and strengthen the representation of our key constructs (van der Sluis, Verhage, Posthuma, & Dolan, 2010). Obtaining information from multiple informants controls for informant bias, whilst discrepancies between informant reports provides additional sources of information relevant to developmental psychopathology that can be the subject of further analysis (De Los Reyes, Salas, Menzer, & Daruwala, 2013; De Los Reyes, Thomas, Goodman, & Kundey, 2013). Moreover, the MAGNET Project’s representative sample and measures are important prerequisites for robust clustering methods to avoid model overfitting and poor reproducibility (Bzdok, Altman, & Krzywinski, 2018; Rashid & Calhoun, 2020).

Dimension reduction strategies, such as exploratory factor analysis and exploratory structural equation modelling (Asparouhov & Muthén, 2009; Costello & Osborne, 2005; Marsh, Morin, Parker, & Kaur, 2014), or multidimensional item response theory (Reckase, 2009), will be used on each participant’s raw scores to first identify their factor or scale score estimates representing their standing on these latent dimensions. Unbiased feature selection and optimising latent model fit in this step, prior to later clustering analyses, can reduce the interference of variance from extraneous noise. It is also acknowledged that there may be clustering and nesting within the data based on sampling (e.g. participants from the same family) and testing (e.g. testing sessions, assessors) procedures (Clarke, 2008; McNeish, 2014). Subsequent analyses will account for these effects, though the choice of correction method will depend on the characteristics of our final dataset.

Factor mixture modelling is one possible supervised clustering method that we will employ for our subtyping analyses. Factor mixture modelling can uncover homogeneous clusters within continuous and categorical data embedded within dimensional models of psychopathology by utilising probabilistic modelling techniques (Borsboom et al., 2016; Lubke & Muthén, 2005; Miettunen et al., 2016). The flexibility of factor mixture modelling permits the testing and comparison of multiple models with varying numbers of *a priori* specified clusters. Alternatively, where unsupervised machine learning techniques may be better suited for addressing specific research questions, community detection is one possible approach. This method combines graph theoretic analyses to detect homogeneous communities/clusters (i.e. highly connected sets of nodes). By ensuring that the algorithm achieves a connected graph, our analyses will parsimoniously account for all participants. These approaches empirically unify the theoretical grounding of MAGNET’s research questions with the power of cutting-edge data-driven analysis techniques. Moreover, both techniques are diagnosis-naïve, thus allowing MAGNET to fully embrace the transdiagnostic features of our biobehavioural subtypes. Finally, although MAGNET aims towards data-driven clusters using symptom and behavioural data, the potential utility of incorporating neurocognitive or genetic components in defining clusters will not be overlooked (Clementz et al., 2016; Fair, Bathula, Nikolas, & Nigg, 2012).

# References

Asparouhov, T., & Muthén, B. (2009). *Exploratory structural equation modeling*. *Structural Equation Modeling* (Vol. 16). https://doi.org/10.1080/10705510903008204

Borsboom, D., Rhemtulla, M., Cramer, A. O. J., Maas, H. L. J. Van Der, & Scheffer, M. (2016). Kinds versus continua: A review of psychometric approaches to uncover the structure of psychiatric constructs, (2016), 1567–1579. https://doi.org/10.1017/S0033291715001944

Bzdok, D., Altman, N., & Krzywinski, M. (2018). Statistics versus machine learning. *Nature Methods*, *15*(4), 233–234. https://doi.org/10.1038/nmeth.4642

Clark, S. L., Muthén, B., Kaprio, J., Onofrio, B. M. D., Viken, R., Rose, R. J., … Rose, R. J. (2013). Models and strategies for factor mixture analysis : An example concerning the structure underlying psychological disorders. *Structural Equation Modeling: A Multidisciplinary Journal*, *20*(4), 681–703. https://doi.org/10.1080/10705511.2013.824786

Clarke, P. (2008). When can group level clustering be ignored? Multilevel models versus single-level models with sparse data. *Journal of Epidemiology and Community Health*, *62*(8), 752–758. https://doi.org/10.1136/jech.2007.060798

Clementz, B. A., Sweeney, J. A., Hamm, J. P., Ivleva, E. I., Ethridge, L. E., Pearlson, G. D., … Tamminga, C. A. (2016). Identification of distinct psychosis biotypes using brain-based biomarkers. *American Journal of Psychiatry*, *173*(4), 373–384. https://doi.org/10.1176/appi.ajp.2015.14091200

Costello, A. B., & Osborne, J. W. (2005). Best practices in exploratory factor analysis: Four recommendations for getting the most from your analysis. *Practical Assessment, Research and Evaluation*, *10*(7). https://doi.org/https://doi.org/10.7275/jyj1-4868

De Los Reyes, A., Salas, S., Menzer, M. M., & Daruwala, S. E. (2013). Criterion validity of interpreting scores from multi-informant statistical interactions as measures of informant discrepancies in psychological assessments of children and adolescents. *Psychological Assessment*, *25*(2), 509–519. https://doi.org/10.1037/a0032081

De Los Reyes, A., Thomas, S. A., Goodman, K. L., & Kundey, S. M. A. (2013). Principles underlying the use of multiple informants’ reports. *Annual Review of Clinical Psychology*, *9*(1), 123–149. https://doi.org/10.1146/annurev-clinpsy-050212-185617

De Sonneville, L. M. J. (1999). Amsterdam Neuropsychological Tasks: A computer-aided assessment program. *Computers In Psychology*, *6*, 187–203.

Fair, D. A., Bathula, D., Nikolas, M. A., & Nigg, J. T. (2012). Distinct neuropsychological subgroups in typically developing youth inform heterogeneity in children with ADHD. *Proceedings of the National Academy of Sciences of the United States of America*, *109*(17), 6769–6774. https://doi.org/10.1073/pnas.1115365109

Feczko, E., Miranda-Dominguez, O., Marr, M., Graham, A. M., Nigg, J. T., & Fair, D. A. (2019). The heterogeneity problem: Approaches to identify psychiatric subtypes. *Trends in Cognitive Sciences*, *23*(7), 584–601. https://doi.org/10.1016/j.tics.2019.03.009

Grzadzinski, R., Di Martino, A., Brady, E., Mairena, M. A., O’Neale, M., Petkova, E., … Castellanos, F. X. (2011). Examining autistic traits in children with ADHD: Does the Autism Spectrum extend to ADHD? *Journal of Autism and Developmental Disorders*, *41*(9), 1178–1191. https://doi.org/10.1007/s10803-010-1135-3

Harris, P. A., Taylor, R., Minor, B. L., Elliott, V., Fernandez, M., O’Neal, L., … Duda, S. N. (2019). The REDCap consortium: Building an international community of software platform partners. *Journal of Biomedical Informatics*, *95*, 103208. https://doi.org/10.1016/j.jbi.2019.103208

Harris, P. A., Taylor, R., Thielke, R., Payne, J., Gonzalez, N., & Conde, J. G. (2009). Research electronic data capture (REDCap)— A metadata-driven methodology and workflow process for providing translational research informatics support. *Journal of Biomedical Informatics*, *42*(2), 377–381.

Hawi, Z., Cummins, T. D. R., Tong, J., Johnson, B., Lau, R., Samarrai, W., & Bellgrove, M. A. (2015). The molecular genetic architecture of attention deficit hyperactivity disorder. *Molecular Psychiatry*, *20*(3), 289–297. https://doi.org/10.1038/mp.2014.183

Lubke, G. H., & Muthén, B. (2005). Investigating population heterogeneity with factor mixture models. *Psychological Methods*, *10*(1), 21–39. https://doi.org/10.1037/1082-989X.10.1.21

Marsh, H. W., Morin, A. J. S., Parker, P. D., & Kaur, G. (2014). Exploratory structural equation modeling: An integration of the best features of exploratory and confirmatory factor analysis. *Annual Review of Clinical Psychology*, *10*(Mimic), 85–110. https://doi.org/10.1146/annurev-clinpsy-032813-153700

McNeish, D. M. (2014). Modeling sparsely clustered data: Design-based, model-based, and single-level methods. *Psychological Methods*, *19*(4), 552–563. https://doi.org/10.1037/met0000024

Miettunen, J., Nordström, T., Kaakinen, M., & Ahmed, A. O. (2016). Latent variable mixture modeling in psychiatric research: A review and application, (2016), 457–467. https://doi.org/10.1017/S0033291715002305

Oldham, S., Arnatkevic̆iūtė, A., Smith, R. E., Tiego, J., Bellgrove, M. A., & Fornito, A. (2020). The efficacy of different preprocessing steps in reducing motion-related confounds in diffusion MRI connectomics. *NeuroImage*, *222*(July), 117252. https://doi.org/10.1016/j.neuroimage.2020.117252

Parkes, L., Fulcher, B., Yücel, M., & Fornito, A. (2018). An evaluation of the efficacy, reliability, and sensitivity of motion correction strategies for resting-state functional MRI. *NeuroImage*, *171*(December 2017), 415–436. https://doi.org/10.1016/j.neuroimage.2017.12.073

Polanczyk, G. V., Willcutt, E. G., Salum, G. A., Kieling, C., & Rohde, L. A. (2014). ADHD prevalence estimates across three decades: An updated systematic review and meta-regression analysis. *International Journal of Epidemiology*, *43*(2), 434–442. https://doi.org/10.1093/ije/dyt261

Randall, M., Sciberras, E., Brignell, A., Ihsen, E., Efron, D., Dissanayake, C., & Williams, K. (2016). Autism spectrum disorder: Presentation and prevalence in a nationally representative Australian sample. *Australian & New Zealand Journal of Psychiatry*, *50*(3), 243–253. https://doi.org/10.1177/0004867415595287

Rashid, B., & Calhoun, V. (2020). Towards a brain-based predictome of mental illness. *Human Brain Mapping*, *41*(12), 3468–3535. https://doi.org/10.1002/hbm.25013

Reckase, M. D. (2009). Multidimensional Item Response Theory. New York, NY: Springer.

Rommelse, N. N. J., Franke, B., Geurts, H. M., Hartman, C. A., & Buitelaar, J. K. (2010). Shared heritability of attention-deficit/hyperactivity disorder and autism spectrum disorder. *European Child and Adolescent Psychiatry*, *19*(3), 281–295. https://doi.org/10.1007/s00787-010-0092-x

Sabaroedin, K., Tiego, J., Parkes, L., Sforazzini, F., Finlay, A., Johnson, B., … Fornito, A. (2019). Functional connectivity of corticostriatal circuitry and psychosis-like experiences in the general community. *Biological Psychiatry*, *86*(1), 16–24. https://doi.org/10.1016/j.biopsych.2019.02.013

Thomas, S., Sciberras, E., Lycett, K., Papadopoulos, N., & Rinehart, N. (2015). Physical functioning, emotional, and behavioral problems in children with ADHD and comorbid ASD: A cross-sectional study. *Journal of Attention Disorders*, *0*(0), 1087054715587096. https://doi.org/10.1177/1087054715587096

van der Sluis, S., Verhage, M., Posthuma, D., & Dolan, C. V. (2010). Phenotypic complexity, measurement bias, and poor phenotypic resolution contribute to the missing heritability problem in genetic association studies. *PLoS ONE*, *5*(11). https://doi.org/10.1371/journal.pone.0013929
