# Supplementary material for: The Monash Autism-ADHD genetics and neurodevelopment (MAGNET) project design and methodologies: a dimensional approach to understanding neurobiological and genetic aetiology
Source: Mol Autism. 2021 Aug 5;12:55. doi: 10.1186/s13229-021-00457-3 (PMC8340366; doi:10.1186/s13229-021-00457-3)
Supplement: Supplementary file 2 — Additional file 2. Best clinical estimate protocol. [file 13229_2021_457_MOESM2_ESM.docx]

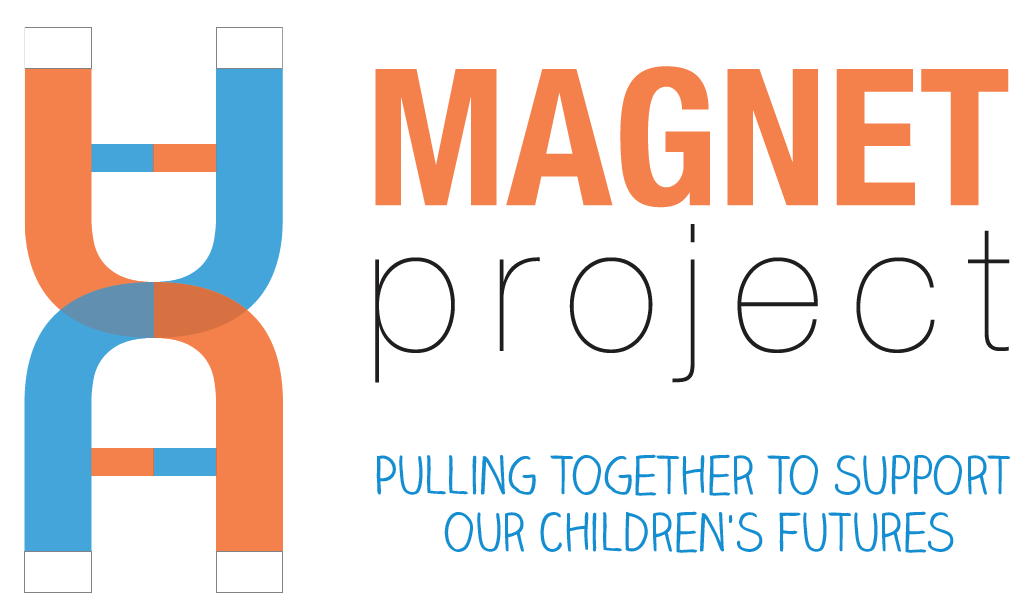


Best Clinical Estimate (BCE) SoP

The Monash Autism/ADHD Genetics and Neurodevelopment (MAGNET) Project

Principal investigators

Dr Beth Johnson, Prof Mark Bellgrove

Turner Institute of Brain and Mental Health

Monash University

Date: September 2020

Revision: 1

Confidential

This document is confidential. It may not be transmitted, reproduced, published, or used without prior written authorization.

Statement of Compliance

This document is a protocol for a research project. This study will comply with this protocol, the conditions of the ethics committee approval, and the NHMRC National Statement on ethical Conduct in Human Research (2018).

**Best Clinical Estimate (BCE)**

# Overview

A Best Estimate Clinical (BCE) is recognised as an important component of the assessment protocol, especially in genetics studies (Baron, Endicott, & Ott, 1990). In research, when assessing conditions such as autism spectrum disorder (ASD) and attention-deficit/ hyperactivity disorder (ADHD) there is often a heavy reliance on parent-report to determine a child’s diagnostic status. A BCE is intended to reduce diagnostic error in research studies, with an increased level of certainty in a child’s diagnosis as reported by the parents.

The BCE is not a formal diagnosis, but an independent review whereby a ‘best diagnostic impression’ is determined. In a research setting this process aims to ensure that participants are classified as accurately as possible. Best practice guidelines stipulate the opinion of at least a registered psychologist and a paediatrician should be considered in the diagnosis of ASD and ADHD.

The BCE is not a formal diagnosis, but an independent review to ensure that participants are grouped to appropriate comparative samples for the MAGNET Project Study Design (e.g. ASD, ADHD, ASD/ADHD combined, intellectual disability, ODD, CD, anxiety, depression, language disorders, and typical development).

All children (typically developing, probands, and siblings) enrolled in the MAGNET Project will undergo the BCE protocol irrespective of diagnostic status on enrolment.

## BCE Team:

MAGNET Project BCE team consists of:

1. Qualified, registered Psychologist (Clinical, Educational & Developmental, or Neuropsychologist)
2. Paediatrician, and
3. Speech Pathologist (if available).

Specialists will review all available clinical information for each participant in order to assign the best diagnostic impression on the basis of information collected as part of the MAGNET project. The measures included for review are outlined below.

## Summary BCE Report

A BCE Report is completed for every child and is to be completed by at least a qualified psychologist (Clinical, Educational & Developmental, Neuropsychologist) and paediatrician, with provision for additional input from a speech pathologist.

Each member of the BCE team completes a BCE Report independently for each child. The clinicians then discuss their diagnostic impression and come to a consensus BCE.

Children are reviewed against DSM-5 and ICD-11 diagnostic criteria for the diagnostic categories of interest.

# Measures for Inclusion in BCE Review

## Parent-report measures

BCE reviewers will be provided with:

### Child Behaviour Checklist (CBCL)

For the parent-report version of the CBCL, reviewers will be provided with T-scores and qualitative ranges for the anxious, depressed, social problems, thought problems, oppositional defiant problems, conduct problems, and attention problems subscales.

### Child Communication Checklist – Second Edition (CCC-2)

Scores from the General Communication Composite and the Social Interaction Difference Index are provided for BCE reviewers.

### Children’s Depression Inventory – Second Edition (CDI-2)

For the parent-report version of the CDI-2, scaled scores and qualitative ranges for the emotional problems, functional problems, and total scale scores, in addition to the negative mood, negative self-esteem, ineffectiveness, and interpersonal problems subscale scores.

### Conners’ Parent Rating Scale – Revised Long Form (CPRS-R-LF)

Summarised table including T-score and qualitative ranges for the inattentive subscale, hyperactive/impulsive subscale, ODD subscale, and total score.

### The Developmental, Dimensional and Diagnostic Interview (3di)

Reviewers are to be provided with item level responses from the 3di as reported by the parent/caregiver.

### The Development and Wellbeing Assessment (DAWBA)

Reviewers are to be provided with item level responses from the DAWBA as reported by the parent/caregiver.

### Spence Children’s Anxiety Scale (SCAS)

For the parent-report version of the SCAS, reviewers will be provided with T-scores and qualitative ranges for the generalised anxiety, physical injury fears, panic/agoraphobia, obsessive compulsive, social phobia, and separation anxiety subscales, and the total score.

### Social Responsiveness Scale – Second Edition (SRS-2)

Summarised table including T-scores and qualitative ranges for the social awareness, social cognition, social communication, social motivation, and restricted interests and repetitive behaviours subscales, the social communication index, and for the total score.

### Vineland – 3

Reviewers are provided standard scores, 95% confidence intervals, percentile ranks (where applicable), and qualitative ranges for the Adaptive Behaviour Composite, domain scores (communication, daily living, socialisation, motor skills), and subdomain scores (receptive, expressive, written, personal, domestic, community, interpersonal, play and leisure, coping skills, gross motor, and fine motor). Maladaptive behaviour scores to be included where possible.

## Self-report measures (child)

BCE reviewers will be provided with:

### Child Behaviour Checklist (CBCL)

For the self-report version of the CBCL, reviewers will be provided with T-scores and qualitative ranges for the anxious, depressed, social problems, thought problems, oppositional defiant problems, conduct problems, and attention problems subscales.

### Children’s Depression Inventory – Second Edition (CDI-2)

The CDI-2 self-report outcomes will be included where applicable (i.e. when the child is old enough to complete the self-report version). Scaled scores and qualitative ranges for the emotional problems, functional problems, and total scale scores, in addition to the negative mood, negative self-esteem, ineffectiveness, and interpersonal problems subscale scores.

### Spence Children’s Anxiety Scale (SCAS)

For the self-report version of the SCAS, reviewers will be provided with T-scores and qualitative ranges for the generalised anxiety, physical injury fears, panic/agoraphobia, obsessive compulsive, social phobia, and separation anxiety subscales, and the total score.

## Clinical Assessment Measures

### Autism Diagnostic Observation Schedule – Second Edition (ADOS-2)

For review the team will be provided with raw scores for all ADOS-2 items that are added together to make the subdomain scores (i.e. Social Affect [SA], Restricted Repetitive Behaviours [RRB]). The total scores for SA, RRB, Total score (SA + RRB), comparison score, and the ADOS-2 severity score will be provided, as well as the overall ADOS-2 classification.

### 3.2 Cognitive assessment

The review team will be provided with data from the child’s WASI-II, WPPSI-IV, WISC-V, or WAIS-IV (depending on participant age/diagnostic status), which includes standard scores, percentile ranks, 95% confidence intervals, index scores, and qualitative ranges.

### 3.3 Language assessments

The review team will be provided with data from the CELF-5 Screener, and CELF-5, CELF-P2, or PLS-5 (if administered), which includes standard scores, percentile ranks, 95% confidence intervals, domain scores, and qualitative ranges.

## External reports

All external reports for probands and siblings (where applicable) are collected by the MAGNET Project. External reports will be reviewed for BCE if further diagnostic clarification is required following review of the parent report, self-report, and clinical assessment measures collected as part of the MAGNET Protocol. The purpose of the BCE is to be an independent review of diagnosis. It is ideal if BCE assessors do not have knowledge of the external clinician’s diagnosis (blind assessment).

A member of the research team who is not on the BCE review team will therefore remove diagnostic indicators in external reports before the BCE review if these reports are required.

BCE reviewers will use the following from external reports:

- Cognitive Assessments
- Language Assessment
- Behaviour assessments (e.g. DBC, BASC)
- Functional assessments (e.g. ABAS)
- Other (e.g. occupational therapy)
