# Supplementary material for: The Monash Autism-ADHD genetics and neurodevelopment (MAGNET) project design and methodologies: a dimensional approach to understanding neurobiological and genetic aetiology
Source: Mol Autism. 2021 Aug 5;12:55. doi: 10.1186/s13229-021-00457-3 (PMC8340366; doi:10.1186/s13229-021-00457-3)
Supplement: Supplementary file 3 — Additional file 3. Protocol for testing non-verbal children and children with ID. [file 13229_2021_457_MOESM3_ESM.docx]

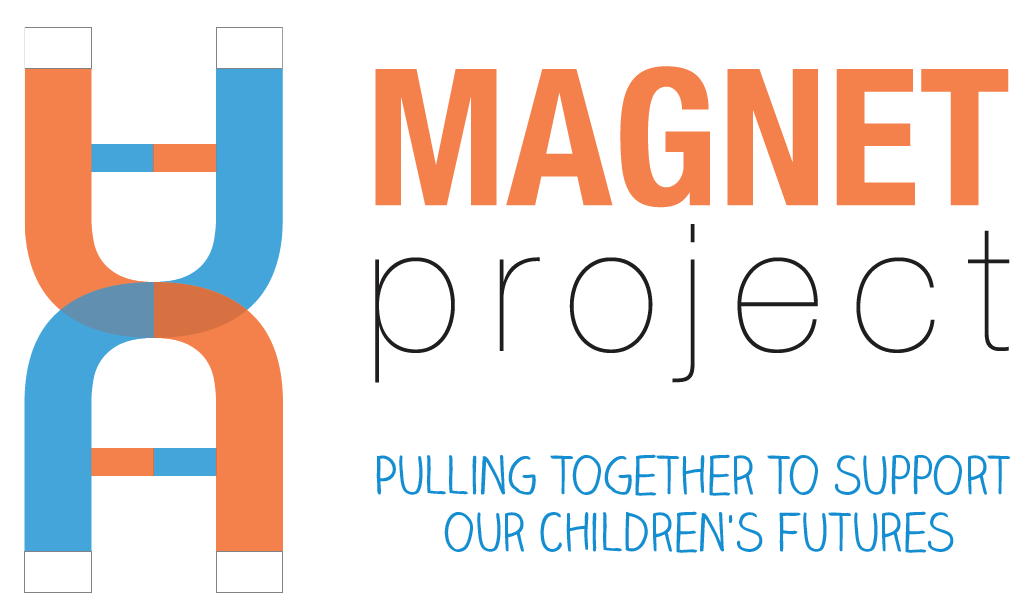


The Monash Autism/ADHD Genetics and Neurodevelopment (MAGNET) Project

Non-verbal Children or Children with an Intellectual Disability Protocol

Minimum data requirements:

- Saliva sample (spit kit or swab)
- Parent questionnaires (completed)
- ADOS (completed)
- Cognitive assessment (attempted): for children under 7 years old attempt WPPSI, then attempt PEP-3
- Speech and Language (attempted)

Research data should be attempted where possible:

- Neurocognitive tasks
- Eye tracking

For families where one of the children is non-verbal or has an intellectual disability the ADOS and cognitive assessment session should be completed first, and be booked in on Mondays or days where our supervising psychologist, speech pathologist, and/or senior members of the research team will be in attendance.
