# Supplementary material for: The Monash Autism-ADHD genetics and neurodevelopment (MAGNET) project design and methodologies: a dimensional approach to understanding neurobiological and genetic aetiology
Source: Mol Autism. 2021 Aug 5;12:55. doi: 10.1186/s13229-021-00457-3 (PMC8340366; doi:10.1186/s13229-021-00457-3)
Supplement: Supplementary file 4 — Additional file 4. Clinical assessments protocol. [file 13229_2021_457_MOESM4_ESM.docx]

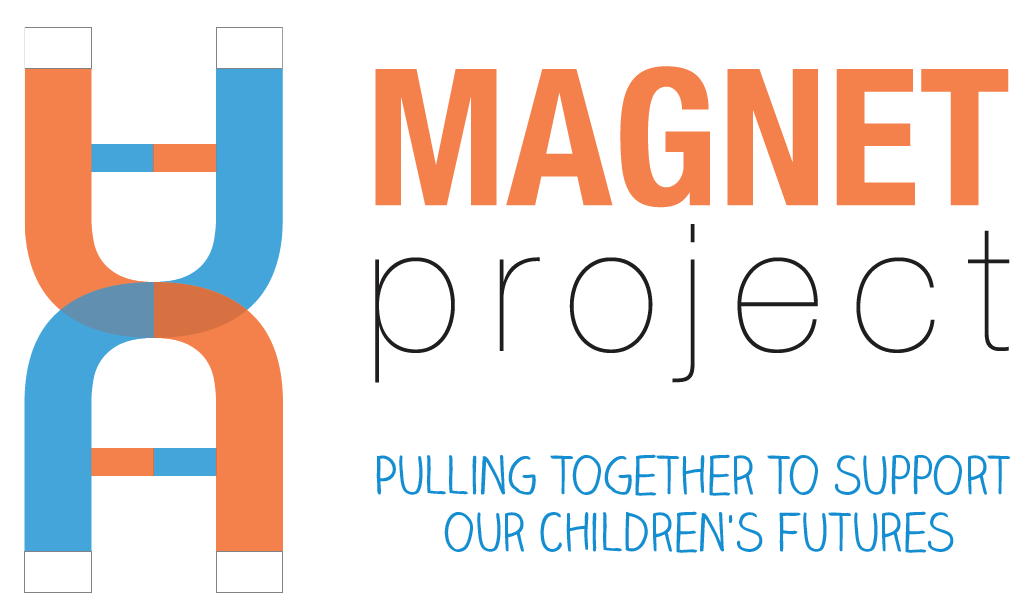


Clinical Assessments SoP

The Monash Autism/ADHD Genetics and Neurodevelopment (MAGNET) Project

Principal investigators

Dr Beth Johnson, Prof Mark Bellgrove

Turner Institute of Brain and Mental Health

Monash University

Date: 14 September 2020

Revision: 2

Confidential

This document is confidential. It may not be transmitted, reproduced, published, or used without prior written authorization.

Statement of Compliance

This document is a protocol for a research project. This study will comply with this protocol, the conditions of the ethics committee approval, and the NHMRC National Statement on ethical Conduct in Human Research (2018).

**Cognitive Assessments**

Standardised measures of cognitive function (WASI-II/WPPSI-IV/WISC-V/WAIS-IV) are administered in line with the administration guidelines in the respective Wechsler manuals. Children entering the study as typically developing, with no current concerns raised about their developmental trajectory, will be administered a WASI-II (6 years 0 months and older) or a WPPSI-IV (4 years 0 months – 5 years 11 months). Children with a current neurodevelopmental diagnosis, or who have had concerns raised by a parent, school or treating team will be administered a WPPSI-IV, WISC-V or WAIS-IV, depending on their age. All siblings, irrespective of case-control status are to be administered a WPPSI-IV, WISC-V, or WAIS-IV, dependent on age. Clinical children and their siblings who are: 4 years 0 months – 5 years 11 months will be administered the WPPSI-IV; 6 years 0 months – 15 years 11 months will be administered the WISC-V; or 16 years 0 months and older are to be administered the WAIS-IV. Item level responses, scaled scores, index scores, and percentile ranks will be entered into the MAGNET Project REDCap database.

**The Vineland Adaptive Behaviour Scale – Third Edition (Vineland-3)**

The Vineland Adaptive Behaviour Scale – Third Edition – Parent/Caregiver Form (Vineland-3) is a measure of adaptive behaviour and is intended for individuals aged 0 to 90 years of age (Sparrow, Cicchetti, & Saulnier, 2016). Adaptive behaviour is conceptualised as how well individuals are able to perform activities required for self-sufficiency and success in their day-to-day lives. The Vineland-3 is divided into subdomains, which are socialisation, communication, daily living, and motor skills (if the child is nine years of age or less). The socialisation subdomain captures the individuals ‘play and leisure’ skills, ability to manage ‘interpersonal relationships’, and their ‘coping skills’. The communication subdomain covers the child’s expressive, receptive and written communication skills. The daily living subdomain explores personal, domestic, and community skills. The motor skills subdomain is intended for children from birth to nine years of age and assesses their fine and gross motor skills compared to other children their age. The parent/caregiver for every child (control and clinical) participating in the study completes a Vineland-3 online, which takes 30 and 60 minutes. The Vineland-3 is administered and scored online. Item level responses, subdomain scores, percentile ranks, confidence intervals, and the overall adaptive behaviour score (Adaptive Behaviour Composite), which summarises the child’s adaptive behaviour overall, will be entered into the MAGNET Project database.

**Autism Diagnostic Observation Schedule – Second Edition (ADOS-2)**

The ADOS-2 (Lord et al., 2012) is a semi-structured play-based measure that assesses the communicative, social and repetitive behaviours associated with ASD. It typically takes 40 to 60 minutes and can be administered to individuals aged 12 months to adulthood. The tool comprises a number of planned interactions and activities between child and administrator which provide opportunities for target behaviours to be observed and then coded. The ADOS-2 is to be administered in line with administration guidelines from the manual. Four of the ADOS-2 modules are utilised in the MAGNET Project (Module 1, 2, 3 and 4). The most appropriate module is chosen based on the participants chronological age and level of expressive language. All ADOS-2 assessments are video recorded to allow for scoring by a second-rater, with final scores determined through consensus coding. ADOS-2 assessments are coded by members of the research team with ADOS-2 for Research training and research reliable coding. One rater is blinded to the diagnostic status of the participant during coding of ADOS-2 assessments. Scores are calculated for Social Affect (SA) and Restricted, Repetitive and Stereotyped Behaviours (RRB) subdomains, as well as SA + RRB total, and ADOS-2 comparison scores. The ADOS-2’s diagnostic algorithm includes cut-offs which classify individuals into either Autism, Autism Spectrum, or Non-Spectrum categories. Item level responses, subdomain scores, SA + RRB total, ADOS-2 comparison score, and the ADOS-2 classification are entered into the MAGNET Project REDCap database.

**The Developmental, Dimensional and Diagnostic Interview (3di)**

The 3di (Skuse et al., 2004) is a standardised, dimensional, computer-based, diagnostic interview designed to assist with the diagnosis of ASD. In line with a dimensional conceptualisation of autism, the 3di was developed to measure continuously distributed traits in the general population and is appropriate for use with typically developing as well as clinical populations. The 3di contains questions designed to assist in the identification of ASD, including questions on language development and early symptomatology. The 3di has excellent criterion validity when compared with the Autism Diagnostic Interview, and discriminates between ASD cases and non-spectrum cases with good sensitivity = 1.0 and specificity > 0.97 estimates (Skuse et al., 2004).

**The Development and Wellbeing Assessment (DAWBA)**

The DAWBA is a parent-completed questionnaire designed to measure child and adolescent psychopathology (Robert Goodman, Ford, Richards, Gatward, & Meltzer, 2000). The DAWBA’s section on Attention and Activity is used in conjunction with the Conners Rating Scale – Revised (Conners, Sitarenios, Parker, & Epstein, 1998) as a key measure for confirmation of ADHD diagnoses through a Best Clinical Estimate (BCE) review process (see BCE SoP). As recommended by the authors of the measure, the DAWBA is completed by parents online for ease of administration and to ensure skip-rules are accurately adhered to. Items are divided into sections, which include social aptitudes, friendships, development, separation anxiety, specific phobias, social phobias, panic attacks and agoraphobia, post-traumatic stress, compulsions and obsessions, generalised anxiety, depression, attention and activity, awkward and troublesome behaviour, tics, and dieting, weight and body shape.

**Clinical Evaluation of Language Fundamentals – Screening Test (CELF-5 Screener)**

The CELF-5 Screener is a standardised language screening tool that identifies children (aged 5 to 21years) in need of comprehensive language assessment using the most discriminating items from CELF- 5 diagnostic test. The CELF-5 Screener is to be administered in line with administration guidelines from the manual. Items are divided into subtests which include Word Structure, Word Classes, Following Directions, Recalling Sentences, Sentence Assembly and Semantic Relationship. The sum of the total number of correct items contributes to the overall score, which is then compared to a criterion score appropriate to the child's age. Children whose total scores are at, or below, their age-referenced criterion scores are referred for a comprehensive language evaluation. To ensure the dimensionality of language difficulties are captured, children whose score 2 points above the criterion and/or who passed criterion but showed difficulty in one or any of the subtests, are also administered a comprehensive language test. Administration time for the CELF-5 Screener ranges from 10 to 15 minutes. Item level responses and the criterion score will be entered into the MAGNET Project REDCap database.

**Clinical Evaluation of Language Fundamentals - Fifth Edition (CELF-5)**

The CELF-5 (Wiig, Semel, & Secord, 2013) is a comprehensive language test designed to assess, diagnose and identify language strengths and weaknesses in individuals aged 5 to 21 years. The CELF-5 is to be administered in line with administration guidelines from the manual. Children complete core subtests from the CELF-5 relevant to their age range. Children aged 5 to 8 years old complete sentence comprehension, linguistic concepts, linguistic concepts, word structure, word classes, following directions, formulated sentences, recalling sentences, and understanding spoken paragraphs, and children 9 years and older complete word classes, following directions, formulated sentences, recalling sentences, understanding spoken paragraphs, word definitions, sentence assembly, and semantic relationships. Each subtest is age-specific and includes differential starting points. A parent/caregiver will also complete the Social Pragmatics profile for all children completing a CELF-5, which evaluates the individual’s verbal and non-verbal pragmatic language skills using a checklist of 50 statements. Raw scores, scaled scores, 95% confidence intervals, and percentile ranks for subscales, and standard scores, 95% confidence intervals, and percentile ranks for core language and index scores will be entered into the MAGNET Project REDCap database.

**Clinical Evaluation of Language Fundamentals – Preschool Edition (CELF-P2)**

The CELF- P2 (Wiig, Secord, & Semel, 2004) is a comprehensive language assessment designed to identify language strengths and weaknesses in preschool children aged 3 to 6 years 11 months. The CELF-P2 is to be administered in line with administration guidelines from the manual. The CELF – P2 subtests administered include sentence structure, word structure, expressive vocabulary, following directions, basic concepts, recalling sentences, and word classes. Children who are 4 years 0 months to 4 years 11 months of age will undertake this assessment. However, it is expected that there will be participants between 5 and 6 years 11 months who are presenting with significant language difficulties (e.g. Intellectual Disability and Language Disorders). In these instances, the CELF-P2 will be administered instead of the CELF-5 as this assessment tool will be more appropriate and increase the likelihood of completion. The overall administration time for CELF-P2 ranges from 20 to 40 minutes. Raw scores, scaled scores, 95% confidence intervals, and percentile ranks for subscales, and standard scores, 95% confidence intervals, and percentile ranks for core language and index scores will be entered into the MAGNET Project REDCap database.

**Preschool Language Scales – Fifth Edition (PLS-5)**

The PLS-5 (Zimmerman, Steiner, & Pond, 2011) is a measure of language, incorporating information from clinician observation, direct measurement and parent report. The PLS-5 is to be administered in line with administration guidelines from the manual. PLS-5 is administered to younger minimally verbal children in the age range of birth to 7 years 11 months and consists of two standard scales: Auditory comprehension and Expressive communication. Total administration time for PLS-5 ranges from 35 to 60 minutes. This assessment will be administered should a child present with limited language skills (e.g. non-verbal) and is unable to complete the CELF-P2. Raw scores, scaled scores, 95% confidence intervals, and percentile ranks for the subscales and total language score, as well as discrepancy comparison values, will be entered into the MAGNET Project REDCap database.
