# Supplementary material for: The Monash Autism-ADHD genetics and neurodevelopment (MAGNET) project design and methodologies: a dimensional approach to understanding neurobiological and genetic aetiology
Source: Mol Autism. 2021 Aug 5;12:55. doi: 10.1186/s13229-021-00457-3 (PMC8340366; doi:10.1186/s13229-021-00457-3)
Supplement: Supplementary file 5 — Additional file 5. Research visit and neurocognitive testing protocol summary. [file 13229_2021_457_MOESM5_ESM.docx]

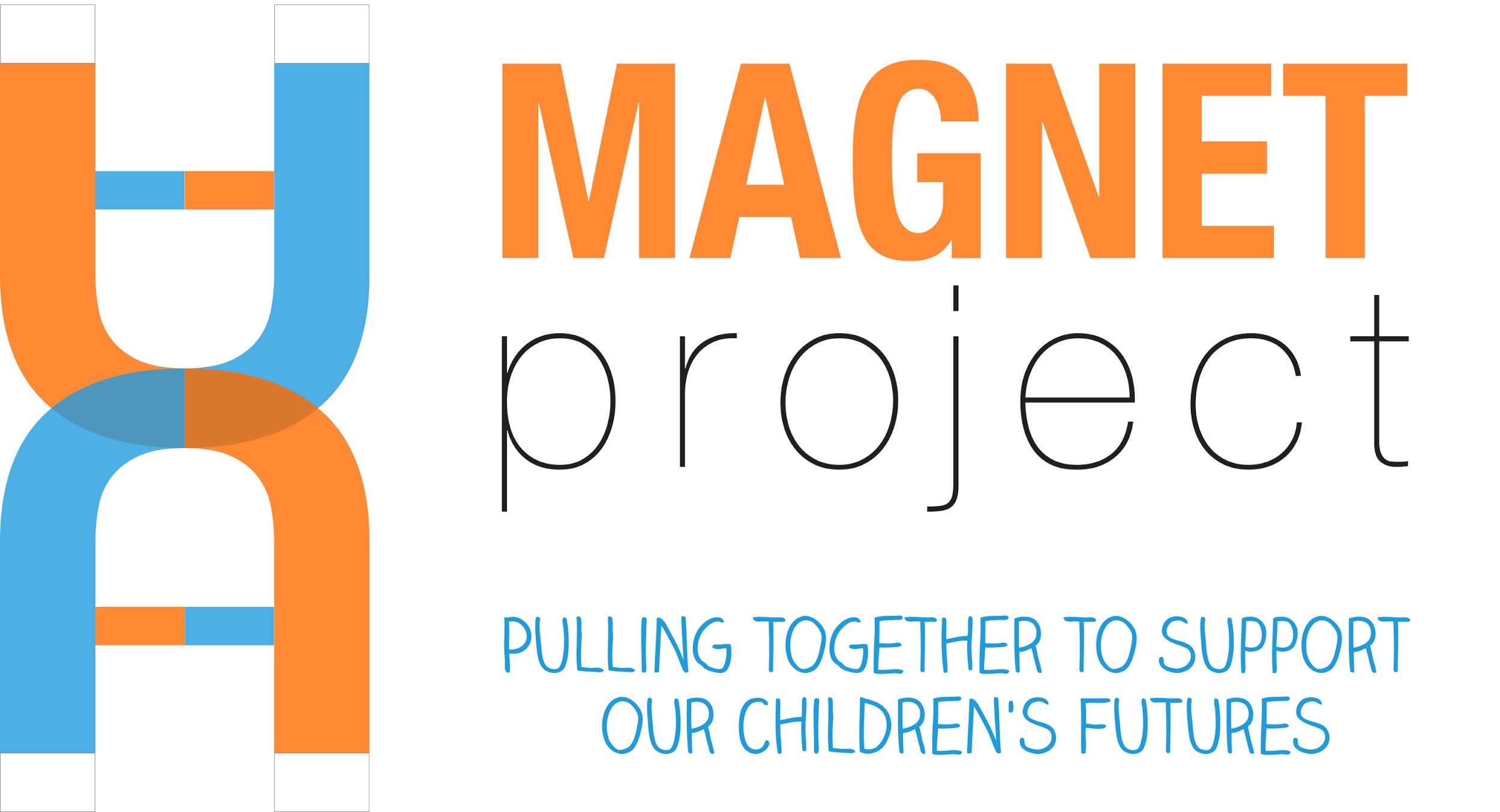


Research Visit and Neurocognitive Testing SoP

The Monash Autism/ADHD Genetics and Neurodevelopment (MAGNET) Project

**Principal investigators**

Dr Beth Johnson, Prof Mark Bellgrove

Turner Institute of Brain and Mental Health

Monash University

Date: July 2020

Revision: 5

**Confidential**

This document is confidential. It may not be transmitted, reproduced, published, or used without prior written authorization.

**Statement of Compliance**

This document is a protocol for a research project. This study will comply with this protocol, the conditions of the ethics committee approval, and the NHMRC National Statement on ethical Conduct in Human Research (2018).

Contents

[STAFF AND STUDENT REQUIREMENTS 4](#_Toc52442363)

[REQUIREMENTS BEFORE EACH SESSION 4](#_Toc52442364)

[Clothing 4](#_Toc52442365)

[Staff illness 4](#_Toc52442366)

[SCREENING PARTICIPANTS FOR COVID-19 AND OTHER ILLNESS PRIOR TO TESTING 4](#_Toc52442367)

[Case Report Forms (CRF) 5](#_Toc52442368)

[SITE-SPECIFIC TESTING 5](#_Toc52442369)

[Overview of Testing at Monash University 5](#_Toc52442370)

[OVERVIEW OF TESTING IN SCHOOLS 6](#_Toc52442371)

[Administration of Standardised Clinical Assessments 7](#_Toc52442372)

[SITE SETUP PROCEDURE 7](#_Toc52442373)

[clean and disinfect test spaces and equipment on arrival 7](#_Toc52442374)

[Set up checklist 7](#_Toc52442375)

[PARTICIPANT TESTING 8](#_Toc52442376)

[MEDICATION WITHDRAWAL 8](#_Toc52442377)

[EQUIPMENT SET-UP 8](#_Toc52442378)

[Eye Tracking - Eyelink 1000 Eye Tracker 8](#_Toc52442379)

[VISIT 1 – NEUROCOGNITIVE TASK ADMINISTRATION 9](#_Toc52442380)

[touchscreen tasks and Celf-5 screener 9](#_Toc52442381)

[Spatial Working Memory – Find the Phone 9](#_Toc52442382)

[Karolinska Directed Emotional Faces (KDEF) 9](#_Toc52442383)

[Reading the Mind in the Eyes Task (RMET) - Child 10](#_Toc52442384)

[New Cambridge Gambling Task 10](#_Toc52442385)

[New Reversal Learning 11](#_Toc52442386)

[Continuous False Belief – Sandbox Task 11](#_Toc52442387)

[CELF-5 Screening tool 12](#_Toc52442388)

[STOP SIGNAL, ANT AND EYE TRACKING tasks 12](#_Toc52442389)

[Tasks 10-13: EyeLink 12](#_Toc52442390)

[Visually Guided Saccade 12](#_Toc52442391)

[Antisaccade Paradigm 12](#_Toc52442392)

[Smooth Pursuit 1: SPEM 12](#_Toc52442393)

[Smooth Pursuit 2: Step Ramp 12](#_Toc52442394)

[Go/No-Go 12](#_Toc52442395)

[Face Recognition 13](#_Toc52442396)

[Stop Signal Task 14](#_Toc52442397)

[Tasks 8, 9, 17 & 18 15](#_Toc52442398)

[Spence Children’s Anxiety Scale 15](#_Toc52442399)

[Physical Measurements 15](#_Toc52442400)

[Beighton’s Hypermobility Scale 15](#_Toc52442401)

[Saliva Collection 15](#_Toc52442402)

[VISIT 2 – STANDARDISED CLINICAL ASSESSMENTS 16](#_Toc52442403)

[Tasks 19-20: Cognitive assessment, ADOS, Speech & Language Assessment & Vineland 3 16](#_Toc52442404)

[Cognitive assessment summary 16](#_Toc52442405)

[Speech & Language assessment summary 16](#_Toc52442406)

[Administration and Infection Control 18](#_Toc52442407)

[ADOS-2 18](#_Toc52442408)

[Arrangement of the room for modules 1 & 2 18](#_Toc52442409)

[Arrangement of the room for modules 3 & 4 19](#_Toc52442410)

[Clean up at the conclusion of the assessment 19](#_Toc52442411)

[ados video data back up 19](#_Toc52442412)

[Return to Home – Important for MAGNET team members: 19](#_Toc52442413)

# STAFF AND STUDENT REQUIREMENTS

Staff and students are to meet the following requirements before commencing testing.

- All staff and doctoral students must be GCP trained to work on the project
- All staff and students must have a valid Working With Children Check
- All staff and students must be trained and inducted into this procedure
- All staff and students must complete local area inductions. There are separate inductions for general facility usage and ocular motor equipment usage
- All staff and students collecting saliva must be trained to do so
- All staff and students must be trained by the supervising psychologist, or have other relevant training or qualifications before administering the WISC, WPPSI or WASI
- When working with children, at least two staff or students must be present during the testing session
- All staff and students should have Mental Health First Aid training
- All staff and students should have a Hepatitis B immunisation before starting work

# REQUIREMENTS BEFORE EACH SESSION

## Clothing

For the purposes of infection control, staff and students (herein referred to as research staff) must wear easy to wash, easy to wear clothing (e.g. scrubs) including shoes. You will need to have spare clothing at end of session/getting home. Avoid jewellery or other items that a child may pull or grab at, or be a potential source of cross contamination.

## Staff illness

If you are unwell and experiencing symptoms of COVID-19 (however mild), you are directed not to come to work, you must get tested and only come to campus when you have recovered and a negative test result; and/or have concluded appropriate quarantine. If staff members become unwell either before or during the session (see Signs of COVID-19), they must immediately notify Dr Beth Johnson and any other staff members who are rostered on the same day to arrange an alternative staff member to attend the session.

# SCREENING PARTICIPANTS FOR COVID-19 AND OTHER ILLNESS PRIOR TO TESTING

During all screening conversations, the message is reinforced to parents that if any infection is reported or observed, testing will not proceed. This is also embedded within the ‘emails to parents’ and registration, and SMS notifications/reminders.

Signs of COVID-19 include:

- Fever (>37.5)
- Cough
- Sore throat
- Shortness of breath
- Chills/body aches, headache
- Runny nose
- Muscle pain
- Diarrhoea

Only participants can attend session. Parents and siblings that are participating in the study, and dependents (young children) can be present. Parents will need to find alternative arrangements for other children.

Inform carer/parent if there is a reported infection at the testing site for the MAGNET Project. Confirm with participating family key contact numbers/emails so if there is a reported cluster the family can be informed so they can get tested.

If participants meet any of these criteria at the time of testing, the session must be discontinued. The researcher is required to provide a surgical mask to the participant and instruct them on how to use it, recommend the participant be tested for COVID-19, and request they leave the premises. A list of COVID-19 testing sites must be provided to the participant. The participant must return a negative COVID test before rebooking.

# Case Report Forms (CRF)

Trained research staff on the MAGNET Project will document the administration of tasks using a case report form (CRF) during each research visit. All CRF information is entered into REDCap at the conclusion of each session.

At the research visit, a researcher will fill in a CRF for each participant. This will act as a checklist for the standardised assessments and neurocognitive tasks, where the date, time, and any comments are recorded.

# SITE-SPECIFIC TESTING

## Overview of Testing at Monash University

Eligible families will be invited to attend a research session at Monash University.

Table 1 details the assessments that should be completed in each session based on the child’s case-control status.

The Beighton’s mobility task, growth measurements, the CELF-5 screener, Children’s Depression Inventory, Child Behavior Checklist, and the Spence Children’s Anxiety Scale (SCAS) sections can be completed at any time during the testing sessions, and it is advised that these tasks be used to help break up the session.

The order of administration for the neurocognitive tasks is based on that of the EU-AIMS, and is listed as follows (Table 2). The CRF for testing at Monash University is the same one used when testing in schools.

Table 1. Assessments completed during each visit

| Controls | ASD and ADHD | | |
| --- | --- | --- | --- |
| Visit 1  (4-5 hours) | Visit 1 (unmedicated) (3.5 hours) | Visit 2 (medicated) (3.5 hours) | Visit 3 (medicated) (2 hours) |
| Neurocognitive tasks & Eye tracking | Neurocognitive tasks & Eye tracking | WISC-V / WPPSI-IV / WAIS-IV (medicated) | CELF 5/CELF-P2 / PLS 5 (medicated) |
| CELF Screener | CELF Screener | ADOS (medicated) |  |
| WASI | Saliva | Any missing neurocog tasks |  |
| ADOS | Parent: Vineland | Parent: Questionnaires |  |
| Saliva |  |  |  |
| Parent: Vineland |  |  |  |

Table 2. Summary of assessments completed during Visit 1 (controls) or Visit 2 (ASD/ADHD)

| Task | Program | Age (years) | Time (minutes) |
| --- | --- | --- | --- |
| 1. Spatial working memory | Psytools | All | 10 |
| 1. KDEF | Psytools | 8+ | 10 |
| 1. Reading the Mind in the Eyes | Psytools | 8+ | 10 |
| 1. New Cambridge Gambling task | PsychoPy | All | 5 |
| 1. New reversal learning | Psytools | All | 10 |
| 1. Continuous false belief (sandbox) | Touchscreen laptop | All | 5 |
| 1. CELF-5 Screener | Pen & paper | 5+ | 15 |
| 1. Beighton Mobility Task | Pen & paper/REDCap | All | 2 |
| 1. Visually guided saccade task | EyeLink1000 | All | 5 |
| 1. Anti-saccade | EyeLink1000 | 8+ | 5 |
| 1. Smooth pursuit | EyeLink1000 | All | 5 |
| 1. Smooth pursuit 2 | EyeLink1000 | All | 5 |
| 1. Go/No-Go | ANT | All | 10 |
| 1. Face recognition | ANT | All | 5 |
| 1. Stop signal | STOP-IT | 6+ | 15 |
| 1. Saliva collection | - | All | 10 |
| 1. Growth measurements | Pen & paper/REDCap | All | 2 |
| 1. WISC/WPPSI/WASI/WAIS | Pen & paper/Q-Interactive | All | 60-90 |
| 1. ADOS | Pen & paper | All | 45-60 |
| 1. Vineland | Q-Global | All | 15 - 30 |

## OVERVIEW OF TESTING IN SCHOOLS

All questionnaires must be completed prior to the school visit. This is to ensure no missing data/loss of data occurs. The child will complete all tasks as above at their school, however they are broken into 1.5-hour blocks.

When available, a room will be set aside to complete the Cognitive Assessment and the ADOS-2, as a quiet environment is particularly important during these assessments. The MAGNET team will set up different testing stations in a quiet area of the school. The researcher who administered the task, order of administration, and date and time that the task was completed should be noted in the CRF.

The family may be invited to Monash University research facilities if testing is not able to be completed at the school, or if additional assessments are required.

## Administration of Standardised Clinical Assessments

All research staff must be trained, and supervised by the project’s supervising psychologist or speech pathologist (as appropriate) to administer standardised clinical assessments (i.e. WASI-II, WISC-V, WPPSI-IV, ADOS-2, CELF-5, CELF-P2, PLS-5) which must be completed in a quiet space, and, where possible, with a maximum of two clinical assessments per session. This is to avoid participant fatigue. Standard instructions are found in the assessment manuals.

# SITE SETUP PROCEDURE

## clean and disinfect test spaces and equipment on arrival

Rooms must be cleaned and disinfected by researchers before and after use. The researcher must clean and disinfect all surfaces that will come in contact with other people. You must assume that other researchers have been using the site prior to you.

Clean and disinfect commonly touched surfaces

1. Wash and sanitise your hands prior to cleaning and disinfecting the room. Put on gloves.
2. Clean tables, chairs, door knobs, light switches, phones, and any other surfaces that are touched by hands (e.g. door handles, light switches, filing cabinets) thoroughly using household cleaning product and paper towel. Dispose of paper towel in regular waste bins.
3. Disinfect tables, chairs, door knobs, light switches, sneeze guards, and any surfaces that are touched by hands (e.g. door handles, light switches, filing cabinets) with ethanol, diluted bleach or other disinfectant using paper towel or disinfectant wipes

Consider ALL of the places and surfaces that children touch when undertaking your cleaning. Dispose of paper towel or wipes in regular waste bins (not biohazard). When using paper towel or disposable disinfectant wipes, use the simple rule: ONE WIPE, ONE SITE, ONE DIRECTION, DISCARD

Clean and disinfect computers, peripherals and other electronic equipment

All keyboards, mice, cables, lux meter, etc. must be cleaned and disinfected. If researchers have brought work laptops or other equipment into the facility, this must be cleaned and disinfected before introducing it into the room. Avoid sharing laptops between participants and other researchers. Clean and disinfect all surfaces at the beginning and end of each session.

1. Ensure all equipment is turned off
2. Put on disposable gloves
3. Clean the keyboard, mouse, cables and peripheral cables, chin rests and other surfaces regularly touched on the host PC desk. This is done by spraying household cleaning detergent into a soft cloth (e.g. chux), then gently rub over all the surfaces. Dispose of the cloth wipe at the end of cleaning.
4. Disinfect the keyboard, mouse, cables and peripheral cables etc. as above with disinfectant wipes, or ethanol or viraclean sprayed onto a soft cloth.
5. Dispose of towels or wipes in regular waste bins.

When using paper towel or disposable disinfectant wipes, use the simple rule: ONE WIPE, ONE SITE, ONE DIRECTION, DISCARD

Remove gloves and place in biohazard bin. Wash your hands with soap and water for 20 seconds.

# PARTICIPANT TESTING

## MEDICATION WITHDRAWAL

Wherever possible, children taking medication must withdraw from medication for 48-72 hours prior to testing for Visit 1 (Neurocognitive testing). This is discussed with parents during the initial phone screening and booking. A summary of medication withdrawal is below (Table 3). Research staff will need to confirm current medications and medication withdrawal on the day of testing.

Table 3. Medications for withdrawal or maintenance during MAGNET Project protocol.

| Withdraw | Do not withdraw |
| --- | --- |
| Stimulants   - Methylphenidate (Ritalin, Concerta) - Lisdexamfetamine (Vyvanse) - Dexamfetamine | Guanfacine (Intuniv)  Antipsychotics   - Risperidone (Risperdal) - Aripiprazole (Abilify)   Atomoxetine (Strattera, Atomerra)  Clonidine (Catapress) |

# EQUIPMENT SET-UP

### Eye Tracking - Eyelink 1000 Eye Tracker

#### Setup chair and headrest

Participant (and researcher) ergonomics is important. The participant must remain very still during the eye tracking tasks, and so it is important that they are positioned comfortably. The participant should be positioned so that their back is straight and their chin is resting in the headrest, without a slouch. The height of the chair and headrest can be altered, and researchers should be trained to a point that they feel confident in altering the ergonomic set up of the participant as required.

#### Position of Eyelink Camera and Screen

It is imperative that the distance of the monitor, and distance of the illuminator to the chin rest are measured every time, in case items in the room have been moved between sessions. The Eyelink illuminator camera must be positioned 40-55cm away from the centre of the headrest. The middle of the Screen must be 84cm away from the centre of the headrest.

#### Starting Eyelink

1. Turn on Eyelink computer.
2. Tilt the camera so that the participant’s eyes are centred at the widest part of the display on the Eyelink Screen, and so that the face is not tilted.
   1. The vertical dotted line should line up along the centre of the face.
3. Focus the eye via the camera
4. Move to the Eyelink computer to focus the pupil and cornea for each eye.
   1. Pupil threshold should be between 75-110.
   2. Cornea threshold should be between 200-230, although 210-220 is ideal.
5. If a participant is wearing glasses, select “Use Search Limits” and red circles will appear which limit the area of the camera’s focus and avoid glare or reflection from the glasses. **The sample rate should be 500 Hz. The EyeLink changes this back to 1000 Hz frequently, so make sure to check that this is where it should be.**

#### Lux recording

The amount of light in the room is measured using a luxometer. This value must be written on the CRF. Position the luxometer in the next to the keyboard on the right side of the table the participant sits at to complete eye tracking.

# VISIT 1 – NEUROCOGNITIVE TASK ADMINISTRATION

There are tasks that can be administered anywhere and should be completed whenever a researcher has time in their testing session – however, for the purpose of consistency, certain tasks will be grouped together with others to ensure no data is missing.

## Neurocognitive tasks and Celf-5 screener

### Spatial Working Memory – Find the Phone

The Spatial Working Memory task is run via Psytools. The participant must find the phone that is ringing, remembering which phones have rung before.

- Participants uses the touchscreen. Instructions are from Psytools.


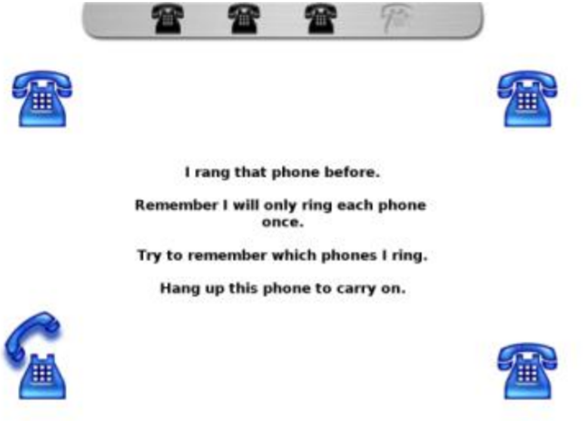


Instructions / Practice / Test:

1. Instructions appear on the screen: read these out to the child.
2. When the test begins, it is not necessary to read the speech box each time it appears

- If the child doesn’t seem to understand on the first page, use the practice to explain again.

### Karolinska Directed Emotional Faces (KDEF)

The KDEF is run via Psytools. The participant must select which emotion they think best matches the picture of the face.

- This task is only administered for ages 8+, or if the participant’s reading ability is suitable.
- Instructions are from Psytools.


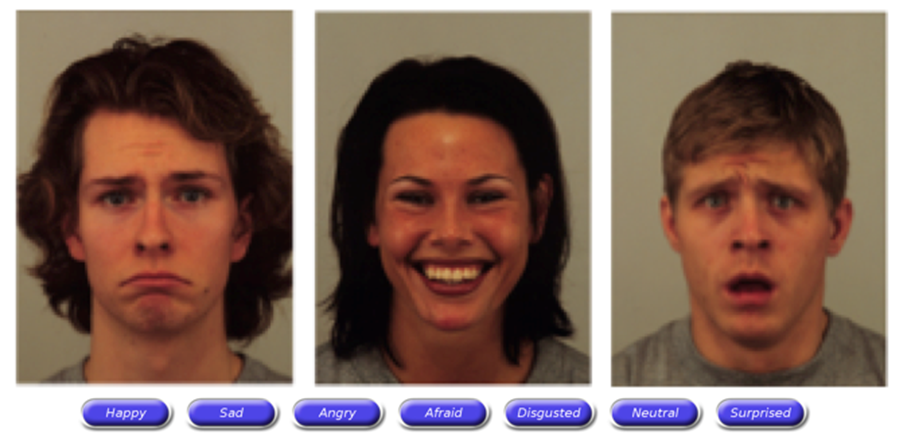


Instructions / Practice / Test:

- Instructions appear on the screen: read these out to the child. Ask the participant to read the emotions aloud to you to ensure they understand them.
  - If the child seems unsure about any emotions, then ask the child to show you their faces for each emotion – if the child can’t, show them what it looks like, then ask them to complete the task.

### Reading the Mind in the Eyes Task (RMET) - Child

The RMET is run via Psytools.

- The participant must select what they think the person is thinking or feeling.
- This task is only administered for ages 8+, or if the participant’s reading ability is suitable.
- Choose task relevant to participants age (‘Child’ – children under 13; ‘Adolescent’ – children under 19)
- Instructions are from Psytools.


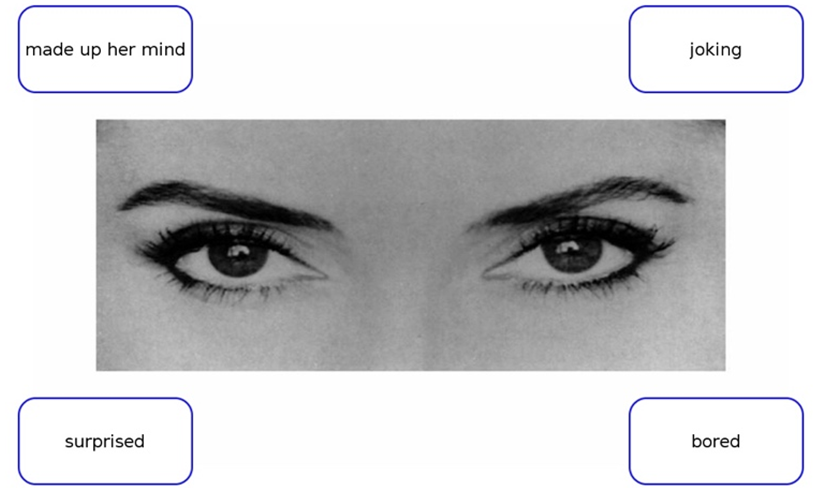


Instructions / Practice / Test:

- Instructions appear on the screen: read these out to the child.
- The emotions change with each picture: start by reading these out whilst pointing to them, though the researcher may cease this if it is clear that the participant is able to read and understand them.
  - Tell the participant that they can ask the researcher if they are unsure of the word meaning.

### New Cambridge Gambling Task

The New Cambridge Gambling Task explores decision making and risk taking. Children must choose what colour they think the spinning pointer will land on, and place a “guess” using “tokens”.

- It should be noted that some children may not understand the concept of this task, especially those under 6 years: ensure to note this is noted in the CRF if this is the case.
- The researcher must word the instructions as a “guess” rather than “bet”, and “tokens” rather than “chips”.


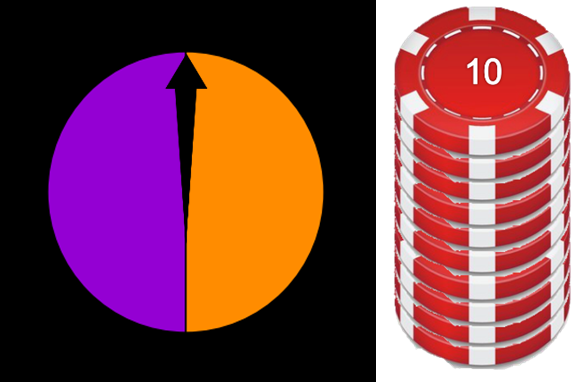


Instructions / Practice / Test:

- Instructions appear on the screen: read these out to the participant.
- There are two conditions: Win/Lose and Keep/Double. When that condition appears, read the instructions to the participant. Don’t mention the other condition until the child gets to it.

### New Reversal Learning

The New Reversal Learning task is run on Psytools and explores the participant’s response following positive and negative feedback.

- The participant must select either the yellow or blue pattern, where they are told that one is correct more often than the other, and they are given positive or negative feedback.
- Instructions are from Psytools.

Instructions / Practice / Test:

- Instructions appear on the screen: read these aloud to the participant – you may need to change the wording slightly for younger children.
  - If the child doesn’t seem to understand, run through the first five trials and explain again.


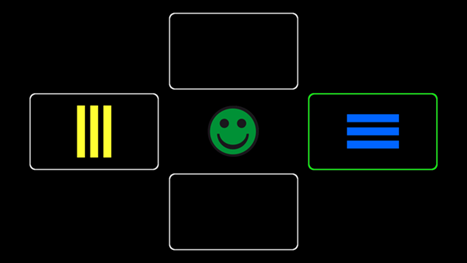


Positive feedback


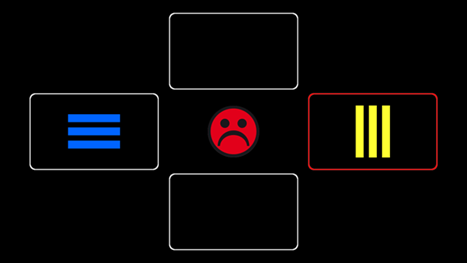


Negative feedback

### Continuous False Belief – Sandbox Task

The Sandbox Task involves reading the story to the participant: the story is about a girl and her father who are planting flower bulbs.

- While the father is absent, the girl moves the flower bulb to a different location (AB – False Belief Condition) or buries a stone in a different location (BA – True Belief Condition).
- The participant is then asked to show where the father thinks the flower bulb is.
- Both conditions are administered, though the order of administration is counterbalanced across participants.
- The task is completed on the touch screen computer.

### CELF-5 Screening tool

- For all children aged 5 – 21 years
- 5 - 8 year old’s complete version 1
- 9 - 18 year old’s complete version 2
- Takes 10-15 minutes to complete
- To be administered by members of the research team with appropriate training.

## STOP SIGNAL, ANT AND EYE TRACKING tasks

### Tasks 9 - 12: EyeLink

1. Setup chair and headrest
2. Turn on Eyelink computer.
3. Tilt the camera so that the participant’s eyes are centred at the widest part of the display on the Eyelink Screen, and so that the face is not tilted.
   1. The vertical dotted line should line up along the centre of the face.
4. Focus the eye via the camera:
5. Move to the Eyelink computer to focus the pupil and cornea for each eye.
   1. Pupil threshold should be between 75-110.
   2. Cornea threshold should be between 200-230, although 210-220 is ideal.
6. If a participant is wearing glasses, select “Use Search Limits” and red circles will appear which limit the area of the camera’s focus and avoid glare or reflection from the glasses.
7. The sample rate should be 500 Hz. **The EyeLink changes this back to 1000 Hz frequently, so make sure to check that this is where it should be.**

### Visually Guided Saccade

Instructions / Practice /Test:

“All you need to do is look in the middle of each cross.”

### Antisaccade Paradigm

This task requires the participant to inhibit a saccade and is only administered for ages 8+

Instructions / Practice /Test:

1. “In this task, a cross will appear on either the right hand side or left hand side of the screen. Your job is look to the mirror opposite side of the screen to the cross.
2. Here is an example;
3. Start with your eyes focused on this central square
4. A cross will appear, but don’t look at it! Instead look to the same spot on the opposite side of the screen. In this case this would be to here [point to where it would be]
5. Then, when the next dot appears in the centre, you can look back there. Let’s have a go.”

### Smooth Pursuit 1: SPEM

Instructions / Practice /Test:

- “Watch the ball as it moves across the screen.”

### Smooth Pursuit 2: Step Ramp

Instructions / Practice /Test:

- “Watch the ball as it moves across the screen.”

### Go/No-Go

The Go/No-Go Task is administered via the ANT program.

- The participant will be shown either a Green ‘Go’ Signal or a Red ‘No-Go’ Signal, and must press the space bar as quickly as they can when the Go signal is presented.
- Instructions are from the ANT Manual.
- The participant will do this twice – the first block has equal green and red men (equal) and the second has more green men (biased)
-
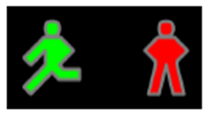
Ensure the participant is using their dominant hand.

Instructions:

1. “Here is a green man and a red man.
2. The green man is the go man and the red man is the stop man.
3. When you see the green man, press the space bar
4. When you see the red man, the stop man, DON’T press the spacebar”

Practice:

1. “Let’s practice!
2. Hold your finger on the key.
3. Try to respond as fast as you can without making mistakes.”
   - The practice task can be run twice if necessary.

Test:

1. “Now the real test comes.
2. We will play this game twice.
3. Hold your finger on the key.
4. Are you ready?”
5. Run the second run through (biased)

### Face Recognition

The Face Recognition Task is administered via the ANT program.

- The participant will be shown a picture of one face which disappears, and then they must decide whether that face is present in a set of four pictures.
- The participant will do this once.
- Instructions are from the ANT Manual.
- The response keys differ depending on the participant’s handedness.

| Right-handed  right key = yes  left key = no | Left-handed  left key = yes  right key = no |
| --- | --- |


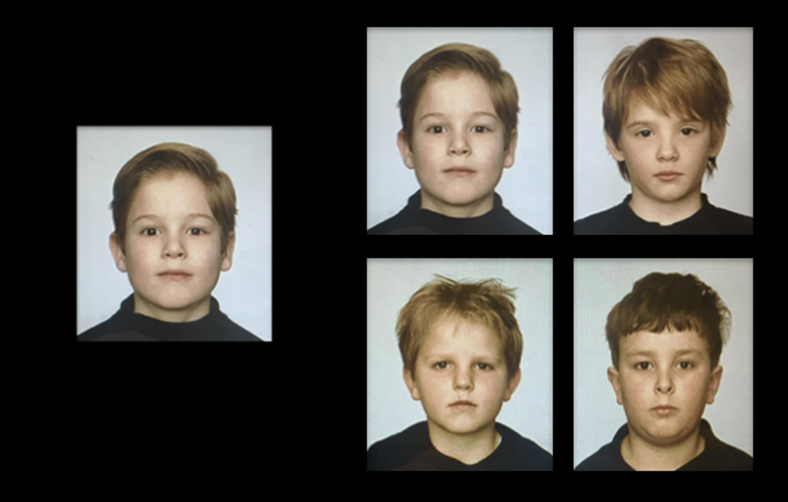


Instructions:

1. “We would like to know how good you can recognize faces and how quick you are.
2. Each time you will see a picture of a face, like this one [instruct], you must look at it carefully
3. We will ask you if this face is present in the next picture which always contains four faces, for example like this picture [instruct].
4. You should press the yes-key when that face is present and the no-key when it is absent. When you know the answer, you should press the key as fast as possible.
5. The yes key is this key (indicate to child) and the no key is this key (indicate to child).”

Practice:

1. “Let’s practice!
2. Put index finger and middle finger on the response keys.
3. Respond as fast and as accurately as possible.”

The practice task can be run twice if necessary.

Test:

1. “Now the real test comes.
2. Respond as fast as you can without making mistakes.
3. Hold your fingers on the keys.”

### Stop Signal Task

The Stop Signal Task explores reaction time and inhibition and is only administered for ages 6+.

1. The participant is shown a circle or a square on the screen responds by pressing a key.
2. When a sound is played along with the stimuli, participants must not press the key, and rather inhibit their response.
3. Each block is about 3 minutes long and there are three blocks. Participants will use the keyboard.

Instructions / Practice / Test:

Instructions appear on the screen: however, they are complex and not suitable for children. Read these aloud to the participant.

1. In this game, there will be a square or a circle that pops up on the screen
2. When you see the square, press the z key
3. When you see the circle, press the / key
4. Your job is to press the ‘z’ or ‘/’ key as soon as you see one, okay?
5. However, this game is tricky – sometimes when a square or circle pops up a beep sounds
6. When that beep sounds, we want you to NOT press any key and wait for the next picture, okay?
   - Ensure child understands: if they don’t, run through the practice with the instructions again and correct them when they get it wrong.
7. Do the practice
8. Check child is ready and tell them to press a response key to start the real test

| Z key  Left index finger | / key  Right index finger |
| --- | --- |

## Tasks 8, 16 & 17

These tasks are on the CRF and any of the researchers can complete them, excluding saliva which can be completed at any time.

### Physical Measurements

The child’s height, head circumference, and weight must be measured and entered into the CRF.

### Beighton’s Hypermobility Scale

The Beighton’s hypermobility scale measures a child’s degree of hypermobility. Included within the CRF are instructions about how to measure hypermobility. Nine measurements are taken, and the researcher asks the child whether they are able to do each action (e.g. touch the floor with their legs straight), and the researcher must write either Y or N whether the child was able to do this.

### Saliva Collection

Staff and students must be trained to undertake saliva collection. Typically developing children that are Caucasian will be asked to provide a saliva sample for genetic testing. Where possible, this should be completed at the site visit using the ORAgene-DNA saliva kits or ORACollect for Paediatrics DNA swab kits. Prior to testing, label tubes and plastic outer container with the participant ID using permanent marker. See the specific Saliva Collection, Handling and DNA Extraction SOP for additional information.


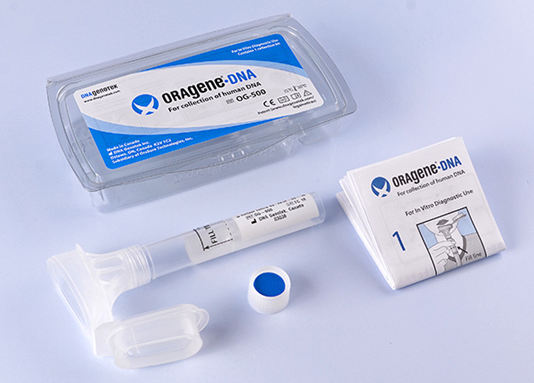

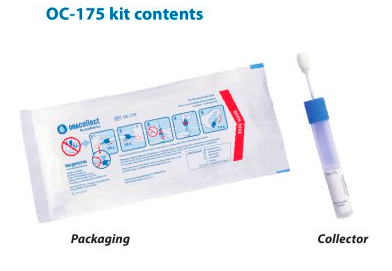


Figure. ORAgene-DNA saliva kit and ORACollect for Paediatrics DNA kits .

# VISIT 2 – STANDARDISED CLINICAL ASSESSMENTS

## Tasks 18-20: Cognitive assessment, ADOS, Speech & Language Assessment & Vineland 3

Summary of how to administer the appropriate cognitive assessment and speech and language assessment tools.

### Cognitive assessment summary

| Assessment | Age range | Administered to |
| --- | --- | --- |
| WISC-V | 6y 0m – 16y 11m | Clinical children, all siblings of clinical children |
| WPPSI-IV | 2y 6m – 7y 7m | All children under the age of 6, children with ID or who are non-verbal or have a very low language level |
| WASI-II | 6y 0m – 90y 11m | Control children |
| WAIS-IV | 16y 0m – 90y 11m | Clinical children, all siblings of clinical children |

Administration of the WISC-V is either completed using Q-interactive, except for the processing speed tasks (symbol search and coding) which are completed using paper and pencil, or using the physical test kits.

WPPSI, WASI and WAIS are all completed using paper and pencil forms.

### Speech & Language assessment summary

| Assessment | Age range | Administered to |
| --- | --- | --- |
| CELF-5 Screening Test | 5 years 0 months -21 years 11 months | All children in the age range. |
| CELF-5 | 5 years 0 months -21 years 11 months | Children that score below criterion on the CELF-5 Screening Test, or within two of criterion; children who are flagged during the ADOS or cognitive assessment; children who have had a previous speech and language assessment and scored below average. |
| CELF-P2 | 3 years 0 months – 6 years 0 months |  |
| PLS 5 | - | Nonverbal or minimally verbal children |

CELF-5 are completed using either Q-interactive or the physical test kits.

CELF-P2 and PLS 5 are completed using paper and pencil forms.

To determine if a child should have a full speech and language assessment (CELF-5, CELF-P2, PLS 5), check the following:

1. Does child have previous ‘atypical’ speech & language assessment (i.e. below average (or more) or weaknesses in given speech areas)
2. Has the child accessed speech therapy/interventions for speech delay, pragmatics, dyspraxia
3. Does child have a current referral for a speech & language assessment from their paediatrician, or if the school has recommended/put them on a waitlist
4. If the CELF-5 screening test comes out either
   1. At or below criterion level or
   2. Within 2 above the criterion level
5. ADOS score:
   1. Module 1: Any score above 0 on any of the following items – A1, A2, A4
   2. Module 2: Any score above 0 on any of the following items – A1, A2, A3, A5
   3. Module 3: Any score above 0 on any of the following items – A1, A2, A7, A8
   4. Module 4: Any score above 0 on any of the following items – A1, A2, A7, A8
6. Clinical judgement (in consultation with the projects supervising psychologist and/or speech pathologist) from working with child i.e. neurocognitive tasks, ADOS, cognitive assessment, CELF-5 screener

#### ADOS-2

Administration of the ADOS-2 is to be completed by a trained member of the research team.

| Module | Age range/population | Administered to |
| --- | --- | --- |
| 1 | 2y 5m +, Pre-verbal/single words | Children with ID or who are non-verbal |
| 2 | Phrase speech (flexible 3-word phrases) | Control or clinical children who use flexible 3-word phrases |
| 3 | Fluent speech | Most participants, control and clinical, will use this module |
| 4 | Adolescent/Adult, fluent speech | Adolescents/adults with fluent speech |

The ADOS-2 administration is recorded on a video camera to be consensus scored by two members of the research team following the assessment.

##### Deciding on an ADOS module

##### At 4 years typically developing children can usually

###### Understanding (receptive)

- answer most questions about daily tasks
- understand most wh-questions (e.g. what, who), including those about a story they have recently heard
- understand some numbers
- show an awareness that some words start or finish with the same sounds.

###### Speaking (expressive)

- use words, such as ‘and’, ‘but’ and ‘because’, to make longer sentences
- describe recent events, such as morning routines
- ask lots of questions
- use personal pronouns (e.g., he/ she, me/you) and negations (e.g., don’t/can’t)
- count to five and name a few colours.

If researchers note that any children they are working with can’t do some of these things, especially count to five or name their favourite colour, make a note on the CRF and discuss with senior members of the research team as to which assessments to administer.

#### Vineland 3

The Vineland 3 is a questionnaire that asks about adaptive behaviour and daily functioning for individuals from birth to 90 years of age. Parents complete a Vineland for every child, usually while they are attending the research visits. NOTE: Occasionally a parent cannot complete the Vineland during the session, in these instances they are sent a link for completion at home.

## Return to Home – Important for MAGNET team members:

It is important to ensure safety of the MAGNET Project team members

- Staff may want to change into clean clothes (remove scrubs).
- When leaving testing site make sure you have washed your hands and arms.
- Maintain wipes in car and be aware of surfaces touched in car (e.g. keys, steering wheel, seatbelt, bag, wallet etc…).
- It is not recommended that you visit other sites on the way home (e.g. visiting the supermarket).
- When entering home make sure hands and arms are washed.
- Highly recommended to change clothing and put work clothes in wash.
