# Supplementary material for: The Monash Autism-ADHD genetics and neurodevelopment (MAGNET) project design and methodologies: a dimensional approach to understanding neurobiological and genetic aetiology
Source: Mol Autism. 2021 Aug 5;12:55. doi: 10.1186/s13229-021-00457-3 (PMC8340366; doi:10.1186/s13229-021-00457-3)
Supplement: Supplementary file 6 — Additional file 6. Oculomotor testing protocol. [file 13229_2021_457_MOESM6_ESM.docx]

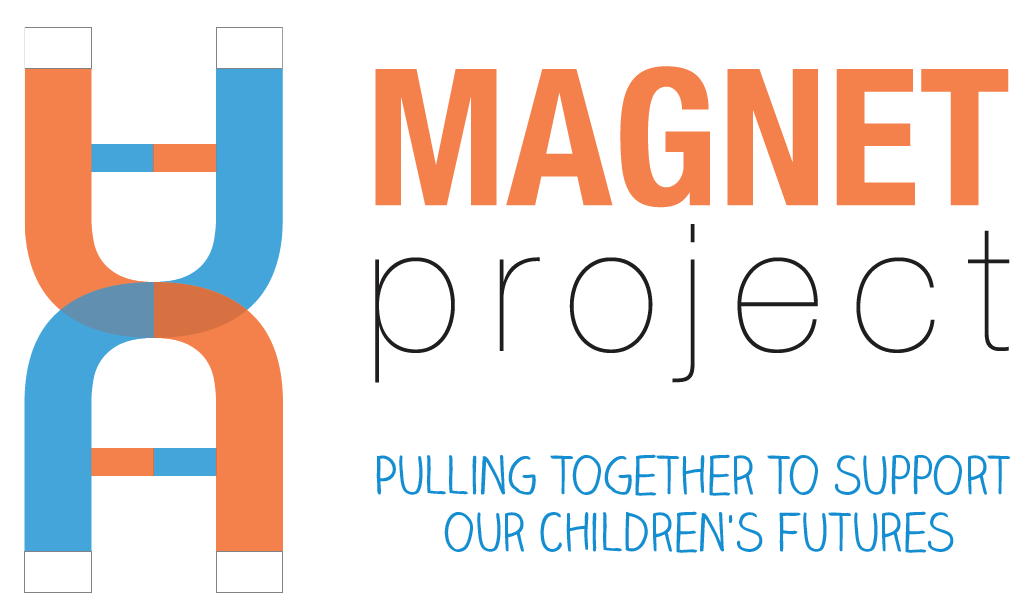


Oculomotor Tasks SoP

The Monash Autism/ADHD Genetics and Neurodevelopment (MAGNET) Project

**Principal investigators**

Dr Beth Johnson, Prof Mark Bellgrove
Turner Institute of Brain and Mental Health
Monash University

Date: January 2020
Revision: 4

**Confidential**This document is confidential. It may not be transmitted, reproduced, published, or used without prior written authorization.

**Statement of Compliance**This document is a protocol for a research project. This study will comply with this protocol, the conditions of the ethics committee approval, and the NHMRC National Statement on ethical Conduct in Human Research (2018).

Contents

[BACKGROUND & EQUIPMENT 3](#_Toc51677196)

[Equipment set up 3](#_Toc51677197)

[Set up checklist 3](#_Toc51677198)

[Setup chair and headrest 3](#_Toc51677199)

[Position of Eyelink Camera and Screen 3](#_Toc51677200)

[Starting Eyelink 3](#_Toc51677201)

[Lux recording 4](#_Toc51677202)

[Ocular motor setup and task administration 4](#_Toc51677203)

[Visually Guided Saccade task 4](#_Toc51677204)

[Antisaccade task 4](#_Toc51677205)

[Smooth Pursuit 1: SPEM 4](#_Toc51677206)

[Smooth Pursuit 2: Step Ramp 4](#_Toc51677207)

# BACKGROUND & EQUIPMENT

This section covers the information about where equipment is stored and how to open the applications used during testing. It also includes login information and setup information for the EyeLink 1000.

#

# Equipment set up

## Set up checklist

Set up Oculomotor lab:

Turn on Host computer

Turn on EyeLink and press enter when prompted to boot up as Eyelink or Windows

Check distances (see Position of eye link camera and screen)

### Setup chair and headrest

Participant (and researcher) ergonomics is important. The participant must remain very still during the eye tracking tasks, and so it is important that they are positioned comfortably. The participant should be positioned so that their back is straight and their chin is resting in the headrest, without a slouch. A cushion is available to ensure that they have appropriate lumbar support. The height of the chair and headrest can be altered, and researchers should be trained to a point that they feel confident in altering the ergonomic set up of the participant as required.

### Position of Eyelink Camera and Screen

The Eyelink camera should be positioned 40-55cm away from the centre of the headrest. The middle of the Screen should be 84cm away from the centre of the headrest. **These distances must be checked every time.**

### Starting Eyelink

1. Turn on Eyelink host and display computers.
2. Tilt the camera so that the participant’s eyes are centred at the widest part of the display on the Eyelink Screen, and so that the face is not tilted.
   1. The vertical dotted line should line up along the centre of the face.
3. Focus the eye via the camera:
4. Move to the Eyelink computer to focus the pupil and cornea for each eye.
   1. Pupil threshold should be between 75-110.
   2. Cornea threshold should be between 200-230, although 210-220 is ideal.
5. If a participant is wearing glasses, select “Use Search Limits” and red circles will appear which limit the area of the camera’s focus and avoid glare or reflection from the glasses. **The sample rate should be 500 Hz. The EyeLink changes this back to 1000 Hz frequently, so make sure to check that this is where it should be.**

### Lux recording

The amount of light in the room is measured using a luxometer. There is one in the ocular motor lab on site, and one in the test kits that is brought to school visits. This value must be written on the CRF. Position the luxometer in the next to the keyboard on the right side of the table the participant sits at to complete eye tracking.

## Ocular motor setup and task administration

###

### Visually Guided Saccade task

Access this task from the desktop

**Instructions / Practice /Test:**

“All you need to do is look in the middle of each cross.”

### Antisaccade task

- Participant performance is very sensitive to task instructions. **Always** use the script.
- Only give 3 prompts per block: If you keep seeing errors on the screen, remind them to look straight across the screen
- Use the pointer stick to show them where to look on the screen during the instruction phase

This task requires the participant to inhibit a saccade and is only administered for **ages 8+**

**Instructions / Practice /Test:**

1. “In this task, a cross will appear on either the right hand side or left hand side of the screen. Your job is look to the mirror opposite side of the screen to the cross.
2. Here is an example;
3. Start with your eyes focused on this central square [*press ‘F’*]
4. A cross will appear, but don’t look at it! Instead look to the same spot on the opposite side of the screen. In this case this would be to here [*press ‘F’*, *point to where it would be]*
5. Then, when the next dot appears in the centre, you can look back there. Let’s have a go. [*press ‘F’*]”

###

### Smooth Pursuit 1: SPEM

**Instructions / Practice /Test:**

“Watch the ball as it moves across the screen.”

###

### Smooth Pursuit 2: Step Ramp

**Instructions / Practice /Test:**

“Watch the ball as it moves across the screen.”
