# Supplementary material for: The Monash Autism-ADHD genetics and neurodevelopment (MAGNET) project design and methodologies: a dimensional approach to understanding neurobiological and genetic aetiology
Source: Mol Autism. 2021 Aug 5;12:55. doi: 10.1186/s13229-021-00457-3 (PMC8340366; doi:10.1186/s13229-021-00457-3)
Supplement: Supplementary file 7 — Additional file 7. Saliva collection protocol. [file 13229_2021_457_MOESM7_ESM.docx]

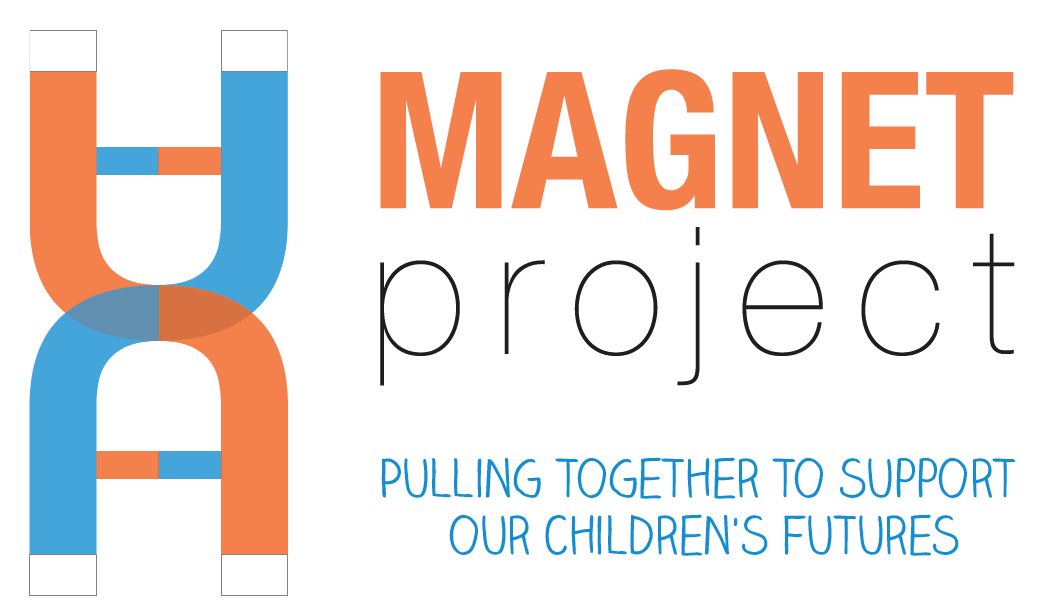


Saliva Collection, Handling, and DNA Extraction SoP

The Monash Autism/ADHD Genetics and Neurodevelopment (MAGNET) Project

**Principal investigators**

Dr Beth Johnson, Prof Mark Bellgrove

Turner Institute of Brain and Mental Health

Monash University

Date: August 2020

Revision: 3

**Confidential**

This document is confidential. It may not be transmitted, reproduced, published, or used without prior written authorization.

**Statement of Compliance**

This document is a protocol for a research project. This study will comply with this protocol, the conditions of the ethics committee approval, and the NHMRC National Statement on ethical Conduct in Human Research (2018).

**Contents**

[Saliva collection using ORAgene-DNA Kits 4](#_Toc44680538)

[Purpose and application 4](#_Toc44680539)

[Risk minimisation 4](#_Toc44680540)

[Portable saliva sample collection kit contents: 4](#_Toc44680541)

[Procedure 5](#_Toc44680542)

[Spill procedure 6](#_Toc44680543)

[Transport of saliva samples to the Bellgrove Lab 6](#_Toc44680544)

[At-home saliva collection 6](#_Toc44680545)

[ORAgene-DNA Saliva Kit Instructions 7](#_Toc44680546)

[If posted out to families: 7](#_Toc44680547)

[Savlia collection using ORAcollect SWAB Kits 8](#_Toc44680548)

[Purpose and application 8](#_Toc44680549)

[Risk minimisation 8](#_Toc44680550)

[Portable saliva sample collection kit contents 8](#_Toc44680551)

[Procedure 9](#_Toc44680552)

[Spill procedure 10](#_Toc44680553)

[Transport of saliva samples to the Bellgrove Lab 10](#_Toc44680554)

[At-home saliva collection 10](#_Toc44680555)

[ORAcollect Saliva Kit Instructions 11](#_Toc44680556)

[If posted out to families: 11](#_Toc44680557)

[DNA Extraction from saliva samples 12](#_Toc44680558)

[Purpose and application 12](#_Toc44680559)

[Risk minimisation 12](#_Toc44680560)

[DNA extraction: from saliva collected using oragene-dna kits 13](#_Toc44680561)

[Items required 13](#_Toc44680562)

[GENERAL TIPS 13](#_Toc44680563)

[Procedure 13](#_Toc44680564)

[Spill clean-up procedure 14](#_Toc44680565)

[Sample Storage 14](#_Toc44680566)

[Items required 15](#_Toc44680567)

[GENERAL TIPS 15](#_Toc44680568)

[Procedure 15](#_Toc44680569)

[Spill clean-up procedure: 16](#_Toc44680570)

[Sample Storage: 16](#_Toc44680571)

[Checking dna quantity and quality using nanodrop 17](#_Toc44680572)

[Items required 17](#_Toc44680573)

[GENERAL TIPS 17](#_Toc44680574)

[Procedure 17](#_Toc44680575)

[Spill clean-up procedure 18](#_Toc44680576)

[Sample Storage 18](#_Toc44680577)

# Saliva collection using ORAgene-DNA Kits

## Purpose and application

This procedure is for the collection of saliva samples from participants using ORAgene DNA kits (OG-500). If children are unable to produce enough saliva, see next section (Saliva collection using ORACollect for Paediatrics)

## Risk minimisation

Antigens in saliva are at a much lower concentration compared to blood, however all saliva must be assumed to be infectious. The main health concerns for researchers collecting saliva is the transmission of hepatitis, herpes class of viruses (herpes simplex, mononucleosis), HIV, influenza, COVID-19 and the common cold, however potential transmission is greatly reduced by following a few simple procedures to reduce exposure. All researchers involved in saliva collection are required to have a Hepatitis B vaccination and must be trained before collecting saliva from participants.

To minimise risk of contamination with biologicals and potential transmission of infectious substances (i.e., COVID-19):

- Wear protective nitrile/latex gloves.
- Clean all working surfaces before and after use with disinfectant wipes.
- Maintain 1.5 m social distancing with other research team members/participants.
  - *IMPORTANT: This extends to 2.0 m social distancing with participants when they are in the process of collecting a saliva sample.*
- Wash hands thoroughly with soap and water before and after saliva collection.
- Avoid touching face/skin or rubbing eyes.
- Do not come to campus if you are experiencing COVID-19 symptoms/or have come in contact with a person who has a confirmed/probable case of COVID-19. Ensure to:
  - Seek medical attention.
  - Contact senior staff member/ work partner.
- If you begin to feel sick while on campus, immediately seek medical attention at the Monash Health Services, located in the Campus Centre and contact senior staff member.

## Portable saliva sample collection kit contents:

- ORAgene Saliva kits (OG-500; see Figure 1)
- Disposable latex gloves
- Plastic cups
- Permanent marker for labelling tubes
- Sticky tape or rubber bands
- Antibacterial/ethanol wipes
- Disinfectant handwash
- Plastic biohazard bags for disposal of waste items (e.g., used gloves, wipes)
- Rigid plastic container, labelled with a biohazard sticker, for temporary storage of saliva samples
- Rigid plastic container, labelled with a biohazard sticker, for temporary storage of plastic biohazard bags containing waste items


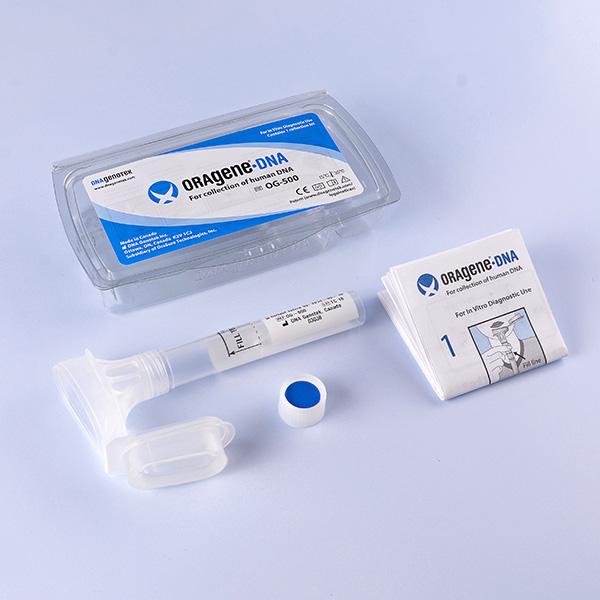


*Figure 1.* ORAgene-DNA saliva kit.

## Procedure

1. Half an hour prior to collecting the sample, provide the participant with a cup of water and ask them to rinse out their mouth, in order to remove any excess food or remnants. *NOTE: The participant will need to wait at least half an hour after drinking the water before they collect the saliva sample*. The participant should be told the following:
   1. *“Please take a drink of water, washing your mouth out as best as possible*, *to remove any food remnants in your mouth. In half an hour, I will ask you to provide a saliva sample so that we can test your DNA. In the meantime, please do not eat or drink anything.”*
2. Before giving the collection tube to the participant, use a permanent marker to write the participant ID on the side of the collection tube and on the lid of the Oragene-DNA plastic outer container.
3. Put on latex gloves.
4. Wherever possible, minimise your exposure to biological hazards. This is done by remaining at a 2 m distance, and having the *participant* handle the saliva kit throughout the procedure. *The parent must assist their child in this procedure*. The participants should be told the following:
   1. “*Please spit into this small tube trying to avoid spitting bubbles.*
   2. *Once you have filled the tube up to the line shown on this picture (indicate the line on the picture) you may stop.*
   3. *Then, snap the lid shut to release the preservative, which helps to maintain the standard of the sample.*
   4. *Now, unscrew the lid and place it in the biohazard bag/bin.*
   5. *Take the small cap, which is provided in the Oragene-DNA plastic outer container, and screw it on tightly*.
   6. *Mix the solution as best as possible for at least 10 seconds.*
   7. *Then, place the tube inside the Oragene-DNA plastic outer container and close the lid.”*
5. Ask the participant to place the Oragene-DNA plastic outer container, containing the sample, into the rigid plastic storage box (the secondary container) labelled with a biohazard sticker.
6. Wipe area using disinfectant wipes.
7. Dispose of latex gloves and other waste in a biohazard bin. If a bin is not immediately available, temporarily store waste in a biohazard plastic bags and place it in a rigid plastic storage box until you are able to dispose of it.
8. Use disinfectant handwash (researcher and participant) or soap and water to clean hands thoroughly.


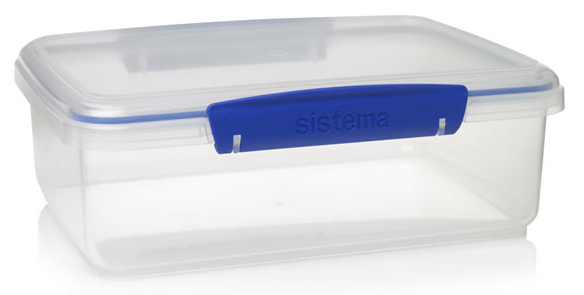


*Figure 2.* Example of rigid, sealed plastic storage container. Must have biohazard sticker displayed on lid and side.

## Spill procedure

1. Any spills during this procedure are likely to be small (4-5ml). Wear gloves. Thoroughly wipe down all surfaces with disinfectant wipes.
2. After cleaning/disinfecting the area, remove your gloves and ensure that you thoroughly wash your hands with soap and water.
3. If saliva makes skin contact, thoroughly wash the affected area under the tap for 10 minutes.
4. Dispose of all gloves and disinfectant wipes: this can be stored temporarily in a bag in the rigid biohazard container before disposal in a biohazard bin.

## Transport of saliva samples to the Bellgrove Lab

1. Store samples in the rigid, sealed plastic container in a cool, dry place. These samples are shelf-stable for up to 5 years. When the plastic container is full, this container can be used to transport all samples to/from the main campus.
2. Transport of biological samples requires that the sample must be double contained: the saliva has a rigid primary container (the saliva kit) and rigid secondary container (the plastic storage container). This rigid secondary container MUST be marked with a biohazard sticker on the lid and side. Please also ensure there is a label indicating the study name and lab group to which these samples relate.
3. All samples are stored (inside secondary containers) in the Bellgrove PC2 lab. *NOTE: Only authorised personnel can enter this area, so if necessary, ask the lab manager for assistance with storing the samples.*

## At-home saliva collection

1. In some cases, participants may need to collect their saliva sample at home (e.g. if parent 2 does not attend the research visit, or when we undertake school visits which parents do not attend). Saliva kits can be sent to participants via mail. Mail-out saliva kits include:
   1. Pre-labelled saliva kits, a reply-paid envelope addressed to the MAGNET office, instructions to collect the saliva samples using the Oragene-DNA kit (see next page), and a hard copy of the explanatory statement and consent form. The participant can follow the instructions to complete this at home, seal it, and then send it back.

# ORAgene-DNA Saliva Kit Instructions

**Please do not consume any food or drink (including water) for 30 minutes before providing the saliva sample.**

| 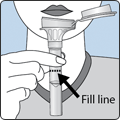 | 1. Spit into funnel until the amount of liquid saliva (not bubbles) reaches the fill line shown in Picture 1. |
| --- | --- |
| 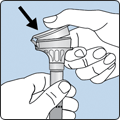 | 2. Hold the tube upright with one hand. Close the funnel lid with the other hand (as shown in Picture 2) by firmly pushing the lid until you hear a loud click. The liquid in the lid will be released into the tube to mix with the saliva. Make sure that the lid is closed tightly. |
| 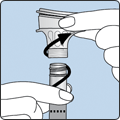 | 3. Hold the tube upright. Unscrew the funnel from the tube. The funnel and plastic ORAgene-DNA outer container can be placed into a bin. |
| 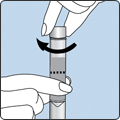 | 4. Use the small cap to close the tube tightly. |
| 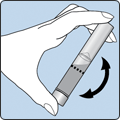 | 5. Invert the capped tube for 5-10 seconds. Place all tubes into the hard, plastic container provided. This does not need to be refrigerated. |

## If posted out to families:

**Please post saliva sample kits back to us using the reply-paid envelope enclosed.** If you have any questions please do not hesitate to contact the team at [med.magnetproject@monash.edu](mailto:med.magnetproject@monash.edu).

# Savlia collection using ORAcollect SWAB Kits

## Purpose and application

This procedure is for the collection of saliva samples from research participants using ORACollect for Paediatrics DNA kits (OC-175). This kit is a reliable alternative to traditional DNA collection methods, and is designed for young donors, offering a non-invasive DNA collection experience that is gentle and fast for both the donor and the person assisting.

## Risk minimisation

Antigens in saliva are at a much lower concentration compared to blood, however all saliva must be assumed to be infectious. The main health concerns for researchers collecting saliva is the transmission of hepatitis, herpes class of viruses (herpes simplex, mononucleosis), HIV, influenza, COVID-19 and common cold, however potential transmission is greatly reduced by following a few simple procedures to reduce exposure. All researchers involved in saliva collection and handling using the ORACollect kit are required to have Hepatitis B vaccinations and must be trained before collecting saliva from participants.

To minimise risk of contamination with biologicals and potential transmission of infectious substances (i.e., COVID-19):

- Wear protective nitrile/latex gloves.
- Clean all working surfaces before and after use with disinfectant wipes.
- Maintain 1.5 m social distancing with other research team members and participants.
  - *IMPORTANT: This extends to 2.0 m social distancing with participants when they are in the process of collecting a saliva sample.*
- Wash hands thoroughly with soap and water before and after saliva collection.
- Avoid touching face/skin or rubbing eyes.
- Do not come to campus if you are experiencing COVID-19 symptoms/or have come in contact with a person who has a confirmed/probable case of COVID-19. Ensure to:
  - Seek medical attention.
  - Contact senior staff member/work partner.
- If you begin to feel sick while on campus, immediately seek medical attention at the Monash Health Services, located in the Campus Centre and contact senior staff member.

## Portable saliva sample collection kit contents

- ORAcollect Saliva kits (OC-175; Figure 1)
- Disposable latex gloves
- Antibacterial/ethanol wipes
- Disinfectant handwash
- Rigid plastic container, marked as biohazard, for temporary storage of saliva samples
- Rigid plastic container, labelled with a biohazard sticker for temporary storage of plastic biohazard bags containing waste items


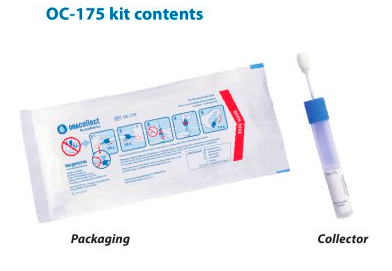


*Figure 1.* ORAcollect saliva kit.

## Procedure

1. Half an hour prior to collecting the sample, provide the participant with a cup of water and ask them to rinse out their mouth in order to remove any excess food or remnants. *NOTE: The participant will need to wait at least half an hour after drinking the water before they collect the saliva sample*. The participant should be told the following:
   1. *“Please take a drink of water, washing your mouth out as best as possible, to remove any food remnants in your mouth. In half an hour, I will ask you to provide a saliva sample so that we can test your DNA. In the meantime, please do not eat or drink anything.”*
2. Before giving the collection tube to the participant, use a permanent marker to write the participant ID on the side of the collection tube.
3. Put on latex gloves.
4. Wherever possible, minimise exposure to biological hazards. This is done by maintaining a 2 m distance and having the *participant* handle the saliva kit throughout the procedure. *The parent must assist their child in this procedure*. The participant should be told the following:
   1. *“Place the sponge comfortably in your mouth and rub your lower gums 10 times back and forth; repeat rubbing motion on the opposite side of the mouth.*
   2. *Holding tube upright, unscrew the cap from the tube.*
   3. *Turn the cap upside down, insert the sponge into the tube and close tightly.*
   4. *Invert the capped tube and shake vigorously 10 times.*
   5. *Now, place the saliva swab back into the plastic packaging and close.”*
5. Ask the participant to place the sample into the rigid plastic storage box (the secondary container) labelled with a biohazard sticker.
6. Wipe up area using disinfectant wipes.
7. Dispose of latex gloves and other waste in a biohazard bin. If a bin is not immediately available, temporarily store waste in a biohazard plastic bag and place it in a rigid plastic storage box until you are able to dispose of it.
8. Use disinfectant handwash (researcher and participant) or soap and water to clean hands thoroughly.


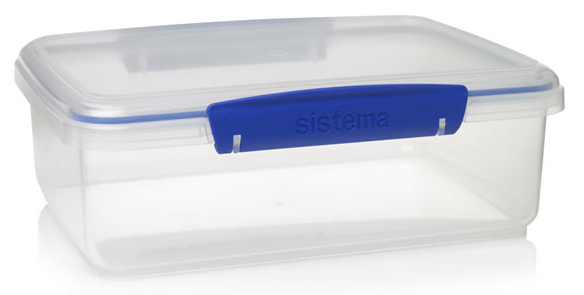


*Figure 2.* Example of rigid, sealed plastic storage container. Must have biohazard sticker displayed on lid and side.

## Spill procedure

1. Any spills during this procedure are likely to be small (4-5ml). Wear gloves. Thoroughly wipe down all surfaces with disinfectant wipes
2. After cleaning/disinfecting the area, remove your gloves and ensure that you thoroughly wash your hands with soap and water.
3. If saliva makes skin contact, thoroughly wash the affected area under the tap for 10 minutes.
4. Dispose of all gloves and disinfectant wipes: this can be stored temporarily in a bag in the rigid biohazard container before disposal in a biohazard bin at either the Monash Biomedical Imaging Centre, Monash Psychology Centre (MPC), or the Bellgrove Lab (Room 511, Building 17, main campus).

## Transport of saliva samples to the Bellgrove Lab

1. **These samples need to be processed within 30 days,** and therefore should be taken to the Bellgrove PC2 lab at the end of each week. IMPORTANT: To ensure samples are processed within the 30 days, notify the Bellgrove team in advance via email, and log the collected sample in the spreadsheet.
2. Transport of biological samples requires that the sample must be double contained: the saliva has a rigid primary container (the saliva kit) and rigid secondary container (the plastic storage container). This rigid secondary container MUST be marked with a biohazard sticker on the lid and side. Please also ensure there is a label indicating the study name and lab group to which these samples relate.
3. All samples are stored (inside secondary containers) in the Bellgrove PC2 lab, located in Room 511, Building 17, Clayton Campus. *NOTE: Only authorised personnel can enter this area, so if necessary, ask the lab manager for assistance with storing the samples.*

## At-home saliva collection

1. In some cases, participants may need to complete their saliva sample at home e.g. if parent 2 does not attend the research visit, or when we undertake school visits which parents do not attend. Saliva kits can be sent to participants via mail. Mail-out saliva kits include:
   1. Pre-labelled saliva kits, a reply-paid envelope addressed to the MAGNET office, instructions to collect the saliva sampling using the ORACollect for Paediatrics kit (see next page), and a hard copy of the explanatory statement and consent form. The participant can follow the instructions to complete this at home, seal it, and then send it back.

# ORAcollect Saliva Kit Instructions

**Please do not consume any food or drink (including water) for 30 minutes before providing the saliva sample.**

| 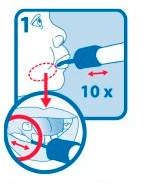 | 1. Place the sponge comfortably in the mouth and rub lower gums 10 times back and forth. |
| --- | --- |
| 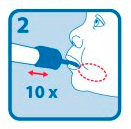 | 1. Repeat rubbing motion on the opposite side of the mouth. |
| 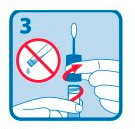 | 1. Holding tube upright, unscrew the cap from the tube. |
| 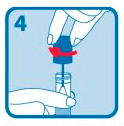 | 1. Turn the cap upside down, insert the sponge into the tube and close tightly. |
| 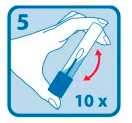 | 1. Invert the capped tube and shake vigorously 10 times. |

## If posted out to families:

**Please post saliva sample kits back to us using the reply-paid envelope enclosed.** If you have any questions please do not hesitate to contact the team at [med.magnetproject@monash.edu](mailto:med.magnetproject@monash.edu).

# DNA Extraction from saliva samples

## Purpose and application

This set of procedures are for the extraction of DNA from participant saliva samples collected using the **ORAgene DNA (OG-500)** and **ORACollect for Paediatrics (OC-175)** kits, and for the quality/quantity assessment of the extracted DNA using a NanoDrop 2000/2000c.

## Risk minimisation

Antigens in saliva are at a much lower concentration compared to blood, however all saliva must be assumed to be infectious. The main health concerns for researchers collecting saliva is the transmission of hepatitis, herpes class of viruses (herpes simplex, mononucleosis), HIV, influenza, COVID-19 and the common cold, however potential transmission is greatly reduced by following a few simple procedures to reduce exposure. All researchers involved in DNA extraction are required to have Hepatitis B vaccinations and must be trained and inducted to the Bellgrove PC2 lab (Room 511, Building 17, Clayton Campus).

To minimise risk of contamination with biologicals and potential transmission of infectious substances (i.e., COVID-19):

- Wear protective lab coat, facemask (if working within 1.5 m of other researcher), nitrile/latex gloves and protective eyewear.
  - This includes when using the computer in the lab!
- Clean all working surfaces before and after use with 80% v/v Ethanol.
- Maintain 1.5 m social distancing for others working in the lab.
- Wash hands thoroughly with soap and water before and after entering the lab.
- Avoid touching face/skin or rubbing eyes.
- Do not come to campus if you are experiencing COVID-19 symptoms/or have come in contact with a person who has a confirmed/probable case of COVID-19. Ensure to:
  - Seek medical attention.
  - Contact senior staff member/ work partner.
- If you begin to feel sick while on campus, immediately seek medical attention at the Monash Health Services, located in the Campus Centre and contact senior staff member.

The number of Monash staff working on campus/in Building 17 can vary from day to day, which can be a potential safety risk. To minimise risk when working on campus **in isolated conditions**:

- Do not work outside of office hours.
- In advance, arrange for a senior staff member to be your contact person (by phone or in person) on the day/s you are working at the university. Provide them with a plan for your day, the expected arrival and departure times and set times to check in with them throughout the day. The senior staff member will also need to be contactable throughout the day in case of an emergency/incident.
- Have your phone fully charged and with you at all times.
- Where possible, work in pairs (while maintaining social distancing requirements) to minimise safety risk.
- Upon arriving at Building 17, Clayton Campus, contact senior staff member to announce arrival.
- Contact Campus Security (9902 7777) to let the know your location on campus and estimated length of time you intend to stay.
- Touch base with the senior staff member at the appointed times.
- If the event of injury, seek treatment from the Monash Health Services, located in the Campus Centre and contact senior staff member.

In the event of serious injury/emergency, contact Campus Security (9905 3333) or triple zero, and senior staff member.

# DNA extraction: from saliva collected using oragene-dna kits

The following procedure is specific to DNA extraction from participant saliva samples collected using the ORAgene-DNA collection kits (OG-500).

## Items required

- Participant saliva samples in ORAgene-DNA collection kits (OG-500)
- Purifying agent (prepIT.L2P)
- Latex/nitrile gloves
- DNA storage buffer: TE
- 200 uL pipette with sterile tips
- 1000 uL pipette with sterile tips
- Eppendorf tubes
- Blue and orange 15 mL tubes
- Tube racks
- 100% ethanol (absolute)
- 70% ethanol (made up using 100% absolute ethanol and distilled water)
- Paper towel
- 80% ethanol (made up using 96% ethanol and distilled water) for cleaning benchtop
- Water bath
- Centrifuge
- Microcentrifuge

## GENERAL TIPS

- Ensure all tubes are labelled correctly
- Clean your bench space prior to, and after, working in the lab.
  - Firstly, spray bench with 80% ethanol (ETOH) and wipe with paper towel
  - Then, spray with distilled water and wipe with paper towel.
- Use your allocated bench space – ask lab manager/supervisor if uncertain
- Blue 15 mL tubes are only good up to 5000 RPM.
- Orange 15 mL tubes are stronger.
- Make sure you are only using the pipettes that lab manager/supervisor allocate.
- Pipette tips and Eppendorf tubes need to be autoclaved prior to use. Ask lab person for assistance with this, if you have not been shown how to use the autoclave.

##

## Procedure

1. Transfer 4000µL saliva sample to 15 ml tube (use blue tubes). Incubate overnight at 50°C. NOTE: If not possible to leave overnight, need 2 hours minimum in bath.
2. Collect ice from Level 2 using a Styrofoam box – (As you step off the lift on Level 2, turn right, and the ice machine is in the room at the end of corridor). NOTE: Sometimes this room is locked on weekends, so ensure you have access. Do not wear lab coat/gloves.
3. Add 40µL ORAgene purifier per 1000µL to each tube. Mix by vortexing for a few seconds.
4. Incubate samples for 10 minutes on ice.
5. Centrifuge for 5 minutes at 5000 RPM at room temperature (NOTE: blue tubes are OK for use at 5000 RPM).
6. Set up fresh 15 ml tube (use orange tubes) and carefully add (in any order):
   1. The clear supernatant with a pipette tip into the tube. Discard the tube containing the pellet.
   2. Per 1000µL supernatant, add 1000µL 100% ethanol (ETOH) (e.g., for 4 mL supernatant add 4 mL 100% ethanol).
7. Invert the tube and allow the sample to stand at room temperature for 10 minutes to allow the DNA to fully precipitate.

| **If a clear DNA pellet has formed:** | **If a clear DNA pellet has not formed:** |
| --- | --- |
|  | Place samples in -20C freezer for 2-3 hours. |
|  | Centrifuge for 15 min at 9,500 RPM at room temperature. |
| Use a 1000uL pipette (set to 100 uL) to transfer it to a 1.5 ml microtube (Eppendorf tube). *Note: you will probably transfer a small amount of liquid along with the DNA pellet*. | Check if pellet has formed:   - If yes, go to next step. - If not, leave sample in -20C freezer overnight. Next day centrifuge sample at room temperature for 15 min at 9500. Then go to next step. |
| Add 1 mL 70% Ethanol to the pellet. | Discard supernatant. Take care to avoid disturbing the DNA pellet. |
| Centrifuge at room temperature for 5 min at 9,500 RPM. | To the pellet, add 5 mL 70% ETOH. |
| Discard liquid. Take care to avoid disturbing the DNA pellet. | Centrifuge at room temperature for 5 mins at 9500 RPM. |
|  | Discard liquid. Take care to avoid disturbing the DNA pellet. |
| Cover lid of the 1.5 ml tube with parafilm, pierce the surface of the parafilm with a needle and allow to dry in fume hood for 1-2 hours (NEED to check that pellet is dry before step 8). | Cover the lid of the tube with parafilm, pierce the surface of the parafilm with a needle and allow to dry for 1-2 hours (NEED to check that pellet is dry before step 8) |

1. Add 100µL of TE buffer to dissolve the dried DNA pellet. Close lid and place in the fridge. You may need to increase the amount of TE depending on the size of the pellet.
   1. To make TE: Use special highly purified water (ask lab manager/supervisor), 49.5ml of purified water with 0.5 concentrated TE (found in fridge)
2. Flick the tubes periodically every couple of hours to ensure all of the DNA is dissolved. Once dissolved, place in -20°C freezer.

## Spill clean-up procedure

- Any spills are likely to be less than 5 mL. Wear minimum PPE required for PC2 lab (latex gloves, lab coat, protective glasses). Thoroughly wipe down all surfaces with 80% ethanol v/v ethanol and paper towel.
- After cleaning/disinfecting the area, remove your gloves and ensure that you thoroughly wash your hands with soap and water.
- If saliva/DNA makes skin contact, thoroughly wash the affected area under the tap for 10 minutes.

## Sample Storage

- DNA samples in Eppendorf and orange 15 mL tubes can be stored for the short-term in the fridge located in the Bellgrove PC2 lab (Level 5, Building 15, Clayton Campus). Short-term storage may be appropriate when you need access to the samples. Ensure all samples are labelled appropriately and contained in labelled secondary container or tube rack.
- DNA samples in Eppendorf and orange 15 mL tubes can be stored long term in the -80C freezer located in the Bellgrove PC2 lab (Level 5, Building 15, Clayton Campus). Ensure all samples are labelled appropriately and contained in labelled secondary container.

DNA Extraction: from saliva collected using oracollect for paediatrics kits

The following procedure is specific to DNA extraction from participant saliva samples collected using the ORACollect for Paediatric (OC-175) kits.

### Items required

- Participant saliva samples in ORAgene-DNA collection kits (OC-175)
- Latex/nitrile gloves
- DNA storage buffer: TE
- Purifying agent (prepIT.L2P)
- 1.5 mL microtubes
- Tube racks
- 100% ethanol (absolute)
- 70% ethanol (made up using 100% absolute ethanol and distilled water)
- Paper towel
- 80% ethanol (made up using 96% ethanol and distilled water) for cleaning benchtop
- Water bath
- Microcentrifuge

### GENERAL TIPS

- Ensure all tubes are labelled correctly
- Clean your bench space prior to, and after, working in the lab.
  - Firstly, spray bench with 80% ethanol (ETOH) and wipe with paper towel
  - Then, spray with distilled water and wipe with paper towel.
- Use your allocated bench space – ask lab supervisor (e.g., lab manager/supervisor) if uncertain
- Make sure you are only using the pipettes that lab manager/supervisor allocate.
- Pipette tips and microtubes need to be autoclaved prior to use. Ask lab person for assistance with this, if you have not been shown how to use the autoclave.
- The laptop in the lab is not connected to the internet, so you will need to bring a USB to transfer the data (this is relevant when you are checking the quantity and quality of extracted DNA in the samples).

### Procedure

1. Mix the sample in the collection tube by inversion and gentle shaking for a few seconds. This is to ensure the sample is properly mixed.
2. Incubate overnight at 50°C in the original collection tube for at least two hours (ensure the sample remains submerged). Preferably incubate overnight.
3. Collect ice from Level 2 using a Styrofoam box – (As you step off the lift on Level 2, turn right, and the ice machine is in the room at the end of corridor). NOTE: Sometimes this room is locked on weekends, so ensure you have access. Do not wear lab coat/gloves.
4. Transfer 500 uL of the mixed sample to a 1.5 mL microcentrifuge tube. The remainder of the sample can be stored at room temperature or frozen (-15°C to -20°C).
5. For 500 uL of sample, add 20 uL of purifier to the microcentrifuge tube and mix by vortex for a few seconds.
6. Incubate samples for 10 minutes on ice.
7. Centrifuge for 5 minutes at 12,500 RPM at room temperature.
8. Set up fresh 1.5 mL microcentrifuge tubes and carefully add (in any order):
   1. The clear supernatant with a pipette tip into the tube. Discard the tube containing the pellet.
   2. To 500 uL of supernatant, add 6000 uL of 100% ethanol (ETOH) (e.g., for 4 mL supernatant add 4 mL 100% ethanol).
9. Invert the tube and allow the sample to stand at room temperature for 10 minutes to allow the DNA to fully precipitate.
10. Place the tube in a *known* orientation inside the microcentrifuge - this will help to locate the DNA pellet. Centrifuge at room temperature for 2 minutes at 12,500 RPM.
11. Carefully remove the supernatant with a pipette tip and discard it, taking care not to disturb the DNA pellet. *TIP*: Rotating the tube such that the pellet is on the upper wall will allow you to safely move the pipette along the lower wall and remove the supernatant. The supernatant contains impurities, so try to remove as much as possible.
12. Carefully add 250 uL of 70% ethanol to the tube and let stand for 1 minute. **Completely** remove all ethanol without disturbing the pellet. Any leftover ethanol may impact the performance of the assay. *TIP*: If the pellet detaches, centrifuge the sample for 5 minutes.
13. Add 100µL of TE buffer to dissolve the dried DNA pellet. Vortex for at least 5 seconds.
14. To ensure complete rehydration of the DNA, incubate overnight at room temperature, followed by vortexing, or at 50°C for 1 hour with occasional vortexing.
    1. To make TE: Use special highly purified water (ask lab manager/supervisor), 49.5ml of purified water with 0.5 concentrated TE (found in fridge).
15. Flick the tubes periodically every couple of hours to ensure all of the DNA is dissolved. Once dissolved, place in -20°C freezer.

### Spill clean-up procedure:

- Any spills are likely to be less than 1 mL. Wear minimum PPE required for PC2 lab (latex gloves, lab coat, protective glasses). Thoroughly wipe down all surfaces with 80% ethanol v/v ethanol and paper towel.
- After cleaning/disinfecting the area, remove your gloves and ensure that you thoroughly wash your hands with soap and water.
- If saliva/DNA makes skin contact, thoroughly wash the affected area under the tap for 10 minutes.

### Sample Storage:

- DNA samples in microcentrifuge tubes can be stored for the short-term in the fridge located in the Bellgrove PC2 lab. Short-term storage may be appropriate when you need access to the samples. Ensure all samples are labelled appropriately and contained in labelled secondary container or tube rack.
- DNA samples in microcentrifuge tubes can be stored long term in the -20C freezer located in the Bellgrove PC2 lab. Ensure all samples are labelled appropriately and contained in labelled secondary container.

# Checking dna quantity and quality using nanodrop

The following procedure is to check the quantity and quality of DNA extracted from the saliva samples.

### Items required

- NanoDrop 2000/2000c (Thermo Scientific)
- Latex/nitrile gloves
- DNA storage buffer: TE (you will use this as your blanking sample)
- Distilled water
- 10 uL pipette with sterile tips
- 2uL pipette with sterile tips
- Tube racks/box for samples
- Kim Wipes
- Laptop

### GENERAL TIPS

- Ensure the DNA is dissolved in your sample, otherwise, you won’t get an accurate reading.
- Clean your bench space prior to, and after, working in the lab.
  - Firstly, spray bench with 80% ethanol (ETOH) and then distilled water.
  - Wipe with paper towel.
- Use your alternate hand as a support when pipetting liquid onto the eye of the NanoDrop, as it helps to keep your hand steady.
- Make sure you are only using the pipettes that lab manager/supervisor allocate.
- Pipette tips need to be autoclaved prior to use. Ask lab person for assistance with this, if you have not been shown how to use the autoclave.

### Procedure

1. Clean the NanoDrop:
   1. Using a 10uL pipette, place 3-4 uL of distilled H2O on the eye of NanoDrop.
   2. Close the NanoDrop. Open it. Wipe with Kim wipes
2. On laptop, open the NanoDrop 2000 application
   1. Click YES. The following page will appear:


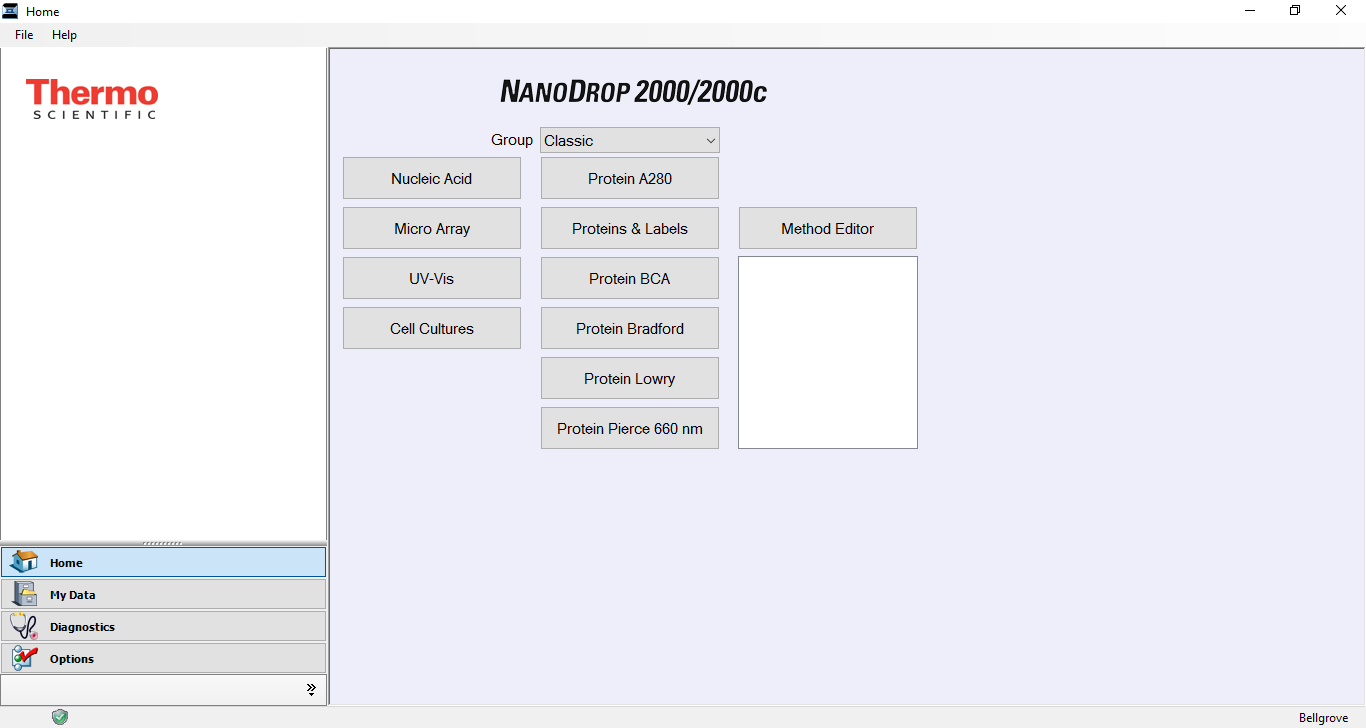


- 1. Click “Nucleic Acid”. The following screen will appear:


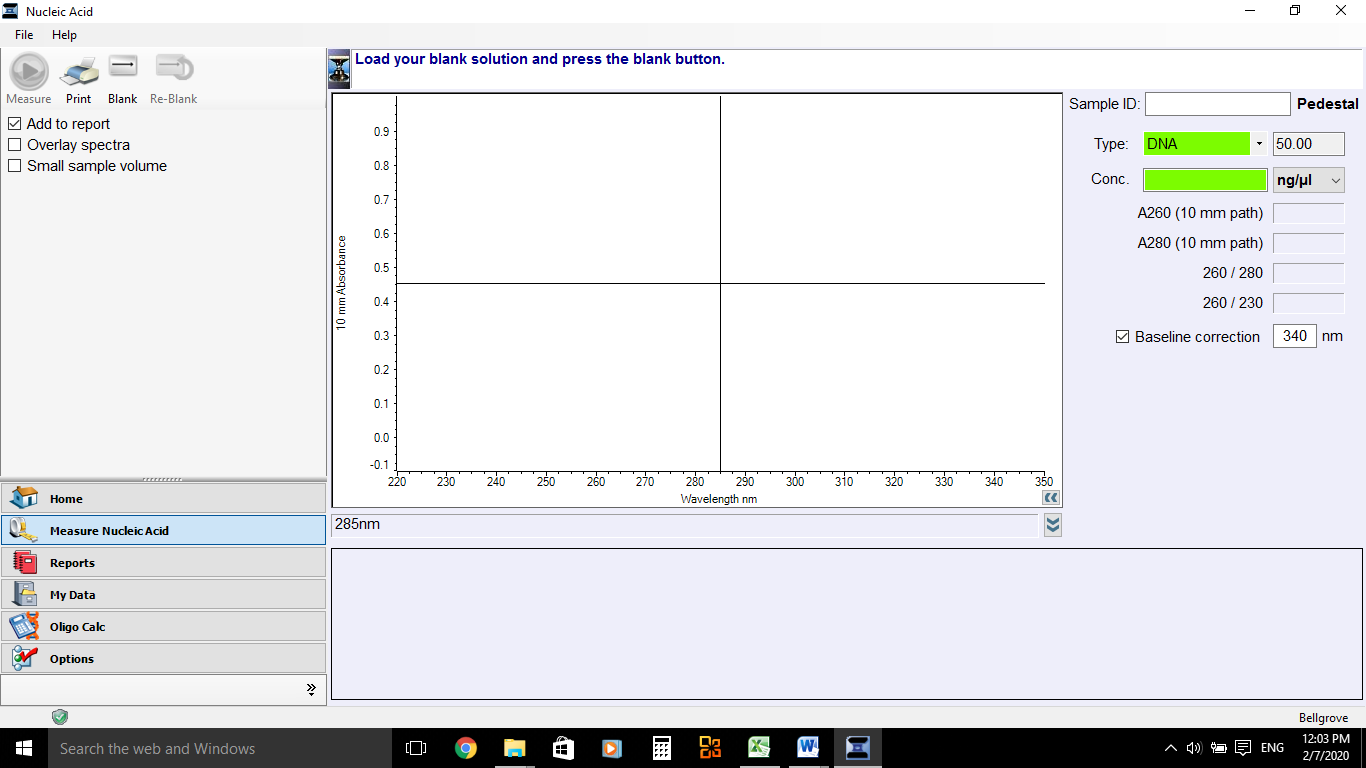


- 1. Click OK to allow for wavelength verification (ensure lid is close for this).
  2. Load blanking sample:
     1. Using a 2 uL pipette, place 1 uL of TE (your blanking solution) on the eye.
     2. Click “BLANK”, located in top left corner of screen.
     3. Wipe top and bottom eye with Kim Wipe.
  3. Load samples:
     1. Using a 2 uL pipette, place 1 uL of sample on eye.
     2. Type Sample ID in the “Sample ID” field, located in top right-hand corner. (IMPORTANT: include identifying characters for study, too e.g., MP XXXX).
     3. Click “Measure”. A graph will appear, and the concentration in ng/uL will appear in the “Conc.” field.
     4. NOTE: 260/280 provides an understanding of the clean/high quality DNA extraction. Ideally, the reading should give OD = 1.8, although acceptable limits are between 1.6 – 2.2.
  4. You can extract the data for each sample by clicking “Reports”, which is located in the bottom left of the screen. Save an export of the data with the project name and date.

### Spill clean-up procedure

- Any spills are likely to be less 1 mL. Wear minimum PPE required for PC2 lab (latex gloves, lab coat, protective glasses). Thoroughly wipe down all surfaces with 80% ethanol v/v ethanol and paper towel.
- After cleaning/disinfecting the area, remove your gloves and ensure that you thoroughly wash your hands with soap and water.
- If DNA makes skin contact, thoroughly wash the affected area under the tap for 10 minutes.

### Sample Storage

- DNA samples in Eppendorf and orange 15 mL tubes can be stored for the short-term in the fridge located in the Bellgrove PC2 lab. Short-term storage may be appropriate when you need access to the samples. Ensure all samples are labelled appropriately and contained in labelled secondary container or tube rack.
- DNA samples in Eppendorf and orange 15 mL tubes can be stored long term in the -80C freezer located in the Bellgrove PC2 lab. Ensure all samples are labelled appropriately and contained in labelled secondary container.

# 
